# Slow and steady wins the race: The behaviour and welfare of commercial faster growing broiler breeds compared to a commercial slower growing breed

**DOI:** 10.1371/journal.pone.0231006

**Published:** 2020-04-06

**Authors:** Laura M. Dixon

**Affiliations:** Animal Behaviour and Welfare, SRUC, Edinburgh, United Kingdom; Tokat Gaziosmanpasa University, TURKEY

## Abstract

Broilers have been bred for fast growth which has led to welfare problems such as high mortality, lameness and skin lesions. Slower growing breeds are thought to have better welfare but are not as efficient in production. This study investigated welfare, behaviour, production and meat quality of faster growing broilers from three main commercial broiler companies (breeds FA, FB and FC) with a commercially available slower growing breed (Hubbard JA757, S). Four hundred birds of each breed (total 1600 birds) were reared in pens of 50 birds, 8 per breed (total 32 pens). Home pen behaviour was recorded once a week in hourly scan samples to get behavioural time budgets. Welfare Assessments (WA) were done when the average bird weight per breed was 2.2 and 2.5kg. Birds and feed supplied were regularly weighed by pen and Feed Conversion Ratio (FCR) and Average Daily Gain (ADG) were calculated at 2.2kg. Birds were then slaughtered and meat quality measures were taken. S and FC had lower mortality and culls due to lameness (P<0.05 for both). Breeds FA, FB and FC grew faster, ate less feed and had better FCR and ADG (P<0.05 for all). S had scores indicating higher welfare for the majority of WA measures and spent more time active and less time sitting, feeding and drinking than the other breeds (P<0.05 for all). Faster growing breeds had more breast meat and S had more leg meat; although S had better meat quality scores (P<0.05). Overall, S birds have improved welfare in terms of activity and welfare measure scores compared to the other breeds but take longer to reach slaughter weight and are not as efficient in production measures. However if lower mortality and improved meat quality are taken into account, as well as the premium price paid for these birds, slower growing broilers may be a viable commercial option.

## Introduction

Poultry are currently the highest consumed meat worldwide and there are over 66 billion birds being used for meat production each year [1]. In the UK alone, there are over 1 billion broilers (*Gallus gallus domesticus*) producing over 1.6 million tonnes of meat each year with approximately 94% of these birds being a faster growing breed which are slaughtered at 5–6 weeks of age, weighing 2.2–2.5 kg [2]. The fast growth rate and weight gain in fast growing broilers is known to be associated with a number of health and welfare issues (e.g. reviewed by [3–4]). Animal welfare encompasses normal biological functioning, emotional state and the ability to express certain normal behaviour patterns [5]. As broilers age and grow, they have increased levels of lameness and decreased levels of activity which are thought to be a main cause of leg weakness [3]. Gait scoring systems have been developed to assess lameness levels (e.g. scale of 0–5, [6]) and birds with higher gait scores (≥3) are thought to be in pain [7]. Lameness levels vary with production and management systems but occurrences of gait score three or higher have been found in 30% of birds in Denmark [8], in 57% of birds in the Netherlands, Britain, Belgium and Italy [9] and in 27.6% of UK farms [10] while only between 1–2.2% of birds have perfect gaits (score = 0; [9–10]). However, most of this research was collected 9+ years ago and breeding companies do select against leg disorders which means the current commercial values may be lower than what is found in peer reviewed literature.

The increased lameness and associated decreased activity levels have the added effect of the birds spending more time sitting. Where litter quality is poor, this increases the occurrences of skin lesions, like hock burn, foot pad dermatitis (FPD) and breast blisters which are also thought to be painful in severe cases [11]. Additionally, faster growing broilers have high mortality levels compared to breeds not selected as heavily for growth, can suffer from metabolic disorders like ascites and are prone to heat stress as they age (e.g. [12–13]). An additional welfare issue that stems from the use of faster growing broilers is chronic hunger in the parent stock: Broilers are slaughtered before they are sexually mature, therefore separate flocks of sexually mature birds are used to produce the broiler chicks. These birds are called broiler breeders and they have similar genetic potential as their offspring to grow and put on weight quickly. However, since they need to live a lot longer than broilers (60+ weeks) and have good reproductive abilities, they are feed restricted up to 1/3 of what they would chose to eat during rearing to sexual maturity (~20 weeks, reviewed by [14–15]). This leads to birds which are physically healthy but show abnormal behaviour and frustration as a result of chronic hunger (Broiler Breeder Paradox, [16]).

The use of slower growing broiler breeds has been suggested to decrease welfare issues. For example, previous studies found that slower growing breeds are more active, have lower levels of lameness and fewer hock and foot pad lesions than faster growing breeds [17–18]. They have lower mortality rates and fewer lameness issues resulting in fewer culls. However they also take longer to reach slaughter weight, often 52–81 days compared to 35–42 days in faster growing broilers (sales market, breed and housing system dependent) and they consume more feed to reach the same weight which is one of the major costs of broiler production. These breeds also tend to be incorporated into higher welfare schemes, meaning an increased price for the product [19]. Additionally, the broiler breeder hens of the slower growing broilers are also slower growing or dwarf breeds, meaning they can consume more feed while still maintaining a slower growth rate and good production parameters (e.g. [20]), suggesting that they might suffer less from chronic hunger induced by food restriction.

Based on the known welfare issues of faster growing broilers, the major breeding companies have been including health and welfare parameters into their breeding programs of faster growing broilers to reduce issues such as lameness and ascites and to decrease mortality rates [21–23]. Although improvements in animal welfare can improve productivity, this is not always the case and some behavioural improvements (e.g. increased activity) may have negative consequences for production (e.g. increased feed intake and/or taking longer to reach slaughter age) and more refined genetic selection may need to take place [24]. Additionally, bird growth rates for the faster growing breeds are still increasing and fast growth appears to be the major contributor to most broiler welfare problems [3] making it unclear how much of a difference to bird welfare the breeding programs for these breeds are making.

Despite the improvements found in the behaviour and welfare of slower growing breeds, there is limited research comparing faster and slower growing breeds side by side within the same environment, and of the existing studies, some are over a decade old [17–18] or only focus on meat quality parameters (e.g. [25]) while others are based in free range systems (e.g. [26]) which only make up a small percentage of broiler production. Broiler breeding companies do make and use their own comparisons, but this data is generally not available for assessment. Therefore the aim of this research was to compare the welfare, behaviour and production of commonly used faster growing breeds from the three major broiler producers with a commercially established slower growing breed housed indoors. It was hypothesised that the slower growing breed will have better scores in the welfare assessments (such as lower gait and hock burn scores) and will perform more active behaviour patterns (such as locomotion and foraging) than faster growing breeds. However faster growing breeds will have better production measures (such as lower FCR) and will consume less feed than the slower growing breed.

## Material and methods

### Ethical concerns

These trials were approved by the Animal Welfare and Ethics Review Body at SRUC. Birds were inspected a minimum of 3 times per day. Any birds found to be unwell were closely monitored and given appropriate veterinary treatment as necessary or, if their welfare had significantly decreased, culled.

### Birds and housing

1600 birds in total from four breeds of broilers (400 of each) were used, spread over two replicates. The Hubbard JA757, a slower growing breed, was used as the Control (S) and one of the most extensively used breeds from each of the top three broiler producing companies (Ross 308, Cobb 500, Hubbard Flex) were used as the Treatments (for anonymity, these were designated in no particular order as breeds FA, FB and FC).

For each replicate, 200 day of hatch chicks from each breed were collected from commercial hatcheries and transported to SRUC’s Avian Science Research Centre (Scotland). Here chicks were weighed and distributed into pens (one breed per pen) As Hatched (no chick sexing was done). There were 4 pens of 50 birds of each breed (16 pens of 50 birds), spread over two rooms (8 pens per room, 2 pens of each breed per room). Within each room, 2 blocks were formed, each containing one pen of each breed (total of 4 blocks). The placement of pens in the blocks was done using an incomplete Latin Square Design. This gave a total number of 400 birds per breed (1600 total) and 8 pens per breed (32 pens).

Each pen measured 1.97 x 3.00 m and contained a 5cm deep layer of wood shavings, a manual pan feeder, a bell drinker and a 1.3 m long x 20cm high perch as per the RSPCA Broiler Breed Welfare Assessment Protocol [27] with a stocking density of 8.5 birds/m^2^. Wood shavings were replenished as needed to keep them dry and friable. This was needed 3–5 times (averaging an extra 24kg of wood shavings per pen) throughout the trial for breeds FA, FB and FC and 2–3 times (averaging an extra 14 kg wood shavings) for the S. The feeder, drinker and perch were in the same location in each pen, with the perch towards the back of the pen and the feeder and drinker towards the front.

Birds were provided *ad libitum* access to water for the duration of the trial. Birds were fed *ab libitum* for the entire trial, except for feed withdrawal 12 hours before slaughter. Feed was standard commercial broiler formulations, provided from Target Feeds (Scotland) as chick Starter crumbs from days 0–10 (protein level = 22.25%, ME = 13.15 MJ/kg), Grower pellets from days 11–28 (protein level = 21.27%, ME = 13.10 MJ/kg) and Finisher pellets from day 29 to the end of the trial (protein level = 18.20%, ME = 13.35 MJ/kg).

For days 0–2, birds had a two hour continuous dark period from 12:00–02:00, this increased by one hour per day up until it reached six hours of darkness (18L:6D) which was maintained for the duration of the trial. Light was provided by overhead LED bulbs enclosed in protective plastic covers. Lux levels were an average of 50 lux per pen and the wavelength mix was from 420-780nm. Temperature and relative humidity were measured for each pen on a daily basis and followed commercial broiler recommendations. The breed standards for each breed were obtained and consulted throughout the trial to ensure that birds were growing and eating as expected. The rearing conditions that were used came from a higher welfare scheme in order to maintain our obligation as a research institute to providing environments that are thought to promote better welfare conditions while still being commercially relevant.

### Recorded measures

#### Pen weights

Birds were bulk weighed by pen at days 0, 14, 28, 35 and 42 days of age. The slower growing S birds were also bulk weighed by pen at 49 and 56 days of age.

#### Mortality

All incidences of birds found dead or which had to be culled because of deteriorating health to prevent suffering were recorded and the reason for the death/cull was recorded on a per pen basis.

#### Welfare assessment (WA)

Welfare assessments were done when the average bird weight for a breed was 2.2kg and again at 2.5 kg. The average bird weight per breed was determined from the bulk pen weights, from using the breed growth standards and average daily gain. As the birds approached the welfare assessment weights, a random sub-sample of 10 birds per pen were weighed daily to accurately determine the dates the welfare assessment should be performed. On average, faster growing broilers reached 2.2kg at 35 days of age and 2.5kg at 38 days of age and S birds reached 2.2kg at 48 days of age and 2.5kg at 54 days of age. One person assessed all the measures to avoid inter-observer variability.

All measures were collected following the protocols in the RSPCA Broiler Welfare Assessment Protocol [27], and included the following components for all birds:

Weight and Sex—Birds were individually weighed and their sex recorded at the time of each WA. The average male:female ratio ± SEM was 0.96 ± 0.090 for the faster growing broilers (or in other words, for every 20 female birds, there were 19 males) and 1.18 ± 0.093 for S (for every 20 females, there were 24 males) and there was no significant difference between the four breeds using mixed model analyses (F_3, 5_ = 3.28, P = 0.12).

Gait Score—All birds were gait scored during a welfare assessment. Each bird was placed individually on the floor 2m from a pen with other chickens in it and was allowed to walk towards the pen. Gait was allocated a score using the RSPCA Broiler Breed Welfare Assessment Protocol walking ability guidelines (Table 1).

**Table 1:** Gait scores and their descriptions^a^.

Score	Description
**0**	The bird displays smooth, fluid locomotion. Typically the foot is picked up and put down smoothly and each foot is brought under the bird's centre of gravity as it walks (rather than the bird swaying). Often, the toes are partial curled while the foot is in the air.
**1**	The bird has a slight defect in its gait that is difficult to define precisely. The bird may take unduly large strides, be unsteady or wobble when it walks, which produces an even gait, but the problem leg is unclear/cannot be easily identified.
**2**	The bird has a definite and identifiable gait abnormality, but this does not affect its ability to move. The bird may take short, quick, unsteady steps with one leg, but is not sufficiently lame to seriously compromise its ability to move, i.e. manoeuvre, accelerate and run.
**3**	The bird has an obvious gait defect that affects its ability to move. The bird may have a limp, jerky or unsteady strut, or splay one leg as it moves. The bird often prefers to squat when not coerced to move, and will not run.
**4**^b^	The bird has a severe gait defect. The bird is capable of walking, but only with difficulty and when driven or strongly motivated. Otherwise it squats down at the first available opportunity.
**5**^b^	The bird is incapable of sustained walking on its feet. Although it may be about to stand, the bird cannot walk except with the assistance of the wings or by crawling on the shanks.

^a^ Taken from the RSPCA Broiler Breeds Welfare Assessment Protocol [27], Appendix 1, Table 1.

^b^ Any birds with a Gait Score of 4 or 5 were culled.

25 birds per pen had additional measures collected and were chosen using a pre-selected randomisation scheme that identified these birds in the order they were caught (1–50) for Gait Scoring. For example, if birds 3 and 7 were pre-identified as requiring additional measures, these birds represented the 3^rd^ and 7^th^ bird that were caught for the Gait Score measurements.

These 25 birds per pen were given a Feather Cover Score, Breast Feather Cleanliness Score, Foot Pad Dermatitis Score, and Hock Burn Score (Tables 2–4).

**Table 2:** Feather cover score and their descriptions^a^.

Score	Definition
**0**	Feather cover is full and even over body and wings
**0.5**	Feather cover is slightly patchy on the sides OR back of body OR on the wings
**1**	Feather cover is patchy to bare on sides OR back of body
**1.5**	Feather cover is bare on sides of body with a light covering on back
**2**	Body is bare of feathers and wings are patchy of feathers

^a^Taken from the RSPCA Broiler Breeds Welfare Assessment Protocol [27], Appendix 1, Table 2.

**Table 3:** Breast cleanliness feather score and descriptions^a^.

Score	Definition
**0**	Plumage is clean
**1**	Slightly dirty plumage
**2**	Large patches of dirty plumage on breast/breast is completely covered in dirty plumage

^a^Taken from the RSPCA Broiler Breeds Welfare Assessment Protocol [27], Appendix 1, Table 3.

**Table 4:** Foot pad dermatitis and hock burn scores and definitions^a^.

Score	Foot pad dermatitis score definitions	Hock burn score definitions
**0**	No lesions present on the pads.	No discoloration or lesions present on hocks.
**0 (P/S/H)**	Very small superficial lesions (1-2mm), slight discolouration in a limited area, mild hyperkeratosis OR no lesions present on the pads, but the pad is pink (P) and/or swollen (S) and/or scarred (i.e. pads have a new, smooth skin covering—healed (H)).	Very small and superficial (<1mm), slight discolouration in a limited area, mild hyperkeratosis (thickening of the skin) OR no discoloration or lesions present on hocks, but hock is pink (P) and/or swollen (S).
**1**	Area affected does not extend over the entire plantar pad, substantial discolouration, dark papillae, superficial lesion, and no ulceration.	Area affected does not extend over hock, substantial discolouration, dark papillae, superficial lesion, and no ulceration.
**2**	Greater surface of plantar pad usually affected, sometimes with lesions on toes. Deeper lesion/s with ulceration, sometimes haemorrhage, scabs of significant size, severely swollen foot pad.	Greater surface of hock affected. Deeper lesion/s with ulceration, sometimes haemorrhage, scabs of significant size, severely swollen area.

^a^Taken from the RSPCA Broiler Breeds Welfare Assessment Protocol [27], Appendix 1, Tables 5 and 6.

#### Behaviour

All pens were video recorded for one day (24-hours) once a week. Scan sampling was done once per hour for the 24-hour period each week, giving 24 scans per 24 hours per pen per week. As some of the behaviours (see below) couldn’t be interpreted through a still video image, for each ‘scan’ the video was played for 10 seconds, then rewound and the same 10 seconds were reviewed again and this was repeated for each behaviour noted. This sampling method provides a reasonable approximation of daily time budgets when compared to periods of continuous behaviour recording [28]. The samples were divided into the lights on and lights off periods and summarised to give overall proportions of behaviour patterns during each. The birds spent the majority of the lights off period sitting/resting so only the lights on behaviour will be presented. The behaviour patterns recorded were feeding: bird with head in feeder, drinking: bird with head in drinker, standing: upright position with two feet on the ground and legs extended and no other activity being performed, sitting: breast in contact with the ground and no other activity being performed, locomotion: moves more than 2 steps in one direction, foraging: scratching or digging in a substrate with the beak or feet, perching: standing or sitting on the perch, preening: using beak to clean feathers or body and dustbathing: lying on the ground and rubbing body in the litter or moving the litter over the body with the feet, beak or wings.

#### Production measures

Feed consumed—The amount of feed provided to each pen was recorded on a daily basis. Feed weigh backs were done when switching between Starter, Grower and Finisher diets, at feed withdrawal before slaughter and just before the first welfare assessment (average bird weight per breed 2.2kg) for each pen. Due to mortalities, there were not always 50 birds left in a pen so to standardise it, this data was converted to feed (kg) per 50 birds per pen for analyses.

Feed Conversion Ratio (FCR) and Average Daily Gain (ADG)—FCR (kg feed intake per kg weight gain) and ADG (mean weight gain per day in kg/day) were calculated for each breed when the average bird weight per breed was 2.2kg.

#### Slaughter measures

Birds were individually wing tagged a few days prior to slaughter and live weight of the birds per pen was recorded directly before the birds went to slaughter. In order for live bird weights before slaughter to not be statistically different between the slower and faster growing breeds, the faster growing breeds were slaughtered at 42 days of age and the slower growing breeds were slaughtered at 60 days of age. After slaughter, eviscerated carcass weight, abdominal fat weight, leg weights and breast weights were recorded, and the following indicators of poor meat quality were scored:

Breast Striations (White Striping)—Each breast was assessed on a 3 point scale: 0 = no striping, 1 = moderate degree of striping, 2 = severe degree of striping (adapted from [29]).

Wooden Breast—Each breast was assessed on a 2 point scale: 0 = absence of wooden breast, 1 = presence of wooden breast (adapted from [30]).

#### Statistical analyses

For all score data, the proportion of male and female birds per pen that had a specific score was calculated and used for analyses. For the behaviour data, the proportion of birds performing a behaviour during the lights on and lights off periods for each day was calculated and used for analyses, however only the behaviour for the lights on period is presented here due to the majority of the lights off period being spent sitting (resting/sleeping).

Data were analysed using Mixed Model analyses in Genstat (16^th^ ed.). Normality of residuals was tested after model fitting and where necessary data were transformed to improve normality. Factors included in the model were Replicate, Room, Block, Pen Nested in Breed as random effects, and the fixed effect was Breed. For the welfare assessment measures, Sex and WA number (1 or 2) were also included as fixed effects and for the behaviour and bird weight measures, Bird Age was included as a fixed effect. Interactions of Breed, Bird Age, Replicate and Block were included; additionally interactions of Sex and WA were included for the welfare assessment measures. The statistical tests used a significance level of 5% and were based on approximate *F* tests referencing the observed *F* statistics to the *F* distribution when available. Otherwise Wald tests were used referencing the Wald statistics to the Chi-Squared distribution. If the data could be not be transformed until residuals were appropriately normal, non-parametric statistical tests were used. This was only needed for foot pad dermatitis (Kruskal-Wallis) and abdominal fat weight (Mann-Whitney).

Data is presented as either raw means and standard error of the means (SEM) or back transformed means to show the biological significance of the results.

## Results

Replicate, Room, and Block did not have significant effects on the results (P>0.05) and will not be presented here. Interactions that were not significant (P>0.05) are also not included in the below results.

### Pen weights

**Fig 1:**
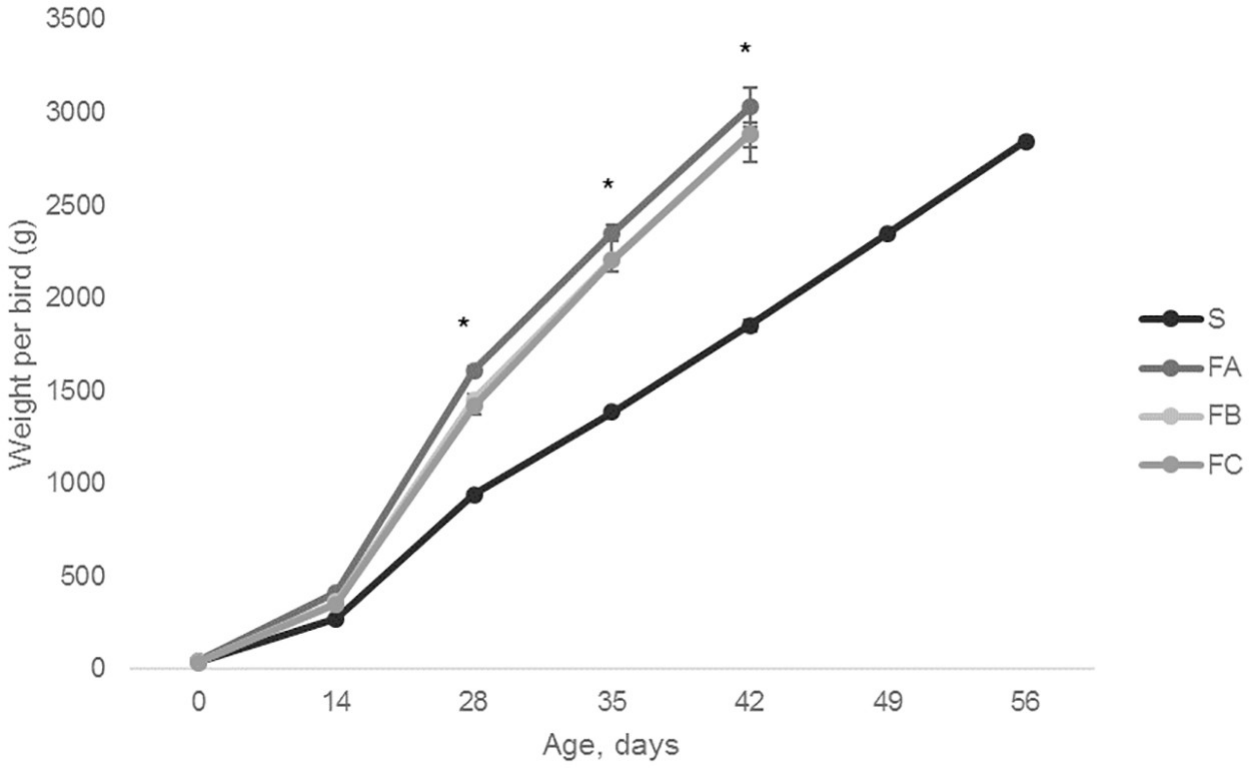
Mean bird weights (± SEM) per breed over time. Asterisk symbols denote signicant differences (P<0.05).

As expected, S birds grew more slowly than FA, FB and FC after about 14 days of age (F_12, 107_ = 2.09, P = 0.023, Fig 1).

### Mortality

The reasons for mortality (including both birds that were found dead and birds that had to be culled on welfare grounds) were yolk sac infections (32%), lameness (24%), bird unwell/unresponsive (13%), found dead—cause undetermined (12%), flip over (9%), injury (5%) or small/runt cull (5%).

S and FC had lower overall mortality rates (birds found dead + culls) than FA or FB (Mean proportion ± SEM, S: 0.05 ± 0.01 and FC: 0.07 ± 0.02 vs FA: 0.11 ± 0.01 and FB: 0.11 ± 0.02; F_3, 17_ = 7.14, P = 0.003). S and FC also had lower proportions of birds culled due to lameness than FA and FB (Mean proportion ± SEM, S: 0.01 ± 0.005 and FC: 0.002 ± 0.002 vs FA: 0.04 ± 0.005 and FB: 0.03 ± 0.01; F_3, 10_ = 4.93, P = 0.025). More birds were found dead during the first 2 weeks of age while more birds were culled from 3 weeks of age until slaughter with the exception of the S breed which had fewer birds found dead and culled after 3 weeks of age compared to the first 2 weeks of age (Table 5).

**Table 5:** The percentages of birds that were either found dead or culled during the first two weeks of age and from three weeks of age to slaughter for the different breeds.

Breed	Age range (weeks)	% f.d.	% cull
**S**	0–2	2.09	1.40
**S**	3- slaughter	0.70	0.93
**FA**	0–2	2.84	1.90
**FA**	3- slaughter	1.42	4.50
**FB**	0–2	4.72	2.12
**FB**	3- slaughter	0.47	3.54
**FC**	0–2	4.05	0.95
**FC**	3- slaughter	0.71	1.67

### Welfare Assessment (WA)

#### Weight and sex

There was no significant difference in the weights of male or female faster and slower growing birds for WA 1 or 2 (P = 0.16). Overall, male birds of all breeds were significantly heavier than female birds (mean ± SEM, Males: 2577 ± 25g, Females: 2207 ± 18g; F_1, 96_ = 5.54, P = 0.021). As mentioned in the Methods, the faster growing broilers reached 2.2kg at an average of 35 days of age and 2.5kg at an average of 38 days of age and S birds reached 2.2kg at 48 days of age and 2.5kg at 54 days of age.

#### Gait score

**Fig 2:**
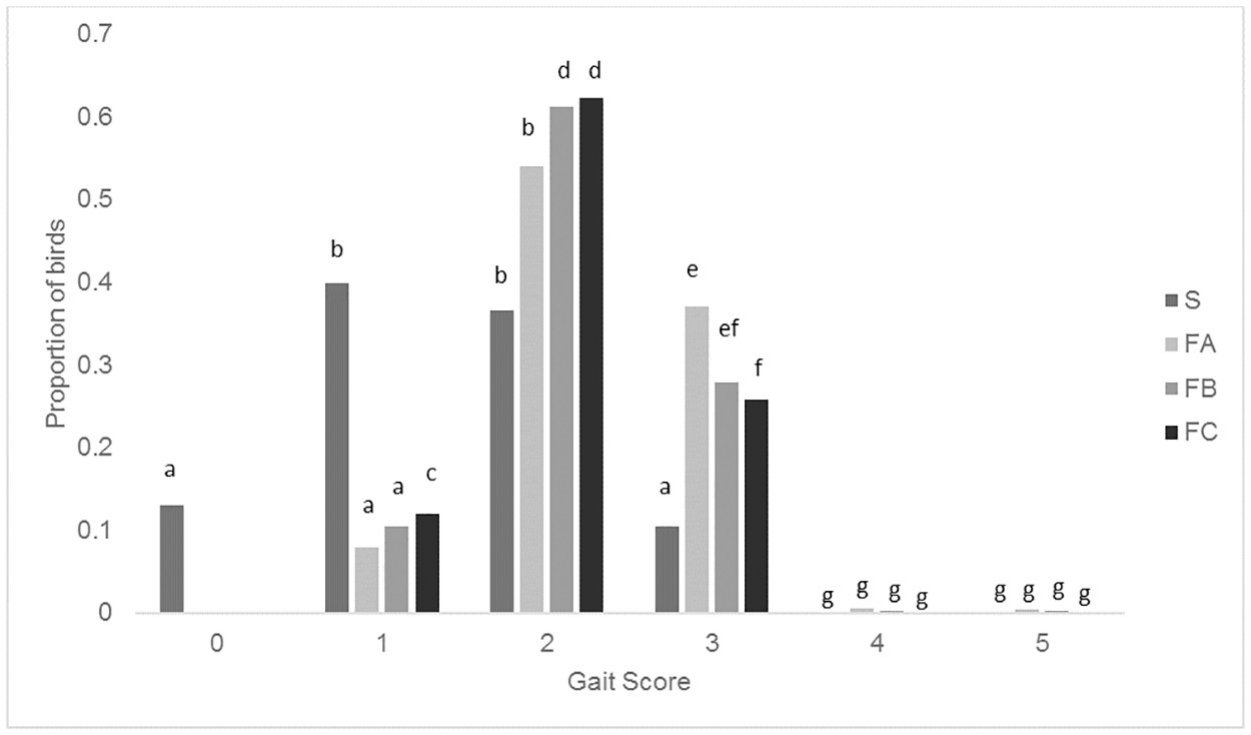
Mean back-transformed proportions of each gait score for the four breeds. Different letters denote signicant differences (P<0.05).

The proportion of birds with higher gait scores increased (gait worsened) as the birds aged (WA 1 vs 2: Wald_5_ = 22.24, P = 0.001) and there was a larger proportion of males with higher average gait scores than females (Wald_5_ = 113.16, P<0.001) (Table 6). S birds had a higher proportion of lower gait scores than the other breeds and FC had more GS1 and fewer GS3 than FA and FB (Wald_15_ = 22.24, P<0.001) (Fig 2).

**Table 6:** The mean proportions (SEM) of birds which had each gait score for sex and welfare assessment.

	SEX		WA	
GS	F	M	1	2
**0**	0.059 (0.017)^a^	0.0058 (0.0028)^b^	0.046 (0.015)^a^	0.019 (0.0092)^a^
**1**	0.25 (0.023)^c^	0.10 (0.020)^d^	0.22 (0.024)^b^	0.13 (0.022)^c^
**2**	0.62 (0.033)^e^	0.45 (0.023)^f^	0.57 (0.026)^d^	0.50 (0.033)^e^
**3**	0.071 (0.010)^d^	0.43 (0.033)^e^	0.16 (0.21)^c^	0.35 (0.039)^b^
**4**	0.0014 (0.00095)^b^	0.0029 (0.0014)^b^	0.0030 (0.0014)^f^	0.0013 (0.00090)^f^
**5**	0.0012 (0.00085)^b^	0.0028 (0.0014)^b^	0.0040 (0.0016)^f^	0 (0)^f^

Different letters denote signicant differences (P<0.05).

#### Feather cover score

Males had a larger proportion of poorer feather scores than females. In particular, males had a larger proportion of feather score 1.5 than females (Score 1.5: Mean proportion ± SEM, Males: 0.10 ± 0.02, Females: 0.03 ± 0.009, F_4, 544_ = 5.14, P<0.001).

**Fig 3:**
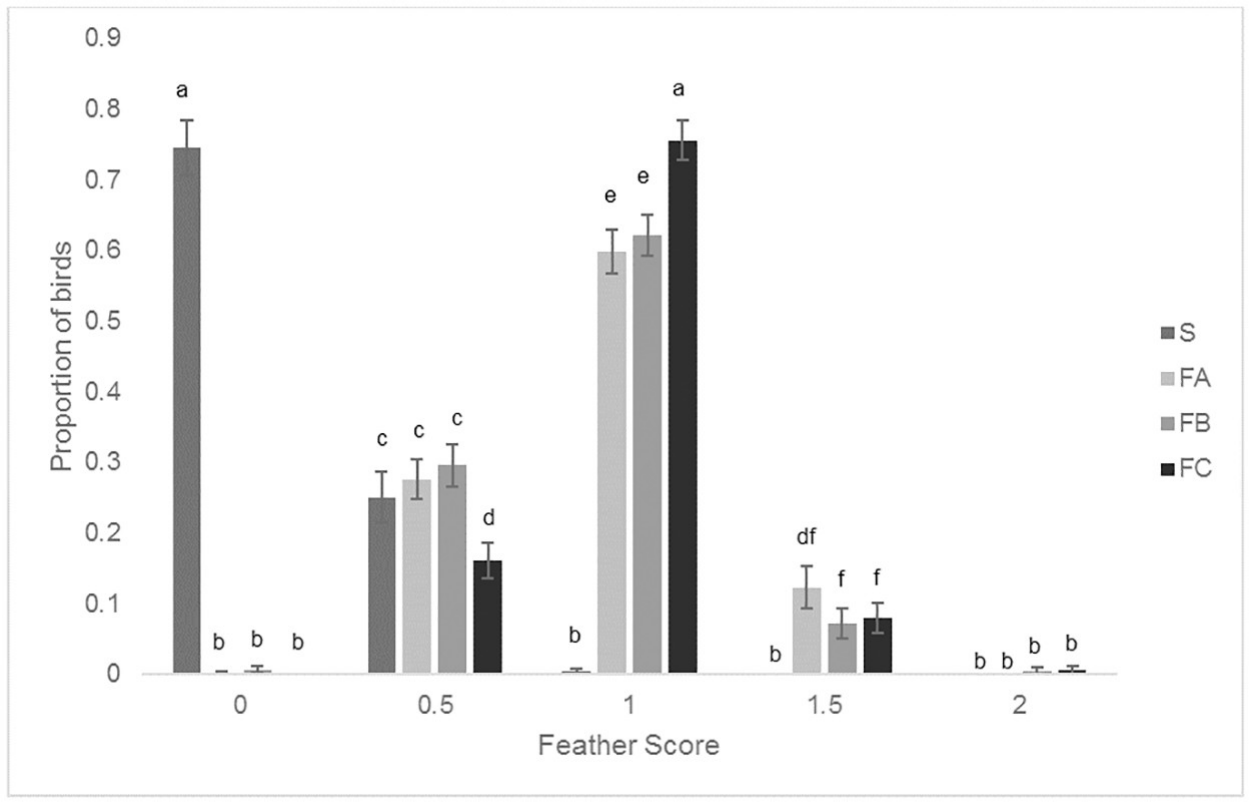
Mean proportions (± SEM) of the different feather scores for the four breeds. Different letters denote signicant differences (P<0.05).

S birds had significantly higher proportions of lower (better) feather scores than the other breeds and FC birds had higher proportions of higher (worse) feather scores than the FA and FB birds (F_12, 544_ = 153.23, P<0.001) (Fig 3).

#### Breast feather cleanliness score

Overall, males had a larger proportion of higher (worse) breast cleanliness scores than females, specifically they had fewer score 1’s and more score 2’s than females indicating dirtier breasts (F_6, 320_ = 6.88, P = 0.001) and the proportions of higher (worse) scores increased between the first and the second WA (F_2, 320_ = 37.52, P<0.001) (Table 7).

**Table 7:** The mean proportions of birds (SEM) which had each breast feather cleanliness score for sex and welfare assessment.

	SEX		WA	
Breast Feather Cleanliness Score	F	M	1	2
**0**	0.078 (0.021)^a^	0.074 (0.019)^a^	0.078 (0.019)^a^	0.074 (0.020)^a^
**1**	0.28 (0.029)^b^	0.21 (0.023)^c^	0.32 (0.027)^b^	0.16 (0.022)^c^
**2**	0.65 (0.039)^d^	0.72 (0.035)^e^	0.60 (0.034)^d^	0.76 (0.038)^e^

Different letters denote signicant differences (P<0.05).

**Fig 4:**
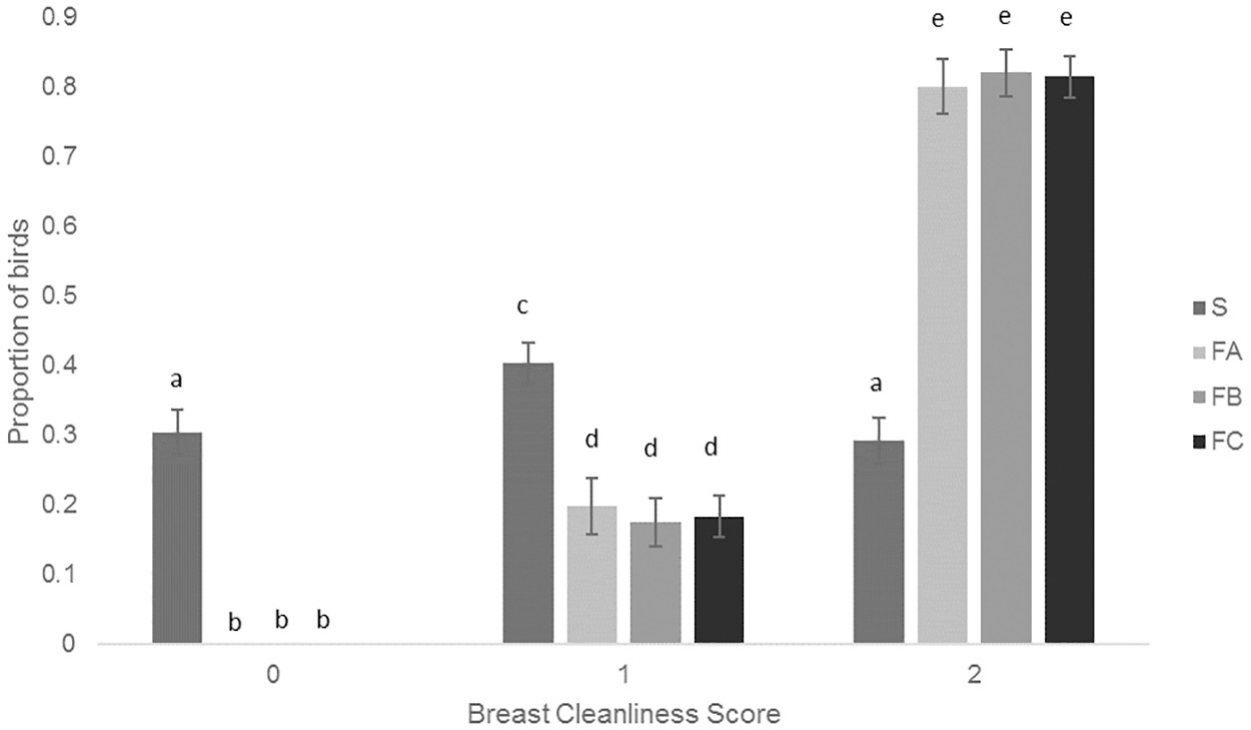
Mean proportions (± SEM) of the different breast cleanliness scores for the four breeds. Different letters denote signicant differences (P<0.05).

S birds had a significantly higher proportion of lower (better) breast cleanliness scores than the other breeds (F_12, 544_ = 153.23, P<0.001; Fig 4).

#### Foot pad dermatitis (FPD)

Levels of FPD were low (97% of birds had a Score 0) and there were no significant differences in the proportions of different FPD scores between the different breeds (F^2^_(3)_ = 1.11, P = 0.774).

#### Hock burn

**Fig 5:**
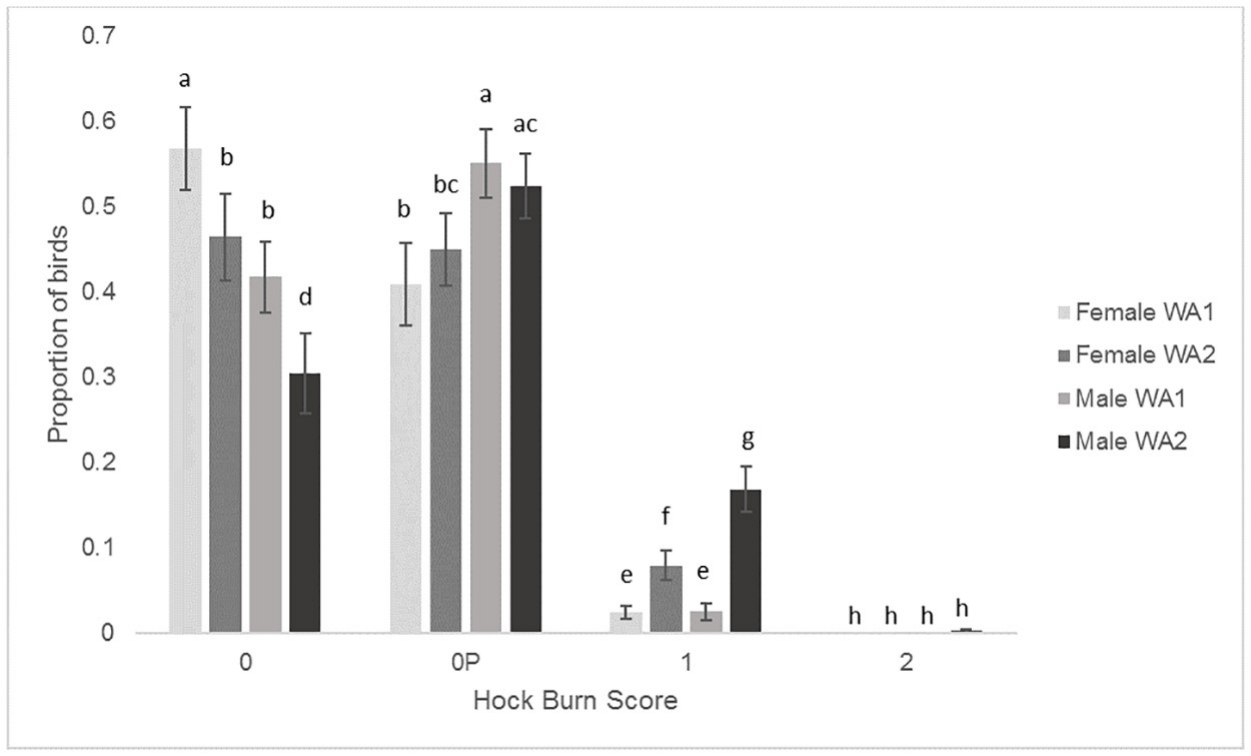
Mean proportions (± SEM) of the different hock burn scores for female and male birds during welfare assessment (WA) 1 and 2. Different letters denote signicant differences (P<0.05).

Males had larger proportions of higher (worse) hock burn scores than females, with fewer 0 scores and more 0P and 1 scores (F_3, 432_ = 32.56, P<0.001) and the proportions of higher scores increased over time, with higher scores in second WA compared to the first (F_3, 432_ = 18.55, P<0.001) (Table 8). There was a Sex by WA interaction with both females and males having more score 0’s and fewer score 1’s in WA1 compared to WA2 but for score 1 males also had higher (worse) scores compared to both WA1 and 2 scores for females (Fig 5).

**Table 8:** The mean proportions of birds (SEM) which had each hock burn score for sex and welfare assessment (WA).

	SEX		WA	
Hock Burn Score	F	M	1	2
**0**	0.52 (0.035)^a^	0.36 (0.032)^b^	0.49 (0.033)^a^	0.38 (0.036)^b^
**0P**	0.43 (0.032)^b^	0.54 (0.027)^a^	0.48 (0.033)^a^	0.49 (0.028)^a^
**1**	0.052 (0.010)^c^	0.097 (0.017)^d^	0.025 (0.0061)^c^	0.12 (0.017)^d^
**2**	0 (0)^e^	0.0016 (0.0011)^e^	0 (0)^e^	0.0016 (0.0011)^e^

Different letters denote signicant differences (P<0.05).

**Fig 6:**
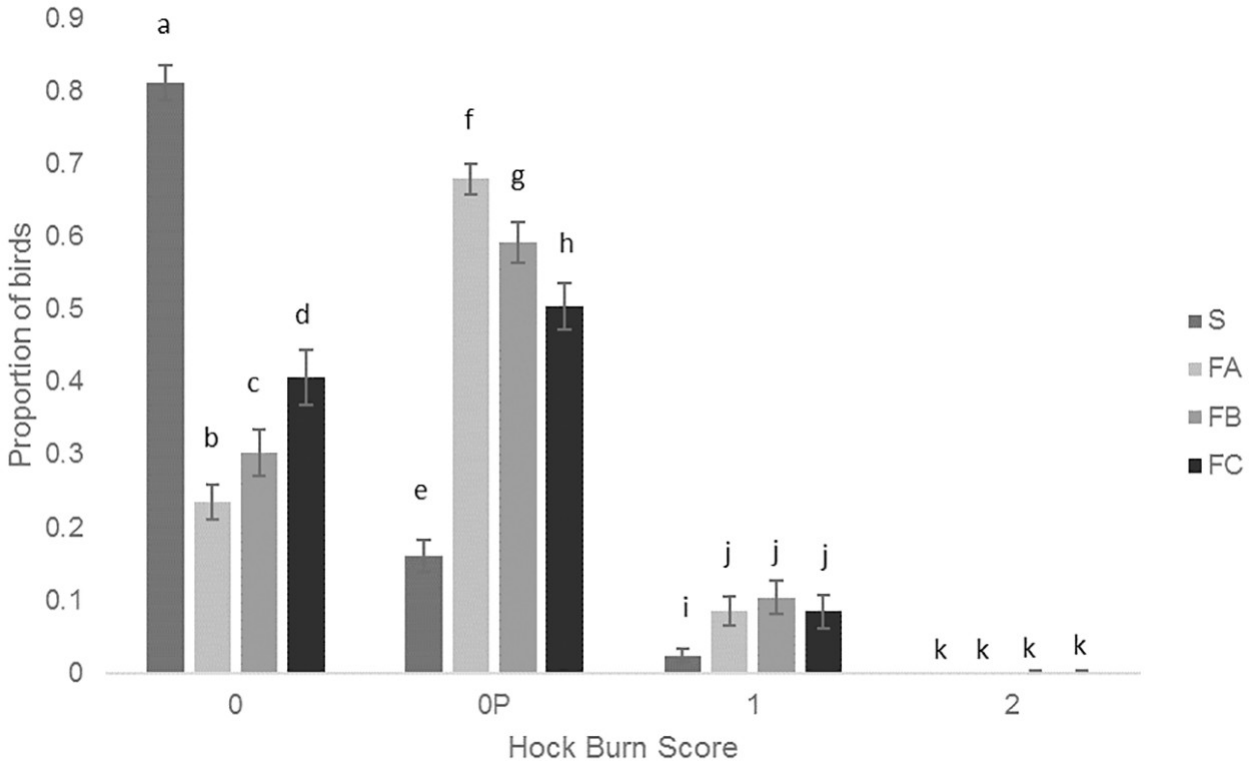
Mean proportions (± SEM) of the different hock burn scores for the four different breeds. Different letters denote signicant differences (P<0.05).

S birds had larger proportions of lower (better) hock burn scores than the other breeds and FC birds had larger proportions of lower (better) hock burn scores than FA and FB and FB had larger proportions of lower (better) hock burn scores than FA (F_9, 432_ = 102.72, P<0.001) (Fig 6).

### Behaviour

**Fig 7:**
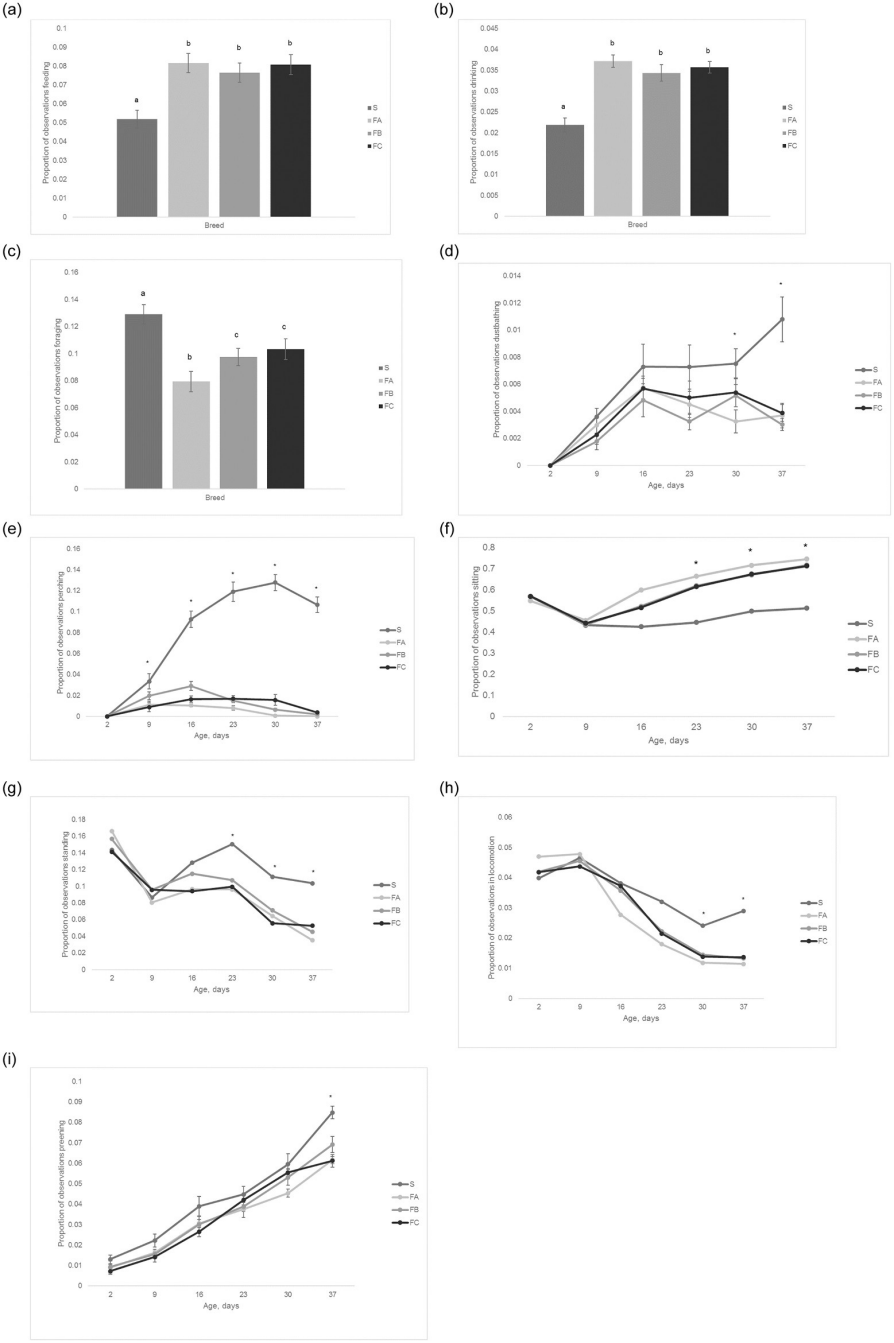
The mean proportions of time spent a) feeding (± SEM), b) drinking (± SEM), c) foraging (± SEM), d) dustbathing (± SEM), e) perching (± SEM), f) sitting (back transformed), g) standing (back transformed), h) in locomotion (back transformed), and i) preening (± SEM) during the lights on period for the four breeds. Different letters and asterik symbols denote signicant differences.

S birds spent less time feeding, drinking and sitting than the other breeds and more time standing, in locomotion, foraging, preening, dustbathing and perching (P<0.01 for all). For sitting, standing, locomotion, perching, preening and dustbathing, the differences between S and the other breeds mainly occurred later in life when the faster growing breeds increased the amount of time they spent sitting and decreased their time spent on other behaviour patterns (P<0.01 for all; Fig 7).

### Production measures

S birds consumed more feed overall (per 50 birds = 268.37 kg vs FA: 226.39kg, FB: 220.08kg, FC: 212.43kg); however they consumed less of the Starter and Grower diets and more Finisher diet than the other breeds (F_6, 58_ = 2.80, P = 0.019; Table 9).

**Table 9:** The mean (SEM) amounts of feed consumed in the different diet phases, feed conversion ratio and average daily gain for the four breeds.

	Diet Phase				
Breed	Starter (kg)^a^	Grower (kg)^a^	Finisher (kg)^a^	FCR^b^	ADG (g/day)^b^
**S**	11.34 (0.31)^a^	61.76 (1.26)^c^	195.30 (1.51)^f^	1.76 (0.014)^a^	46.08 (0.27)^a^
**FA**	15.44 (0.32)^b^	99.21 (1.71)^d^	111.74 (4.65)^g^	1.46 (0.014)^b^	63.49 (0.35)^b^
**FB**	13.30 (0.23)^b^	91.34 (1.00)^de^	115.44 (2.77)^g^	1.43 (0.025)^b^^c^	62.61 (0.56)^b^
**FC**	12.4 (0.28)^a^^b^	86.28 (1.42)^e^	113.75 (2.92)^g^	1.35 (0.013)^c^	62.75 (0.37)^b^

Different letters denote signicant differences (P<0.05).

^a^All values are the mean amount per 50 birds (kg)

^b^FCR and ADG were calculated when the mean bird weight per breed was 2.2kg (at WA1)

FCR and ADG were calculated at the first WA when the average bird weight per breed was 2.2kg. S birds had higher FCR (F_3, 12_ = 6.66, P = 0.007) and lower ADG rates (F_3, 17_ = 5.71, P = 0.007) than the other breeds (Table 9).

### Slaughter measures

There was no significant difference in live bird slaughter weight between the four breeds (P = 0.11). FB had lighter eviscerated carcass weights than the other breeds (F_3, 19_ = 6.78, P = 0.003), S birds had more abdominal fat (U: 0.0, P = 0.004) and heavier leg weights than the other breeds (F_3, 12_ = 45.39, P<0.001) and S and FB birds had lighter breast weights than the other breeds (F_3, 10_ = 5.97, P = 0.013) (Table 10).

**Table 10:** The mean (SEM) eviscerated carcass, abdominal fat, breast weights and leg weights for the four breeds.

Breed	Eviscerated Carcass Weight (g)	Abdominal Fat (g)	Breast Weights (g)	Leg Weights (g)
S	2181 (153.53)^a^	73.94 (1.48)^a^	520.7 (29.47)^a^	699 (59.66)^a^
FA	2085 (81.25)^a^	35.46 (0.85)^b^	655.8 (23.56)^b^	620.1 (24.75)^b^
FB	1916 (65.3)^b^	35.67 (1.033)^b^	553.1 (17.14)^a^	604.3 (22.87)^b^
FC	1995 (64.47)^a^	33.93 (0.54)^b^	609.9 (17.66)^b^	620.5 (22.62)^b^

Different letters denote signicant differences (P<0.05).

There were also sex effects on slaughter measures: females had lighter eviscerated carcass weight than males (mean ± SE, females: 1830 ± 18g, males: 2219 ± 35g, F_3, 19_ = 6.78, P = 0.003), had lighter leg weights than males (mean ± SE, female: 560 ± 6g, males: 694 ± 13g, F_3, 11_ = 16.25, P<0.001) and lighter breast weights than males (mean ± SE, female: 545 ± 13g, males: 644 ± 16g, F_3, 10_ = 5.97, P = 0.013).

#### Breast striations (white striping)

S birds had better meat quality in terms of a larger proportion of lower breast striation scores than the other breeds. FC also had more score 0 and fewer score 2’s than FA and FB (F_6, 51_ = 55.67, P<0.001) (Table 11).

**Table 11:** The mean proportions (SEM) of each breast striation score and of each wooden breast score for the four breeds.

	Breast Striation Score			Wooden Breast Score	
Breed	0	1	2	0	1
**S**	0.90 (0.034)^a^	0.087 (0.027)^d^	0.0093 (0.0093)^f^	0.99 (0.0093)^a^	0.0093 (0.0093)^c^
**FA**	0.18 (0.047)^b^	0.63 (0.036)^e^	0.15 (0.034)^b^	0.72 (0.045)^b^	0.23 (0.058)^d^
**FB**	0.21 (0.025)^b^	0.64 (0.030)^e^	0.14 (0.018)^b^	0.96 (0.033)^a^	0.031 (0.012)^c^
**FC**	0.33 (0.048)^c^	0.57 (0.052)^e^	0.063 (0.016)^h^	0.82 (0.12)^b^	0.14 (0.053)^cd^

Different letters denote signicant differences (P<0.05).

#### Wooden breast

S and FB birds had better meat quality in terms of a larger proportion of lower wooden breast scores than FA and FC (F_1, 51_ = 62.17, P<0.001) (Table 11).

## Discussion

The birds grew as expected and met or slightly exceeded the breed standards, with the faster growing breeds all growing at a similar rate to each other and the S breed (JA757) taking 14 days longer to reach a similar weight. Mortalities were higher than recommended/reported for commercial systems, ranging from 5–11%. This is most likely due to the smaller numbers of birds per pen, meaning all dead birds were found and recorded and due to pro-active culling of birds which appeared unwell or had higher gait scores. With the large numbers of broilers in a commercial shed, it is unlikely that under commercial conditions, all birds which are severely lame are found and culled as they were here. Overall S and FC birds had lower mortality levels and fewer birds culled for lameness issues than FA and FB.

S birds had better welfare indicators than the faster growing breeds, including lower gait scores, feather scores, breast cleanliness scores and hock burn scores. Faster growing breeds have been found to have a hock burn incidence of 60% at five weeks of age similar to the faster growing breeds in this study but Kjaer et al [18] found lower levels of hock burn (under 10%) than found here for the slower growing breed despite growing the birds to 10 weeks of age. In this study, the relative humidity was set and maintained at commercial guidelines (placement to 5 days of age: 60–70%, 6–11 days of age: 50–70%, 12 days of age to the end of the trial: 50–60%) so this should not be the cause of S birds having higher hock scores. We used a 4 level scale to measure hock burn while Kjaer et al [18] used a 3 level scale which combined our lowest two categories into one and may account for this difference. Comparing the faster growing breeds, FA birds had worse gait scores and hock burn scores while FC had worse feather scores but better hock burn scores. Gait score in particular is often used to assess broiler welfare with birds having a gait score of 3 or above assumed to have poorer welfare and be in pain/discomfort [7]. It has been reported that faster growing breeds commercially have from 30% [6, 31] to 57% [31] of birds with a gait score of 3 or above while only 17% of slower growing birds have a gait score of 3 or above [32]. In this experiment, the faster growing breeds ranged from 26–37% of birds having gait score 3 and above while 10% of the slower growing S birds had gait score 3 and above. These scores are slightly lower than previously found but this is most likely due to the litter being kept in a dry and friable state through the study and the birds being kept at a lower stocking density in accordance with the RSPCA Broiler Breed Welfare Assessment Protocol requirements. Overall, male birds had worse gait scores, feather scores, breast cleanliness scores and hock burn scores than female birds and the welfare assessment measures tended to get worse for both males and females as the birds aged and grew from 2.2 to 2.5 kg. Other studies have also found male broilers (heavier birds) to have worse welfare than females (e.g. [8]) most likely due to the faster growth rate of males compared to females. Foot pad dermatitis was not significantly different between any of the breeds and was low overall. Other studies have found low levels of FPD in slower growing birds but found levels of 30% at 35 days of age in faster growing breeds [18]. Our low levels of FPD may again be due to the litter being kept in dry and friable and the birds being kept at a lower stocking density. Additionally, when birds were sitting on the litter, they often bore most of their weight on their hocks with their feet only lightly touching the ground (L. Dixon, Pers. Obs.) which decreased foot contact with litter This combination of lower stocking density, improved litter quality and reduced foot pad contact with the litter may explain our results compared to studies using commercial faster growing broiler management guidelines as lower litter pH (higher acidity) is linked to higher stocking densities and worse litter quality which leads to higher FPD scores [33]. An additional factor which may help explain the differences in our results compared to other studies is that a number of published experiments which assess broilers are older -10 years or more–(e.g. [18]) and genetics of the birds will have changed in that time period and potential improvements on the factors assessed may have been made.

Slower growing S birds spent less time feeding, drinking and sitting, and more time active (foraging, locomotion, preening, perching, dustbathing) than the other breeds. This is similar to other studies which also found that slower growing broilers walked, foraged and perched more, and fed, drank and sat less than faster growing birds [34–36]. The difference between the S and the other breeds in perching was quite pronounced—a maximum of 13% of the light hours was spent perching in the S birds versus a maximum of 3% for the faster growing breeds. All pens had a 1.3m perch per 50 birds. After about the second week of life, the perches in the S pens were almost always occupied and if more perch space had been provided, most likely more birds would have made use of it. We know that laying hens are highly motivated to perch (e.g. [37]) and when sufficient perch space is provided, slower growing broilers show similar levels of perching to laying hens [36]. Therefore, it would appear that the slower growing broilers also share a motivation to perch. The faster growing broilers continued to try to perch and some were successful even up until the end of the trials. However, anecdotally, the faster growing birds would often have trouble balancing on the perches and would have to step off or risk falling. Therefore it is plausible that the faster growing birds are still motivated to perch but are physically unable to do so easily and the inability to satisfy this motivation could cause frustration and stress [38]. There have been studies comparing the use of different perch types in broilers and it seems that ones with mesh between the perches or platforms would be more easily utilized by faster growing breeds [39–40]. Preening was very similar between faster and slower growing birds up until day 37 when the S birds preened significantly more. It has been postulated that faster growing broilers become frustrated with their inability to perform active behaviours as they age, leading to an increase in displacement preening behaviour [36]. Displacement preening has been found in laying hens when they were thwarted from performing feeding behaviour under experimental conditions as a frustration behaviour along with stereotyped pacing and escape movements [41]. Displacement preening could potentially account for the preening levels of the faster growing birds; however the increase in preening in the slower growing S breed would seem unlikely to be due to the same frustration and further research would be needed to confirm this hypothesis. The differences in behaviour between the faster and slower growers became larger as the birds aged and this has been found in a number of other studies as well. For example, sitting in faster growing birds was found to increase from 75% of the time in the first week of age to 90% at five weeks of age while foraging declined over this time period (Bessei 1992a in [3]).

As expected, S birds consumed more feed, had a lower ADG and higher FCR than the faster growing breeds which is also consistent with past research (e.g. [25, 42]). Additionally, S birds also had lighter breast fillet weights and heavier leg weights after processing which also matches past research [25]. S birds were found to have lower levels of breast striations (white striping) and wooden breast than the other breeds. Kuttappan et al [29] found levels of moderate to severe breast striations in 41–72% of breast fillets in faster growing birds which is similar to the 63–78% found here. Breast striations and wooden breast are considered to be muscle abnormalities or myopathies that are associated with the increase in growth and muscle mass of faster growing broilers and which can result in downgraded carcasses [43]. The white breast striations are also negatively perceived by consumers which could influence their choice to purchase whole chickens or breast fillets [44].

Based on all of this evidence, it is clear that the welfare and behaviour of a slower growing breed is improved in terms of the birds being more active which contributes to lower gait, hock and breast cleanliness scores compared to that of faster growing breeds. Faster growing breeds are more efficient at converting feed to body weight and are slaughtered at a younger age than slower growers. Some of the faster growing breeds showed lower scores in the welfare assessments over the others in, e.g. gait score or feather score, but the improvements were not consistent across all measured factors and these breeds were still far from the slower growing breed in most measures. Also there are other welfare, behaviour and meat quality traits that could be assessed, such as conducting welfare assessments when the birds were younger, motivation testing or drip loss, and these may or may not show differences between the faster and slower growing birds.

When considering the mortality rates (especially if all the gait score 3 and above birds were culled as is often recommended, e.g. [45]) plus the reduction in meat downgrades, the difference in production costs may not be that large. It has been quoted (albeit in a popular press article) that when comparing production in faster and slower growing broilers within the EU that ‘the gross margin per square meter per day and the income of the farm which switched to slower growing stays intact. The extra cost are compensated by a higher retail price for breast meat (+1.50 euros/kg) and better valuation of whole birds and cut ups’ [46]. However consumers also need to be willing to purchase higher priced chicken but they tend not to know much about how chicken is produced. In a recent study, Lusk [47] found that only 3% of chicken consumers surveyed in the USA knew broilers were housed in cage-free systems and only 12% had any knowledge of the availability of slower growing breeds. An in-depth analyses of the cost to produce slower growing chicken versus the increase in price paid to the producer for a premium product may help clarify the difference (if any) in production costs between the two systems. Breast meat from slower growing birds was also found to have higher protein and lower fat levels than in faster growers [48] which could encourage increased consumer purchasing. However, one issue with the use of slower growing breeds is that production of faster growing broiler breeds is more environmentally sustainable than production of slower growing breeds. As these broilers eat less feed and take less time to reach slaughter weight, they have a reduced environmental impact compared to slower growing alternatives [49]. For example, faster growing broiler production was calculated to have a greenhouse gas emission of about 5 CO_2_-equivalent kg per bird while the slower growing birds were calculated to have emissions just over 6 CO_2_-equivalent kg per bird [50]. However this analyses did not take into account losses from mortality and meat quality downgrades which are larger in faster than slower growing broiler production and would increase environmental burdens so it is not clear how big the difference actually is on a flock basis. Sustainable agriculture studies do not tend to give animal welfare measures a weighting or cost in their analyses and while with the increasing human population, sustainability of our food sources is important, considering animal welfare along with these other measures could help find production methods that will improve both. Furthermore, an increase in public education about the housing, management, behaviour and welfare of broiler chickens may increase the demand for higher welfare meat which may encourage more producers to enter this market and more sustainable solutions within slower growing production to be investigated.

## Conclusion

In conclusion, in this experimental study the slower growing S birds had improved welfare and behaviour measures such as increased activity and lower gait, hock and breast cleanliness scores as well as lower mortality rates compared to the faster growing birds. Slower growth also had advantages for meat quality measures; however they are still at a disadvantage to faster growing breeds in terms of most production measures. Birds in this study were managed according to high standards and different standards may be applied on commercial farms or be required by different countries which could affect the magnitude of the results. An EU Commission report identified broiler production systems as one of the top three animal production systems that needed the most improvement in terms of animal welfare and protection [51] and while it is encouraging to see breeding companies being active in including some welfare measures into their breeding schemes, faster growth rates still appear to be a large factor in worse welfare assessments compared to a slower growing alternative although large scale research at different commercial facilities is needed to fully quantify welfare measures under different types of commercial management systems.

## Supporting information

S1 Data (PDF)

S2 Data (PDF)

S3 Data (PDF)

S4 Data (PDF)

S5 Data (PDF)

S6 Data (PDF)

S7 Data (PDF)

S8 Data (PDF)

S9 Data (PDF)

S10 Data (PDF)

S11 Data (PDF)

S12 Data (PDF)

S13 Data (PDF)

S14 Data (PDF)

S15 Data (PDF)
