# Supplementary material for: Slow and steady wins the race: The behaviour and welfare of commercial faster growing broiler breeds compared to a commercial slower growing breed
Source: PLoS One. 2020 Apr 6;15(4):e0231006. doi: 10.1371/journal.pone.0231006 (PMC7135253; doi:10.1371/journal.pone.0231006)
Supplement: S1 Data — (PDF) [file pone.0231006.s001.pdf]

Replicate	Pen	Breed	Age (d)	Avg indiv weight (g)
1	3	FC	0	38.6
1	7	FC	0	38.7
1	12	FC	0	39.0
1	16	FC	0	38.7
1	2	FA	0	41.2
1	6	FA	0	40.5
1	11	FA	0	41.0
1	15	FA	0	40.7
1	1	FB	0	37.1
1	5	FB	0	37.1
1	10	FB	0	37.2
1	14	FB	0	36.8
1	4	S	0	35.7
1	8	S	0	35.0
1	9	S	0	35.8
1	13	S	0	35.9
1	3	FC	14	355.0
1	7	FC	14	348.9
1	12	FC	14	364.3
1	16	FC	14	382.3
1	2	FA	14	415.3
1	6	FA	14	401.0
1	11	FA	14	394.0
1	15	FA	14	399.0
1	1	FB	14	367.9
1	5	FB	14	370.8
1	10	FB	14	358.8
1	14	FB	14	344.9
1	4	S	14	257.3
1	8	S	14	249.0
1	9	S	14	280.6
1	13	S	14	276.0
1	3	FC	28	1435.7
1	7	FC	28	1403.3
1	12	FC	28	1471.4
1	16	FC	28	1434.4
1	2	FA	28	1591.7
1	6	FA	28	1586.5
1	11	FA	28	1536.7
1	15	FA	28	1575.0
1	1	FB	28	1407.0
1	5	FB	28	1478.6
1	10	FB	28	1450.0
1	14	FB	28	1393.0
1	4	S	28	871.3
1	8	S	28	874.0
1	9	S	28	945.3
1	13	S	28	930.4
1	3	FC	35	2240.8
1	7	FC	35	2157.8
1	12	FC	35	2248.0
1	16	FC	35	2227.1

1	2	FA	35	2290.5
1	6	FA	35	2281.2
1	11	FA	35	2236.9
1	15	FA	35	2216.5
1	1	FB	35	2125.0
1	5	FB	35	2237.8
1	10	FB	35	2181.6
1	14	FB	35	2132.0
1	4	S	35	1324.0
1	8	S	35	1329.6
1	9	S	35	1393.9
1	13	S	35	1386.7
1	3	FC	42	2747.9
1	7	FC	42	2705.7
1	12	FC	42	2833.0
1	16	FC	42	2734.4
1	2	FA	42	2856.4
1	6	FA	42	2854.4
1	11	FA	42	2587.0
1	15	FA	42	2851.1
1	1	FB	42	2601.0
1	5	FB	42	2719.8
1	10	FB	42	2710.2
1	14	FB	42	2875.0
1	4	S	42	1723.0
1	8	S	42	1728.6
1	9	S	42	1821.4
1	13	S	42	1812.2
2	1	FA	0	44.0
2	2	FB	0	40.0
2	3	S	0	42.0
2	4	FC	0	37.0
2	5	FA	0	44.0
2	6	FB	0	40.0
2	7	S	0	42.0
2	8	FC	0	36.0
2	9	FB	0	41.0
2	10	S	0	42.0
2	11	FC	0	36.0
2	12	FA	0	44.0
2	13	FB	0	40.0
2	14	S	0	41.0
2	15	FC	0	36.0
2	16	FA	0	42.0
2	1	FA	14	397.2
2	2	FB	14	356.0
2	3	S	14	281.8
2	4	FC	14	327.8
2	5	FA	14	417.0
2	6	FB	14	353.2
2	7	S	14	272.9
2	8	FC	14	353.2
2	9	FB	14	375.5

2	10	S	14	279.5
2	11	FC	14	319.1
2	12	FA	14	420.5
2	13	FB	14	367.0
2	14	S	14	271.4
2	15	FC	14	353.1
2	16	FA	14	452.0
2	1	FA	28	1545.1
2	2	FB	28	1425.5
2	3	S	28	984.9
2	4	FC	28	1419.0
2	5	FA	28	1700.0
2	6	FB	28	1511.7
2	7	S	28	967.3
2	8	FC	28	1427.0
2	9	FB	28	1492.6
2	10	S	28	982.4
2	11	FC	28	1319.8
2	12	FA	28	1633.0
2	13	FB	28	1469.6
2	14	S	28	960.0
2	15	FC	28	1408.0
2	16	FA	28	1713.7
2	1	FA	35	2352.0
2	2	FB	35	2220.2
2	3	S	35	1451.0
2	4	FC	35	2260.9
2	5	FA	35	2488.5
2	6	FB	35	2310.9
2	7	S	35	1415.0
2	8	FC	35	2258.1
2	9	FB	35	2264.5
2	10	S	35	1434.3
2	11	FC	35	2094.9
2	12	FA	35	2392.7
2	13	FB	35	2202.2
2	14	S	35	1361.0
2	15	FC	35	2139.7
2	16	FA	35	2545.9
2	1	FA	42	3187.5
2	2	FB	42	2997.0
2	3	S	42	1956.9
2	4	FC	42	3076.0
2	5	FA	42	3488.5
2	6	FB	42	3110.0
2	7	S	42	1917.6
2	8	FC	42	3087.0
2	9	FB	42	3064.8
2	10	S	42	1920.4
2	11	FC	42	2904.0
2	12	FA	42	3025.0
2	13	FB	42	2951.1
2	14	S	42	1938.8

2	15	FC	42	2996.0
2	16	FA	42	3375.5
