# Supplementary material for: Slow and steady wins the race: The behaviour and welfare of commercial faster growing broiler breeds compared to a commercial slower growing breed
Source: PLoS One. 2020 Apr 6;15(4):e0231006. doi: 10.1371/journal.pone.0231006 (PMC7135253; doi:10.1371/journal.pone.0231006)
Supplement: S2 Data — (PDF) [file pone.0231006.s002.pdf]

Cause of mortality	Proportion
Yolk Sac	0.32
Flip Over	0.09
Lame	0.24
Injured	0.05
Unresponsive	0.13
Runt	0.05
Unknown Cause	0.12
