# Supplementary material for: Slow and steady wins the race: The behaviour and welfare of commercial faster growing broiler breeds compared to a commercial slower growing breed
Source: PLoS One. 2020 Apr 6;15(4):e0231006. doi: 10.1371/journal.pone.0231006 (PMC7135253; doi:10.1371/journal.pone.0231006)
Supplement: S3 Data — (PDF) [file pone.0231006.s003.pdf]

| Replicate | Breed | Pen | Prop Total | Prop cull - lame |
|---|---|---|---|---|
| 1 | FB | 1 | 0.055 | 0.000 |
| 1 | FA | 2 | 0.140 | 0.040 |
| 1 | FC | 3 | 0.100 | 0.000 |
| 1 | S | 4 | 0.018 | 0.000 |
| 1 | FB | 5 | 0.109 | 0.073 |
| 1 | FA | 6 | 0.140 | 0.040 |
| 1 | FC | 7 | 0.140 | 0.020 |
| 1 | S | 8 | 0.109 | 0.000 |
| 1 | S | 9 | 0.036 | 0.000 |
| 1 | FB | 10 | 0.077 | 0.000 |
| 1 | FA | 11 | 0.091 | 0.018 |
| 1 | FC | 12 | 0.073 | 0.000 |
| 1 | S | 13 | 0.056 | 0.019 |
| 1 | FB | 14 | 0.135 | 0.019 |
| 1 | FA | 15 | 0.145 | 0.036 |
| 1 | FC | 16 | 0.091 | 0.000 |
| 2 | FA | 1 | 0.13 | 0.07 |
| 2 | FB | 2 | 0.16 | 0.04 |
| 2 | S | 3 | 0.02 | 0.00 |
| 2 | FC | 4 | 0.02 | 0.00 |
| 2 | FA | 5 | 0.08 | 0.04 |
| 2 | FB | 6 | 0.06 | 0.00 |
| 2 | S | 7 | 0.08 | 0.04 |
| 2 | FC | 8 | 0.08 | 0.00 |
| 2 | FB | 9 | 0.20 | 0.04 |
| 2 | S | 10 | 0.04 | 0.00 |
| 2 | FC | 11 | 0.05 | 0.00 |
| 2 | FA | 12 | 0.05 | 0.04 |
| 2 | FB | 13 | 0.10 | 0.06 |
| 2 | S | 14 | 0.06 | 0.02 |
| 2 | FC | 15 | 0.04 | 0.00 |
| 2 | FA | 16 | 0.08 | 0.04 |
