# Supplementary material for: Slow and steady wins the race: The behaviour and welfare of commercial faster growing broiler breeds compared to a commercial slower growing breed
Source: PLoS One. 2020 Apr 6;15(4):e0231006. doi: 10.1371/journal.pone.0231006 (PMC7135253; doi:10.1371/journal.pone.0231006)
Supplement: S4 Data — (PDF) [file pone.0231006.s004.pdf]

Replicate	Pen	Breed	WA	Sex	Weight (g)
1	1	FB	1	F	2050
1	1	FB	1	M	2465
1	1	FB	2	F	2375
1	1	FB	2	M	2801
1	10	FB	1	F	2122
1	10	FB	1	M	2464
1	10	FB	2	F	2422
1	10	FB	2	M	2830
1	11	FA	1	F	2058
1	11	FA	1	M	2402
1	11	FA	2	F	2299
1	11	FA	2	M	2695
1	12	FC	1	F	2044
1	12	FC	1	M	2457
1	12	FC	2	F	2374
1	12	FC	2	M	2817
1	13	S	1	F	2059
1	13	S	1	M	2473
1	13	S	2	F	2264
1	13	S	2	M	2853
1	14	FB	1	F	2090
1	14	FB	1	M	2381
1	14	FB	2	F	2391
1	14	FB	2	M	2755
1	15	FA	1	F	1976
1	15	FA	1	M	2427
1	15	FA	2	F	2279
1	15	FA	2	M	2779
1	16	FC	1	F	2077
1	16	FC	1	M	2458
1	16	FC	2	F	2410
1	16	FC	2	M	2790
1	2	FA	1	F	2135
1	2	FA	1	M	2508
1	2	FA	2	F	2368
1	2	FA	2	M	2855
1	3	FC	1	F	2067
1	3	FC	1	M	2458
1	3	FC	2	F	2323
1	3	FC	2	M	2733
1	4	S	1	F	2072
1	4	S	1	M	2337
1	4	S	2	F	2322
1	4	S	2	M	2731
1	5	FB	1	F	2092
1	5	FB	1	M	2383
1	5	FB	2	F	2325
1	5	FB	2	M	2763
1	6	FA	1	F	2118
1	6	FA	1	M	2551
1	6	FA	2	F	2381
1	6	FA	2	M	2860

1	7	FC	1	F	2079
1	7	FC	1	M	2453
1	7	FC	2	F	2293
1	7	FC	2	M	2773
1	8	S	1	F	2016
1	8	S	1	M	2467
1	8	S	2	F	2317
1	8	S	2	M	2862
1	9	S	1	F	2056
1	9	S	1	M	2461
1	9	S	2	F	2281
1	9	S	2	M	2835
2	1	FA	1	F	2050
2	1	FA	1	M	2325
2	1	FA	2	F	2302
2	1	FA	2	M	2692
2	10	S	1	F	2017
2	10	S	1	M	2338
2	10	S	2	F	2296
2	10	S	2	M	2802
2	11	FC	1	F	2081
2	11	FC	1	M	2285
2	11	FC	2	F	2290
2	11	FC	2	M	2621
2	12	FA	1	F	2095
2	12	FA	1	M	2320
2	12	FA	2	F	2401
2	12	FA	2	M	2669
2	13	FB	1	F	2097
2	13	FB	1	M	2293
2	13	FB	2	F	2294
2	13	FB	2	M	2604
2	14	S	1	F	2038
2	14	S	1	M	2270
2	14	S	2	F	2269
2	14	S	2	M	2696
2	15	FC	1	F	2060
2	15	FC	1	M	2307
2	15	FC	2	F	2294
2	15	FC	2	M	2715
2	16	FA	1	F	2104
2	16	FA	1	M	2364
2	16	FA	2	F	2481
2	16	FA	2	M	2840
2	2	FB	1	F	2059
2	2	FB	1	M	2278
2	2	FB	2	F	2327
2	2	FB	2	M	2619
2	3	S	1	F	2025
2	3	S	1	M	2398
2	3	S	2	F	2283
2	3	S	2	M	2820
2	4	FC	1	F	2046

2	4	FC	1	M	2403
2	4	FC	2	F	2335
2	4	FC	2	M	2817
2	5	FA	1	F	2053
2	5	FA	1	M	2402
2	5	FA	2	F	2359
2	5	FA	2	M	2824
2	6	FB	1	F	2131
2	6	FB	1	M	2317
2	6	FB	2	F	2418
2	6	FB	2	M	2664
2	7	S	1	F	2037
2	7	S	1	M	2405
2	7	S	2	F	2339
2	7	S	2	M	2822
2	8	FC	1	F	2127
2	8	FC	1	M	2397
2	8	FC	2	F	2399
2	8	FC	2	M	2767
2	9	FB	1	F	2180
2	9	FB	1	M	2319
2	9	FB	2	F	2396
2	9	FB	2	M	2639
