# Supplementary material for: Slow and steady wins the race: The behaviour and welfare of commercial faster growing broiler breeds compared to a commercial slower growing breed
Source: PLoS One. 2020 Apr 6;15(4):e0231006. doi: 10.1371/journal.pone.0231006 (PMC7135253; doi:10.1371/journal.pone.0231006)
Supplement: S5 Data — (PDF) [file pone.0231006.s005.pdf]

Replicate	Pen	Breed	Sex	WA	GS	Prop
1	1	FB	F	1	0	0.00
1	1	FB	M	1	0	0.00
1	1	FB	F	2	0	0.00
1	1	FB	M	2	0	0.00
1	2	FA	F	1	0	0.00
1	2	FA	M	1	0	0.00
1	2	FA	F	2	0	0.00
1	2	FA	M	2	0	0.00
1	3	FC	F	1	0	0.00
1	3	FC	M	1	0	0.00
1	3	FC	F	2	0	0.00
1	3	FC	M	2	0	0.00
1	4	S	F	1	0	0.36
1	4	S	M	1	0	0.13
1	4	S	F	2	0	0.00
1	4	S	M	2	0	0.00
1	5	FB	F	1	0	0.00
1	5	FB	M	1	0	0.00
1	5	FB	F	2	0	0.00
1	5	FB	M	2	0	0.00
1	6	FA	F	1	0	0.00
1	6	FA	M	1	0	0.00
1	6	FA	F	2	0	0.00
1	6	FA	M	2	0	0.00
1	7	FC	F	1	0	0.00
1	7	FC	M	1	0	0.00
1	7	FC	F	2	0	0.00
1	7	FC	M	2	0	0.00
1	8	S	F	1	0	0.36
1	8	S	M	1	0	0.00
1	8	S	F	2	0	0.00
1	8	S	M	2	0	0.00
1	9	S	F	1	0	0.05
1	9	S	M	1	0	0.00
1	9	S	F	2	0	0.00
1	9	S	M	2	0	0.00
1	10	FB	F	1	0	0.00
1	10	FB	M	1	0	0.00
1	10	FB	F	2	0	0.00
1	10	FB	M	2	0	0.00
1	11	FA	F	1	0	0.00
1	11	FA	M	1	0	0.00
1	11	FA	F	2	0	0.00
1	11	FA	M	2	0	0.00
1	12	FC	F	1	0	0.00
1	12	FC	M	1	0	0.00
1	12	FC	F	2	0	0.00
1	12	FC	M	2	0	0.00
1	13	S	F	1	0	0.10
1	13	S	M	1	0	0.00
1	13	S	F	2	0	0.00
1	13	S	M	2	0	0.00

1	14	FB	F	1	0	0.00
1	14	FB	M	1	0	0.00
1	14	FB	F	2	0	0.00
1	14	FB	M	2	0	0.00
1	15	FA	F	1	0	0.00
1	15	FA	M	1	0	0.00
1	15	FA	F	2	0	0.00
1	15	FA	M	2	0	0.00
1	16	FC	F	1	0	0.00
1	16	FC	M	1	0	0.00
1	16	FC	F	2	0	0.00
1	16	FC	M	2	0	0.00
2	1	FA	F	1	0	0.00
2	1	FA	M	1	0	0.00
2	1	FA	F	2	0	0.00
2	1	FA	M	2	0	0.00
2	2	FB	F	1	0	0.00
2	2	FB	M	1	0	0.00
2	2	FB	F	2	0	0.00
2	2	FB	M	2	0	0.00
2	3	S	F	1	0	0.33
2	3	S	M	1	0	0.04
2	3	S	F	2	0	0.30
2	3	S	M	2	0	0.00
2	4	FC	F	1	0	0.00
2	4	FC	M	1	0	0.00
2	4	FC	F	2	0	0.00
2	4	FC	M	2	0	0.00
2	5	FA	F	1	0	0.00
2	5	FA	M	1	0	0.00
2	5	FA	F	2	0	0.00
2	5	FA	M	2	0	0.00
2	6	FB	F	1	0	0.00
2	6	FB	M	1	0	0.00
2	6	FB	F	2	0	0.00
2	6	FB	M	2	0	0.00
2	7	S	F	1	0	0.40
2	7	S	M	1	0	0.00
2	7	S	F	2	0	0.16
2	7	S	M	2	0	0.00
2	8	FC	F	1	0	0.00
2	8	FC	M	1	0	0.00
2	8	FC	F	2	0	0.00
2	8	FC	M	2	0	0.00
2	9	FB	F	1	0	0.00
2	9	FB	M	1	0	0.00
2	9	FB	F	2	0	0.00
2	9	FB	M	2	0	0.00
2	10	S	F	1	0	0.62
2	10	S	M	1	0	0.10
2	10	S	F	2	0	0.38
2	10	S	M	2	0	0.00
2	11	FC	F	1	0	0.00

2	11	FC	M	1	0	0.00
2	11	FC	F	2	0	0.00
2	11	FC	M	2	0	0.00
2	12	FA	F	1	0	0.00
2	12	FA	M	1	0	0.00
2	12	FA	F	2	0	0.00
2	12	FA	M	2	0	0.00
2	13	FB	F	1	0	0.00
2	13	FB	M	1	0	0.00
2	13	FB	F	2	0	0.00
2	13	FB	M	2	0	0.00
2	14	S	F	1	0	0.40
2	14	S	M	1	0	0.07
2	14	S	F	2	0	0.32
2	14	S	M	2	0	0.03
2	15	FC	F	1	0	0.00
2	15	FC	M	1	0	0.00
2	15	FC	F	2	0	0.00
2	15	FC	M	2	0	0.00
2	16	FA	F	1	0	0.00
2	16	FA	M	1	0	0.00
2	16	FA	F	2	0	0.00
2	16	FA	M	2	0	0.00
1	1	FB	F	1	1	0.27
1	1	FB	M	1	1	0.00
1	1	FB	F	2	1	0.13
1	1	FB	M	2	1	0.00
1	2	FA	F	1	1	0.14
1	2	FA	M	1	1	0.00
1	2	FA	F	2	1	0.07
1	2	FA	M	2	1	0.00
1	3	FC	F	1	1	0.23
1	3	FC	M	1	1	0.04
1	3	FC	F	2	1	0.18
1	3	FC	M	2	1	0.04
1	4	S	F	1	1	0.56
1	4	S	M	1	1	0.42
1	4	S	F	2	1	0.24
1	4	S	M	2	1	0.04
1	5	FB	F	1	1	0.14
1	5	FB	M	1	1	0.00
1	5	FB	F	2	1	0.38
1	5	FB	M	2	1	0.00
1	6	FA	F	1	1	0.14
1	6	FA	M	1	1	0.06
1	6	FA	F	2	1	0.04
1	6	FA	M	2	1	0.00
1	7	FC	F	1	1	0.20
1	7	FC	M	1	1	0.05
1	7	FC	F	2	1	0.32
1	7	FC	M	2	1	0.00
1	8	S	F	1	1	0.54
1	8	S	M	1	1	0.43

1	8	S	F	2	1	0.43
1	8	S	M	2	1	0.00
1	9	S	F	1	1	0.40
1	9	S	M	1	1	0.10
1	9	S	F	2	1	0.45
1	9	S	M	2	1	0.00
1	10	FB	F	1	1	0.04
1	10	FB	M	1	1	0.00
1	10	FB	F	2	1	0.14
1	10	FB	M	2	1	0.00
1	11	FA	F	1	1	0.26
1	11	FA	M	1	1	0.08
1	11	FA	F	2	1	0.04
1	11	FA	M	2	1	0.00
1	12	FC	F	1	1	0.17
1	12	FC	M	1	1	0.00
1	12	FC	F	2	1	0.09
1	12	FC	M	2	1	0.04
1	13	S	F	1	1	0.62
1	13	S	M	1	1	0.18
1	13	S	F	2	1	0.29
1	13	S	M	2	1	0.00
1	14	FB	F	1	1	0.17
1	14	FB	M	1	1	0.00
1	14	FB	F	2	1	0.00
1	14	FB	M	2	1	0.00
1	15	FA	F	1	1	0.10
1	15	FA	M	1	1	0.04
1	15	FA	F	2	1	0.00
1	15	FA	M	2	1	0.04
1	16	FC	F	1	1	0.08
1	16	FC	M	1	1	0.09
1	16	FC	F	2	1	0.08
1	16	FC	M	2	1	0.09
2	1	FA	F	1	1	0.29
2	1	FA	M	1	1	0.05
2	1	FA	F	2	1	0.00
2	1	FA	M	2	1	0.00
2	2	FB	F	1	1	0.29
2	2	FB	M	1	1	0.04
2	2	FB	F	2	1	0.09
2	2	FB	M	2	1	0.00
2	3	S	F	1	1	0.67
2	3	S	M	1	1	0.50
2	3	S	F	2	1	0.57
2	3	S	M	2	1	0.33
2	4	FC	F	1	1	0.30
2	4	FC	M	1	1	0.00
2	4	FC	F	2	1	0.14
2	4	FC	M	2	1	0.00
2	5	FA	F	1	1	0.29
2	5	FA	M	1	1	0.08
2	5	FA	F	2	1	0.05

2	5	FA	M	2	1	0.00
2	6	FB	F	1	1	0.28
2	6	FB	M	1	1	0.07
2	6	FB	F	2	1	0.11
2	6	FB	M	2	1	0.04
2	7	S	F	1	1	0.56
2	7	S	M	1	1	0.52
2	7	S	F	2	1	0.72
2	7	S	M	2	1	0.21
2	8	FC	F	1	1	0.14
2	8	FC	M	1	1	0.00
2	8	FC	F	2	1	0.12
2	8	FC	M	2	1	0.04
2	9	FB	F	1	1	0.35
2	9	FB	M	1	1	0.18
2	9	FB	F	2	1	0.05
2	9	FB	M	2	1	0.04
2	10	S	F	1	1	0.33
2	10	S	M	1	1	0.41
2	10	S	F	2	1	0.48
2	10	S	M	2	1	0.44
2	11	FC	F	1	1	0.29
2	11	FC	M	1	1	0.05
2	11	FC	F	2	1	0.11
2	11	FC	M	2	1	0.04
2	12	FA	F	1	1	0.33
2	12	FA	M	1	1	0.10
2	12	FA	F	2	1	0.07
2	12	FA	M	2	1	0.00
2	13	FB	F	1	1	0.22
2	13	FB	M	1	1	0.16
2	13	FB	F	2	1	0.07
2	13	FB	M	2	1	0.06
2	14	S	F	1	1	0.55
2	14	S	M	1	1	0.62
2	14	S	F	2	1	0.63
2	14	S	M	2	1	0.53
2	15	FC	F	1	1	0.40
2	15	FC	M	1	1	0.24
2	15	FC	F	2	1	0.19
2	15	FC	M	2	1	0.07
2	16	FA	F	1	1	0.26
2	16	FA	M	1	1	0.00
2	16	FA	F	2	1	0.00
2	16	FA	M	2	1	0.00
1	1	FB	F	1	2	0.73
1	1	FB	M	1	2	0.70
1	1	FB	F	2	2	0.70
1	1	FB	M	2	2	0.15
1	2	FA	F	1	2	0.64
1	2	FA	M	1	2	0.63
1	2	FA	F	2	2	0.57
1	2	FA	M	2	2	0.00

1	3	FC	F	1	2	0.73
1	3	FC	M	1	2	0.56
1	3	FC	F	2	2	0.50
1	3	FC	M	2	2	0.19
1	4	S	F	1	2	0.08
1	4	S	M	1	2	0.46
1	4	S	F	2	2	0.72
1	4	S	M	2	2	0.54
1	5	FB	F	1	2	0.86
1	5	FB	M	1	2	0.71
1	5	FB	F	2	2	0.52
1	5	FB	M	2	2	0.48
1	6	FA	F	1	2	0.71
1	6	FA	M	1	2	0.29
1	6	FA	F	2	2	0.75
1	6	FA	M	2	2	0.12
1	7	FC	F	1	2	0.76
1	7	FC	M	1	2	0.63
1	7	FC	F	2	2	0.56
1	7	FC	M	2	2	0.32
1	8	S	F	1	2	0.11
1	8	S	M	1	2	0.57
1	8	S	F	2	2	0.54
1	8	S	M	2	2	0.38
1	9	S	F	1	2	0.55
1	9	S	M	1	2	0.59
1	9	S	F	2	2	0.55
1	9	S	M	2	2	0.34
1	10	FB	F	1	2	0.82
1	10	FB	M	1	2	0.43
1	10	FB	F	2	2	0.71
1	10	FB	M	2	2	0.19
1	11	FA	F	1	2	0.57
1	11	FA	M	1	2	0.44
1	11	FA	F	2	2	0.74
1	11	FA	M	2	2	0.39
1	12	FC	F	1	2	0.78
1	12	FC	M	1	2	0.73
1	12	FC	F	2	2	0.83
1	12	FC	M	2	2	0.40
1	13	S	F	1	2	0.29
1	13	S	M	1	2	0.57
1	13	S	F	2	2	0.71
1	13	S	M	2	2	0.29
1	14	FB	F	1	2	0.74
1	14	FB	M	1	2	0.56
1	14	FB	F	2	2	0.82
1	14	FB	M	2	2	0.31
1	15	FA	F	1	2	0.86
1	15	FA	M	1	2	0.46
1	15	FA	F	2	2	0.95
1	15	FA	M	2	2	0.13
1	16	FC	F	1	2	0.81

1	16	FC	M	1	2	0.73
1	16	FC	F	2	2	0.88
1	16	FC	M	2	2	0.23
2	1	FA	F	1	2	0.64
2	1	FA	M	1	2	0.50
2	1	FA	F	2	2	0.81
2	1	FA	M	2	2	0.09
2	2	FB	F	1	2	0.71
2	2	FB	M	1	2	0.63
2	2	FB	F	2	2	0.91
2	2	FB	M	2	2	0.56
2	3	S	F	1	2	0.00
2	3	S	M	1	2	0.42
2	3	S	F	2	2	0.13
2	3	S	M	2	2	0.59
2	4	FC	F	1	2	0.70
2	4	FC	M	1	2	0.70
2	4	FC	F	2	2	0.82
2	4	FC	M	2	2	0.32
2	5	FA	F	1	2	0.63
2	5	FA	M	1	2	0.46
2	5	FA	F	2	2	0.86
2	5	FA	M	2	2	0.15
2	6	FB	F	1	2	0.67
2	6	FB	M	1	2	0.68
2	6	FB	F	2	2	0.78
2	6	FB	M	2	2	0.36
2	7	S	F	1	2	0.04
2	7	S	M	1	2	0.44
2	7	S	F	2	2	0.12
2	7	S	M	2	2	0.67
2	8	FC	F	1	2	0.83
2	8	FC	M	1	2	0.57
2	8	FC	F	2	2	0.72
2	8	FC	M	2	2	0.40
2	9	FB	F	1	2	0.61
2	9	FB	M	1	2	0.64
2	9	FB	F	2	2	0.81
2	9	FB	M	2	2	0.39
2	10	S	F	1	2	0.05
2	10	S	M	1	2	0.48
2	10	S	F	2	2	0.14
2	10	S	M	2	2	0.52
2	11	FC	F	1	2	0.71
2	11	FC	M	1	2	0.77
2	11	FC	F	2	2	0.85
2	11	FC	M	2	2	0.48
2	12	FA	F	1	2	0.53
2	12	FA	M	1	2	0.55
2	12	FA	F	2	2	0.89
2	12	FA	M	2	2	0.30
2	13	FB	F	1	2	0.74
2	13	FB	M	1	2	0.58

2	13	FB	F	2	2	0.78
2	13	FB	M	2	2	0.33
2	14	S	F	1	2	0.05
2	14	S	M	1	2	0.31
2	14	S	F	2	2	0.05
2	14	S	M	2	2	0.40
2	15	FC	F	1	2	0.56
2	15	FC	M	1	2	0.60
2	15	FC	F	2	2	0.81
2	15	FC	M	2	2	0.45
2	16	FA	F	1	2	0.70
2	16	FA	M	1	2	0.78
2	16	FA	F	2	2	0.82
2	16	FA	M	2	2	0.33
1	1	FB	F	1	3	0.00
1	1	FB	M	1	3	0.30
1	1	FB	F	2	3	0.17
1	1	FB	M	2	3	0.85
1	2	FA	F	1	3	0.14
1	2	FA	M	1	3	0.37
1	2	FA	F	2	3	0.36
1	2	FA	M	2	3	1.00
1	3	FC	F	1	3	0.05
1	3	FC	M	1	3	0.41
1	3	FC	F	2	3	0.32
1	3	FC	M	2	3	0.77
1	4	S	F	1	3	0.00
1	4	S	M	1	3	0.00
1	4	S	F	2	3	0.04
1	4	S	M	2	3	0.42
1	5	FB	F	1	3	0.00
1	5	FB	M	1	3	0.29
1	5	FB	F	2	3	0.10
1	5	FB	M	2	3	0.52
1	6	FA	F	1	3	0.14
1	6	FA	M	1	3	0.65
1	6	FA	F	2	3	0.21
1	6	FA	M	2	3	0.88
1	7	FC	F	1	3	0.04
1	7	FC	M	1	3	0.32
1	7	FC	F	2	3	0.12
1	7	FC	M	2	3	0.68
1	8	S	F	1	3	0.00
1	8	S	M	1	3	0.00
1	8	S	F	2	3	0.04
1	8	S	M	2	3	0.62
1	9	S	F	1	3	0.00
1	9	S	M	1	3	0.31
1	9	S	F	2	3	0.00
1	9	S	M	2	3	0.66
1	10	FB	F	1	3	0.14
1	10	FB	M	1	3	0.57
1	10	FB	F	2	3	0.14

1	10	FB	M	2	3	0.81
1	11	FA	F	1	3	0.13
1	11	FA	M	1	3	0.44
1	11	FA	F	2	3	0.22
1	11	FA	M	2	3	0.61
1	12	FC	F	1	3	0.04
1	12	FC	M	1	3	0.27
1	12	FC	F	2	3	0.09
1	12	FC	M	2	3	0.56
1	13	S	F	1	3	0.00
1	13	S	M	1	3	0.25
1	13	S	F	2	3	0.00
1	13	S	M	2	3	0.71
1	14	FB	F	1	3	0.00
1	14	FB	M	1	3	0.44
1	14	FB	F	2	3	0.18
1	14	FB	M	2	3	0.69
1	15	FA	F	1	3	0.05
1	15	FA	M	1	3	0.50
1	15	FA	F	2	3	0.05
1	15	FA	M	2	3	0.83
1	16	FC	F	1	3	0.12
1	16	FC	M	1	3	0.18
1	16	FC	F	2	3	0.04
1	16	FC	M	2	3	0.68
2	1	FA	F	1	3	0.07
2	1	FA	M	1	3	0.41
2	1	FA	F	2	3	0.19
2	1	FA	M	2	3	0.86
2	2	FB	F	1	3	0.00
2	2	FB	M	1	3	0.33
2	2	FB	F	2	3	0.00
2	2	FB	M	2	3	0.44
2	3	S	F	1	3	0.00
2	3	S	M	1	3	0.04
2	3	S	F	2	3	0.00
2	3	S	M	2	3	0.07
2	4	FC	F	1	3	0.00
2	4	FC	M	1	3	0.30
2	4	FC	F	2	3	0.05
2	4	FC	M	2	3	0.68
2	5	FA	F	1	3	0.08
2	5	FA	M	1	3	0.46
2	5	FA	F	2	3	0.09
2	5	FA	M	2	3	0.85
2	6	FB	F	1	3	0.06
2	6	FB	M	1	3	0.25
2	6	FB	F	2	3	0.11
2	6	FB	M	2	3	0.57
2	7	S	F	1	3	0.00
2	7	S	M	1	3	0.00
2	7	S	F	2	3	0.00
2	7	S	M	2	3	0.13

2	8	FC	F	1	3	0.03
2	8	FC	M	1	3	0.43
2	8	FC	F	2	3	0.16
2	8	FC	M	2	3	0.56
2	9	FB	F	1	3	0.04
2	9	FB	M	1	3	0.18
2	9	FB	F	2	3	0.14
2	9	FB	M	2	3	0.57
2	10	S	F	1	3	0.00
2	10	S	M	1	3	0.00
2	10	S	F	2	3	0.00
2	10	S	M	2	3	0.04
2	11	FC	F	1	3	0.00
2	11	FC	M	1	3	0.18
2	11	FC	F	2	3	0.04
2	11	FC	M	2	3	0.48
2	12	FA	F	1	3	0.10
2	12	FA	M	1	3	0.30
2	12	FA	F	2	3	0.04
2	12	FA	M	2	3	0.70
2	13	FB	F	1	3	0.04
2	13	FB	M	1	3	0.21
2	13	FB	F	2	3	0.15
2	13	FB	M	2	3	0.61
2	14	S	F	1	3	0.00
2	14	S	M	1	3	0.00
2	14	S	F	2	3	0.00
2	14	S	M	2	3	0.03
2	15	FC	F	1	3	0.04
2	15	FC	M	1	3	0.16
2	15	FC	F	2	3	0.00
2	15	FC	M	2	3	0.48
2	16	FA	F	1	3	0.04
2	16	FA	M	1	3	0.22
2	16	FA	F	2	3	0.18
2	16	FA	M	2	3	0.67
1	1	FB	F	1	4	0.00
1	1	FB	M	1	4	0.00
1	1	FB	F	2	4	0.00
1	1	FB	M	2	4	0.00
1	2	FA	F	1	4	0.00
1	2	FA	M	1	4	0.05
1	2	FA	F	2	4	0.00
1	2	FA	M	2	4	0.00
1	3	FC	F	1	4	0.00
1	3	FC	M	1	4	0.00
1	3	FC	F	2	4	0.00
1	3	FC	M	2	4	0.00
1	4	S	F	1	4	0.00
1	4	S	M	1	4	0.00
1	4	S	F	2	4	0.00
1	4	S	M	2	4	0.00
1	5	FB	F	1	4	0.00

1	5	FB	M	1	4	0.00
1	5	FB	F	2	4	0.00
1	5	FB	M	2	4	0.00
1	6	FA	F	1	4	0.00
1	6	FA	M	1	4	0.00
1	6	FA	F	2	4	0.00
1	6	FA	M	2	4	0.00
1	7	FC	F	1	4	0.00
1	7	FC	M	1	4	0.00
1	7	FC	F	2	4	0.00
1	7	FC	M	2	4	0.00
1	8	S	F	1	4	0.00
1	8	S	M	1	4	0.00
1	8	S	F	2	4	0.00
1	8	S	M	2	4	0.00
1	9	S	F	1	4	0.00
1	9	S	M	1	4	0.00
1	9	S	F	2	4	0.00
1	9	S	M	2	4	0.00
1	10	FB	F	1	4	0.00
1	10	FB	M	1	4	0.00
1	10	FB	F	2	4	0.00
1	10	FB	M	2	4	0.00
1	11	FA	F	1	4	0.04
1	11	FA	M	1	4	0.00
1	11	FA	F	2	4	0.00
1	11	FA	M	2	4	0.00
1	12	FC	F	1	4	0.00
1	12	FC	M	1	4	0.00
1	12	FC	F	2	4	0.00
1	12	FC	M	2	4	0.00
1	13	S	F	1	4	0.00
1	13	S	M	1	4	0.00
1	13	S	F	2	4	0.00
1	13	S	M	2	4	0.00
1	14	FB	F	1	4	0.04
1	14	FB	M	1	4	0.00
1	14	FB	F	2	4	0.00
1	14	FB	M	2	4	0.00
1	15	FA	F	1	4	0.00
1	15	FA	M	1	4	0.00
1	15	FA	F	2	4	0.00
1	15	FA	M	2	4	0.00
1	16	FC	F	1	4	0.00
1	16	FC	M	1	4	0.00
1	16	FC	F	2	4	0.00
1	16	FC	M	2	4	0.00
2	1	FA	F	1	4	0.00
2	1	FA	M	1	4	0.00
2	1	FA	F	2	4	0.00
2	1	FA	M	2	4	0.05
2	2	FB	F	1	4	0.00
2	2	FB	M	1	4	0.00

2	2	FB	F	2	4	0.00
2	2	FB	M	2	4	0.00
2	3	S	F	1	4	0.00
2	3	S	M	1	4	0.00
2	3	S	F	2	4	0.00
2	3	S	M	2	4	0.00
2	4	FC	F	1	4	0.00
2	4	FC	M	1	4	0.00
2	4	FC	F	2	4	0.00
2	4	FC	M	2	4	0.00
2	5	FA	F	1	4	0.00
2	5	FA	M	1	4	0.00
2	5	FA	F	2	4	0.00
2	5	FA	M	2	4	0.00
2	6	FB	F	1	4	0.00
2	6	FB	M	1	4	0.00
2	6	FB	F	2	4	0.00
2	6	FB	M	2	4	0.04
2	7	S	F	1	4	0.00
2	7	S	M	1	4	0.00
2	7	S	F	2	4	0.00
2	7	S	M	2	4	0.00
2	8	FC	F	1	4	0.00
2	8	FC	M	1	4	0.00
2	8	FC	F	2	4	0.00
2	8	FC	M	2	4	0.00
2	9	FB	F	1	4	0.00
2	9	FB	M	1	4	0.00
2	9	FB	F	2	4	0.00
2	9	FB	M	2	4	0.00
2	10	S	F	1	4	0.00
2	10	S	M	1	4	0.00
2	10	S	F	2	4	0.00
2	10	S	M	2	4	0.00
2	11	FC	F	1	4	0.00
2	11	FC	M	1	4	0.00
2	11	FC	F	2	4	0.00
2	11	FC	M	2	4	0.00
2	12	FA	F	1	4	0.00
2	12	FA	M	1	4	0.05
2	12	FA	F	2	4	0.00
2	12	FA	M	2	4	0.00
2	13	FB	F	1	4	0.00
2	13	FB	M	1	4	0.00
2	13	FB	F	2	4	0.00
2	13	FB	M	2	4	0.00
2	14	S	F	1	4	0.00
2	14	S	M	1	4	0.00
2	14	S	F	2	4	0.00
2	14	S	M	2	4	0.00
2	15	FC	F	1	4	0.00
2	15	FC	M	1	4	0.00
2	15	FC	F	2	4	0.00

2	15	FC	M	2	4	0.00
2	16	FA	F	1	4	0.00
2	16	FA	M	1	4	0.00
2	16	FA	F	2	4	0.00
2	16	FA	M	2	4	0.00
1	1	FB	F	1	5	0.00
1	1	FB	M	1	5	0.00
1	1	FB	F	2	5	0.00
1	1	FB	M	2	5	0.00
1	2	FA	F	1	5	0.00
1	2	FA	M	1	5	0.00
1	2	FA	F	2	5	0.00
1	2	FA	M	2	5	0.00
1	3	FC	F	1	5	0.00
1	3	FC	M	1	5	0.00
1	3	FC	F	2	5	0.00
1	3	FC	M	2	5	0.00
1	4	S	F	1	5	0.00
1	4	S	M	1	5	0.00
1	4	S	F	2	5	0.00
1	4	S	M	2	5	0.00
1	5	FB	F	1	5	0.00
1	5	FB	M	1	5	0.00
1	5	FB	F	2	5	0.00
1	5	FB	M	2	5	0.00
1	6	FA	F	1	5	0.00
1	6	FA	M	1	5	0.00
1	6	FA	F	2	5	0.00
1	6	FA	M	2	5	0.00
1	7	FC	F	1	5	0.00
1	7	FC	M	1	5	0.00
1	7	FC	F	2	5	0.00
1	7	FC	M	2	5	0.00
1	8	S	F	1	5	0.00
1	8	S	M	1	5	0.00
1	8	S	F	2	5	0.00
1	8	S	M	2	5	0.00
1	9	S	F	1	5	0.00
1	9	S	M	1	5	0.00
1	9	S	F	2	5	0.00
1	9	S	M	2	5	0.00
1	10	FB	F	1	5	0.00
1	10	FB	M	1	5	0.00
1	10	FB	F	2	5	0.00
1	10	FB	M	2	5	0.00
1	11	FA	F	1	5	0.00
1	11	FA	M	1	5	0.04
1	11	FA	F	2	5	0.00
1	11	FA	M	2	5	0.00
1	12	FC	F	1	5	0.00
1	12	FC	M	1	5	0.00
1	12	FC	F	2	5	0.00
1	12	FC	M	2	5	0.00

1	13	S	F	1	5	0.00
1	13	S	M	1	5	0.00
1	13	S	F	2	5	0.00
1	13	S	M	2	5	0.00
1	14	FB	F	1	5	0.04
1	14	FB	M	1	5	0.00
1	14	FB	F	2	5	0.00
1	14	FB	M	2	5	0.00
1	15	FA	F	1	5	0.00
1	15	FA	M	1	5	0.00
1	15	FA	F	2	5	0.00
1	15	FA	M	2	5	0.00
1	16	FC	F	1	5	0.00
1	16	FC	M	1	5	0.00
1	16	FC	F	2	5	0.00
1	16	FC	M	2	5	0.00
2	1	FA	F	1	5	0.00
2	1	FA	M	1	5	0.05
2	1	FA	F	2	5	0.00
2	1	FA	M	2	5	0.00
2	2	FB	F	1	5	0.00
2	2	FB	M	1	5	0.00
2	2	FB	F	2	5	0.00
2	2	FB	M	2	5	0.00
2	3	S	F	1	5	0.00
2	3	S	M	1	5	0.00
2	3	S	F	2	5	0.00
2	3	S	M	2	5	0.00
2	4	FC	F	1	5	0.00
2	4	FC	M	1	5	0.00
2	4	FC	F	2	5	0.00
2	4	FC	M	2	5	0.00
2	5	FA	F	1	5	0.00
2	5	FA	M	1	5	0.00
2	5	FA	F	2	5	0.00
2	5	FA	M	2	5	0.00
2	6	FB	F	1	5	0.00
2	6	FB	M	1	5	0.00
2	6	FB	F	2	5	0.00
2	6	FB	M	2	5	0.00
2	7	S	F	1	5	0.00
2	7	S	M	1	5	0.04
2	7	S	F	2	5	0.00
2	7	S	M	2	5	0.00
2	8	FC	F	1	5	0.00
2	8	FC	M	1	5	0.00
2	8	FC	F	2	5	0.00
2	8	FC	M	2	5	0.00
2	9	FB	F	1	5	0.00
2	9	FB	M	1	5	0.00
2	9	FB	F	2	5	0.00
2	9	FB	M	2	5	0.00
2	10	S	F	1	5	0.00

2	10	S	M	1	5	0.00
2	10	S	F	2	5	0.00
2	10	S	M	2	5	0.00
2	11	FC	F	1	5	0.00
2	11	FC	M	1	5	0.00
2	11	FC	F	2	5	0.00
2	11	FC	M	2	5	0.00
2	12	FA	F	1	5	0.03
2	12	FA	M	1	5	0.00
2	12	FA	F	2	5	0.00
2	12	FA	M	2	5	0.00
2	13	FB	F	1	5	0.00
2	13	FB	M	1	5	0.05
2	13	FB	F	2	5	0.00
2	13	FB	M	2	5	0.00
2	14	S	F	1	5	0.00
2	14	S	M	1	5	0.00
2	14	S	F	2	5	0.00
2	14	S	M	2	5	0.00
2	15	FC	F	1	5	0.00
2	15	FC	M	1	5	0.00
2	15	FC	F	2	5	0.00
2	15	FC	M	2	5	0.00
2	16	FA	F	1	5	0.00
2	16	FA	M	1	5	0.00
2	16	FA	F	2	5	0.00
2	16	FA	M	2	5	0.00
