# Supplementary material for: Slow and steady wins the race: The behaviour and welfare of commercial faster growing broiler breeds compared to a commercial slower growing breed
Source: PLoS One. 2020 Apr 6;15(4):e0231006. doi: 10.1371/journal.pone.0231006 (PMC7135253; doi:10.1371/journal.pone.0231006)
Supplement: S6 Data — (PDF) [file pone.0231006.s006.pdf]

Replicate	Pen	Breed	Sex	WA	Feather Score	Prop
1	1	FB	F	1	0	0.00
1	1	FB	M	1	0	0.00
1	1	FB	F	2	0	0.00
1	1	FB	M	2	0	0.00
1	2	FA	F	1	0	0.00
1	2	FA	M	1	0	0.00
1	2	FA	F	2	0	0.00
1	2	FA	M	2	0	0.00
1	3	FC	F	1	0	0.00
1	3	FC	M	1	0	0.00
1	3	FC	F	2	0	0.00
1	3	FC	M	2	0	0.00
1	4	S	F	1	0	0.75
1	4	S	M	1	0	0.54
1	4	S	F	2	0	1.00
1	4	S	M	2	0	0.90
1	5	FB	F	1	0	0.00
1	5	FB	M	1	0	0.00
1	5	FB	F	2	0	0.11
1	5	FB	M	2	0	0.13
1	6	FA	F	1	0	0.00
1	6	FA	M	1	0	0.00
1	6	FA	F	2	0	0.00
1	6	FA	M	2	0	0.00
1	7	FC	F	1	0	0.00
1	7	FC	M	1	0	0.00
1	7	FC	F	2	0	0.00
1	7	FC	M	2	0	0.00
1	8	S	F	1	0	0.92
1	8	S	M	1	0	0.42
1	8	S	F	2	0	1.00
1	8	S	M	2	0	0.78
1	9	S	F	1	0	1.00
1	9	S	M	1	0	1.00
1	9	S	F	2	0	1.00
1	9	S	M	2	0	0.94
1	10	FB	F	1	0	0.00
1	10	FB	M	1	0	0.00
1	10	FB	F	2	0	0.00
1	10	FB	M	2	0	0.00
1	11	FA	F	1	0	0.00
1	11	FA	M	1	0	0.00
1	11	FA	F	2	0	0.00
1	11	FA	M	2	0	0.00
1	12	FC	F	1	0	0.00
1	12	FC	M	1	0	0.00
1	12	FC	F	2	0	0.00
1	12	FC	M	2	0	0.00
1	13	S	F	1	0	0.75
1	13	S	M	1	0	0.87
1	13	S	F	2	0	0.79
1	13	S	M	2	0	0.73

1	14	FB	F	1	0	0.00
1	14	FB	M	1	0	0.00
1	14	FB	F	2	0	0.00
1	14	FB	M	2	0	0.00
1	15	FA	F	1	0	0.00
1	15	FA	M	1	0	0.00
1	15	FA	F	2	0	0.00
1	15	FA	M	2	0	0.00
1	16	FC	F	1	0	0.00
1	16	FC	M	1	0	0.00
1	16	FC	F	2	0	0.00
1	16	FC	M	2	0	0.00
2	1	FA	F	1	0	0.00
2	1	FA	M	1	0	0.00
2	1	FA	F	2	0	0.00
2	1	FA	M	2	0	0.00
2	2	FB	F	1	0	0.00
2	2	FB	M	1	0	0.00
2	2	FB	F	2	0	0.00
2	2	FB	M	2	0	0.00
2	3	S	F	1	0	0.58
2	3	S	M	1	0	0.46
2	3	S	F	2	0	0.90
2	3	S	M	2	0	0.53
2	4	FC	F	1	0	0.00
2	4	FC	M	1	0	0.00
2	4	FC	F	2	0	0.00
2	4	FC	M	2	0	0.00
2	5	FA	F	1	0	0.00
2	5	FA	M	1	0	0.00
2	5	FA	F	2	0	0.00
2	5	FA	M	2	0	0.00
2	6	FB	F	1	0	0.00
2	6	FB	M	1	0	0.00
2	6	FB	F	2	0	0.00
2	6	FB	M	2	0	0.00
2	7	S	F	1	0	0.92
2	7	S	M	1	0	0.54
2	7	S	F	2	0	1.00
2	7	S	M	2	0	0.36
2	8	FC	F	1	0	0.00
2	8	FC	M	1	0	0.00
2	8	FC	F	2	0	0.00
2	8	FC	M	2	0	0.00
2	9	FB	F	1	0	0.00
2	9	FB	M	1	0	0.00
2	9	FB	F	2	0	0.00
2	9	FB	M	2	0	0.00
2	10	S	F	1	0	0.78
2	10	S	M	1	0	0.56
2	10	S	F	2	0	0.88
2	10	S	M	2	0	0.47
2	11	FC	F	1	0	0.00

2	11	FC	M	1	0	0.00
2	11	FC	F	2	0	0.00
2	11	FC	M	2	0	0.00
2	12	FA	F	1	0	0.00
2	12	FA	M	1	0	0.00
2	12	FA	F	2	0	0.00
2	12	FA	M	2	0	0.00
2	13	FB	F	1	0	0.00
2	13	FB	M	1	0	0.00
2	13	FB	F	2	0	0.00
2	13	FB	M	2	0	0.00
2	14	S	F	1	0	1.00
2	14	S	M	1	0	0.44
2	14	S	F	2	0	0.64
2	14	S	M	2	0	0.43
2	15	FC	F	1	0	0.00
2	15	FC	M	1	0	0.00
2	15	FC	F	2	0	0.00
2	15	FC	M	2	0	0.00
2	16	FA	F	1	0	0.08
2	16	FA	M	1	0	0.00
2	16	FA	F	2	0	0.00
2	16	FA	M	2	0	0.00
1	1	FB	F	1	0.5	0.31
1	1	FB	M	1	0.5	0.00
1	1	FB	F	2	0.5	0.44
1	1	FB	M	2	0.5	0.22
1	2	FA	F	1	0.5	0.64
1	2	FA	M	1	0.5	0.45
1	2	FA	F	2	0.5	0.45
1	2	FA	M	2	0.5	0.00
1	3	FC	F	1	0.5	0.00
1	3	FC	M	1	0.5	0.13
1	3	FC	F	2	0.5	0.15
1	3	FC	M	2	0.5	0.08
1	4	S	F	1	0.5	0.25
1	4	S	M	1	0.5	0.46
1	4	S	F	2	0.5	0.00
1	4	S	M	2	0.5	0.10
1	5	FB	F	1	0.5	0.20
1	5	FB	M	1	0.5	0.27
1	5	FB	F	2	0.5	0.56
1	5	FB	M	2	0.5	0.31
1	6	FA	F	1	0.5	0.27
1	6	FA	M	1	0.5	0.21
1	6	FA	F	2	0.5	0.17
1	6	FA	M	2	0.5	0.08
1	7	FC	F	1	0.5	0.15
1	7	FC	M	1	0.5	0.00
1	7	FC	F	2	0.5	0.29
1	7	FC	M	2	0.5	0.36
1	8	S	F	1	0.5	0.08
1	8	S	M	1	0.5	0.58

1	8	S	F	2	0.5	0.00
1	8	S	M	2	0.5	0.22
1	9	S	F	1	0.5	0.00
1	9	S	M	1	0.5	0.00
1	9	S	F	2	0.5	0.00
1	9	S	M	2	0.5	0.06
1	10	FB	F	1	0.5	0.38
1	10	FB	M	1	0.5	0.36
1	10	FB	F	2	0.5	0.57
1	10	FB	M	2	0.5	0.27
1	11	FA	F	1	0.5	0.50
1	11	FA	M	1	0.5	0.07
1	11	FA	F	2	0.5	0.31
1	11	FA	M	2	0.5	0.08
1	12	FC	F	1	0.5	0.00
1	12	FC	M	1	0.5	0.07
1	12	FC	F	2	0.5	0.54
1	12	FC	M	2	0.5	0.27
1	13	S	F	1	0.5	0.17
1	13	S	M	1	0.5	0.13
1	13	S	F	2	0.5	0.21
1	13	S	M	2	0.5	0.27
1	14	FB	F	1	0.5	0.38
1	14	FB	M	1	0.5	0.25
1	14	FB	F	2	0.5	0.40
1	14	FB	M	2	0.5	0.41
1	15	FA	F	1	0.5	0.42
1	15	FA	M	1	0.5	0.31
1	15	FA	F	2	0.5	0.45
1	15	FA	M	2	0.5	0.29
1	16	FC	F	1	0.5	0.40
1	16	FC	M	1	0.5	0.40
1	16	FC	F	2	0.5	0.27
1	16	FC	M	2	0.5	0.08
2	1	FA	F	1	0.5	0.25
2	1	FA	M	1	0.5	0.14
2	1	FA	F	2	0.5	0.31
2	1	FA	M	2	0.5	0.17
2	2	FB	F	1	0.5	0.18
2	2	FB	M	1	0.5	0.21
2	2	FB	F	2	0.5	0.50
2	2	FB	M	2	0.5	0.54
2	3	S	F	1	0.5	0.42
2	3	S	M	1	0.5	0.54
2	3	S	F	2	0.5	0.10
2	3	S	M	2	0.5	0.47
2	4	FC	F	1	0.5	0.00
2	4	FC	M	1	0.5	0.20
2	4	FC	F	2	0.5	0.00
2	4	FC	M	2	0.5	0.38
2	5	FA	F	1	0.5	0.31
2	5	FA	M	1	0.5	0.50
2	5	FA	F	2	0.5	0.31

2	5	FA	M	2	0.5	0.33
2	6	FB	F	1	0.5	0.40
2	6	FB	M	1	0.5	0.27
2	6	FB	F	2	0.5	0.60
2	6	FB	M	2	0.5	0.20
2	7	S	F	1	0.5	0.08
2	7	S	M	1	0.5	0.54
2	7	S	F	2	0.5	0.00
2	7	S	M	2	0.5	0.50
2	8	FC	F	1	0.5	0.13
2	8	FC	M	1	0.5	0.00
2	8	FC	F	2	0.5	0.17
2	8	FC	M	2	0.5	0.23
2	9	FB	F	1	0.5	0.00
2	9	FB	M	1	0.5	0.08
2	9	FB	F	2	0.5	0.08
2	9	FB	M	2	0.5	0.17
2	10	S	F	1	0.5	0.22
2	10	S	M	1	0.5	0.44
2	10	S	F	2	0.5	0.13
2	10	S	M	2	0.5	0.53
2	11	FC	F	1	0.5	0.08
2	11	FC	M	1	0.5	0.17
2	11	FC	F	2	0.5	0.14
2	11	FC	M	2	0.5	0.09
2	12	FA	F	1	0.5	0.21
2	12	FA	M	1	0.5	0.00
2	12	FA	F	2	0.5	0.42
2	12	FA	M	2	0.5	0.31
2	13	FB	F	1	0.5	0.00
2	13	FB	M	1	0.5	0.23
2	13	FB	F	2	0.5	0.50
2	13	FB	M	2	0.5	0.18
2	14	S	F	1	0.5	0.22
2	14	S	M	1	0.5	0.56
2	14	S	F	2	0.5	0.18
2	14	S	M	2	0.5	0.57
2	15	FC	F	1	0.5	0.08
2	15	FC	M	1	0.5	0.08
2	15	FC	F	2	0.5	0.00
2	15	FC	M	2	0.5	0.19
2	16	FA	F	1	0.5	0.17
2	16	FA	M	1	0.5	0.15
2	16	FA	F	2	0.5	0.38
2	16	FA	M	2	0.5	0.17
1	1	FB	F	1	1	0.62
1	1	FB	M	1	1	1.00
1	1	FB	F	2	1	0.56
1	1	FB	M	2	1	0.78
1	2	FA	F	1	1	0.36
1	2	FA	M	1	1	0.55
1	2	FA	F	2	1	0.27
1	2	FA	M	2	1	0.36

1	3	FC	F	1	1	1.00
1	3	FC	M	1	1	0.73
1	3	FC	F	2	1	0.77
1	3	FC	M	2	1	0.83
1	4	S	F	1	1	0.00
1	4	S	M	1	1	0.00
1	4	S	F	2	1	0.00
1	4	S	M	2	1	0.00
1	5	FB	F	1	1	0.80
1	5	FB	M	1	1	0.73
1	5	FB	F	2	1	0.33
1	5	FB	M	2	1	0.56
1	6	FA	F	1	1	0.73
1	6	FA	M	1	1	0.79
1	6	FA	F	2	1	0.67
1	6	FA	M	2	1	0.50
1	7	FC	F	1	1	0.85
1	7	FC	M	1	1	0.42
1	7	FC	F	2	1	0.71
1	7	FC	M	2	1	0.55
1	8	S	F	1	1	0.00
1	8	S	M	1	1	0.00
1	8	S	F	2	1	0.00
1	8	S	M	2	1	0.00
1	9	S	F	1	1	0.00
1	9	S	M	1	1	0.00
1	9	S	F	2	1	0.00
1	9	S	M	2	1	0.00
1	10	FB	F	1	1	0.63
1	10	FB	M	1	1	0.64
1	10	FB	F	2	1	0.43
1	10	FB	M	2	1	0.73
1	11	FA	F	1	1	0.50
1	11	FA	M	1	1	0.93
1	11	FA	F	2	1	0.69
1	11	FA	M	2	1	0.92
1	12	FC	F	1	1	0.90
1	12	FC	M	1	1	0.93
1	12	FC	F	2	1	0.46
1	12	FC	M	2	1	0.73
1	13	S	F	1	1	0.08
1	13	S	M	1	1	0.00
1	13	S	F	2	1	0.00
1	13	S	M	2	1	0.00
1	14	FB	F	1	1	0.54
1	14	FB	M	1	1	0.75
1	14	FB	F	2	1	0.50
1	14	FB	M	2	1	0.59
1	15	FA	F	1	1	0.50
1	15	FA	M	1	1	0.23
1	15	FA	F	2	1	0.55
1	15	FA	M	2	1	0.43
1	16	FC	F	1	1	0.53

1	16	FC	M	1	1	0.50
1	16	FC	F	2	1	0.73
1	16	FC	M	2	1	0.92
2	1	FA	F	1	1	0.75
2	1	FA	M	1	1	0.57
2	1	FA	F	2	1	0.69
2	1	FA	M	2	1	0.67
2	2	FB	F	1	1	0.82
2	2	FB	M	1	1	0.43
2	2	FB	F	2	1	0.50
2	2	FB	M	2	1	0.46
2	3	S	F	1	1	0.00
2	3	S	M	1	1	0.00
2	3	S	F	2	1	0.00
2	3	S	M	2	1	0.00
2	4	FC	F	1	1	1.00
2	4	FC	M	1	1	0.73
2	4	FC	F	2	1	1.00
2	4	FC	M	2	1	0.62
2	5	FA	F	1	1	0.62
2	5	FA	M	1	1	0.42
2	5	FA	F	2	1	0.69
2	5	FA	M	2	1	0.58
2	6	FB	F	1	1	0.40
2	6	FB	M	1	1	0.53
2	6	FB	F	2	1	0.40
2	6	FB	M	2	1	0.80
2	7	S	F	1	1	0.00
2	7	S	M	1	1	0.08
2	7	S	F	2	1	0.00
2	7	S	M	2	1	0.00
2	8	FC	F	1	1	0.81
2	8	FC	M	1	1	0.89
2	8	FC	F	2	1	0.83
2	8	FC	M	2	1	0.77
2	9	FB	F	1	1	0.77
2	9	FB	M	1	1	0.50
2	9	FB	F	2	1	0.92
2	9	FB	M	2	1	0.67
2	10	S	F	1	1	0.00
2	10	S	M	1	1	0.00
2	10	S	F	2	1	0.00
2	10	S	M	2	1	0.00
2	11	FC	F	1	1	0.85
2	11	FC	M	1	1	0.58
2	11	FC	F	2	1	0.79
2	11	FC	M	2	1	0.73
2	12	FA	F	1	1	0.79
2	12	FA	M	1	1	0.82
2	12	FA	F	2	1	0.58
2	12	FA	M	2	1	0.69
2	13	FB	F	1	1	0.75
2	13	FB	M	1	1	0.46

2	13	FB	F	2	1	0.50
2	13	FB	M	2	1	0.82
2	14	S	F	1	1	0.00
2	14	S	M	1	1	0.00
2	14	S	F	2	1	0.00
2	14	S	M	2	1	0.00
2	15	FC	F	1	1	0.83
2	15	FC	M	1	1	0.69
2	15	FC	F	2	1	0.89
2	15	FC	M	2	1	0.63
2	16	FA	F	1	1	0.50
2	16	FA	M	1	1	0.46
2	16	FA	F	2	1	0.62
2	16	FA	M	2	1	0.75
1	1	FB	F	1	1.5	0.00
1	1	FB	M	1	1.5	0.00
1	1	FB	F	2	1.5	0.00
1	1	FB	M	2	1.5	0.00
1	2	FA	F	1	1.5	0.00
1	2	FA	M	1	1.5	0.00
1	2	FA	F	2	1.5	0.27
1	2	FA	M	2	1.5	0.64
1	3	FC	F	1	1.5	0.00
1	3	FC	M	1	1.5	0.13
1	3	FC	F	2	1.5	0.08
1	3	FC	M	2	1.5	0.08
1	4	S	F	1	1.5	0.00
1	4	S	M	1	1.5	0.00
1	4	S	F	2	1.5	0.00
1	4	S	M	2	1.5	0.00
1	5	FB	F	1	1.5	0.00
1	5	FB	M	1	1.5	0.00
1	5	FB	F	2	1.5	0.00
1	5	FB	M	2	1.5	0.00
1	6	FA	F	1	1.5	0.00
1	6	FA	M	1	1.5	0.00
1	6	FA	F	2	1.5	0.17
1	6	FA	M	2	1.5	0.42
1	7	FC	F	1	1.5	0.00
1	7	FC	M	1	1.5	0.58
1	7	FC	F	2	1.5	0.00
1	7	FC	M	2	1.5	0.09
1	8	S	F	1	1.5	0.00
1	8	S	M	1	1.5	0.00
1	8	S	F	2	1.5	0.00
1	8	S	M	2	1.5	0.00
1	9	S	F	1	1.5	0.00
1	9	S	M	1	1.5	0.00
1	9	S	F	2	1.5	0.00
1	9	S	M	2	1.5	0.00
1	10	FB	F	1	1.5	0.00
1	10	FB	M	1	1.5	0.00
1	10	FB	F	2	1.5	0.00

1	10	FB	M	2	1.5	0.00
1	11	FA	F	1	1.5	0.00
1	11	FA	M	1	1.5	0.00
1	11	FA	F	2	1.5	0.00
1	11	FA	M	2	1.5	0.00
1	12	FC	F	1	1.5	0.00
1	12	FC	M	1	1.5	0.00
1	12	FC	F	2	1.5	0.00
1	12	FC	M	2	1.5	0.00
1	13	S	F	1	1.5	0.00
1	13	S	M	1	1.5	0.00
1	13	S	F	2	1.5	0.00
1	13	S	M	2	1.5	0.00
1	14	FB	F	1	1.5	0.08
1	14	FB	M	1	1.5	0.00
1	14	FB	F	2	1.5	0.10
1	14	FB	M	2	1.5	0.00
1	15	FA	F	1	1.5	0.08
1	15	FA	M	1	1.5	0.46
1	15	FA	F	2	1.5	0.00
1	15	FA	M	2	1.5	0.29
1	16	FC	F	1	1.5	0.07
1	16	FC	M	1	1.5	0.10
1	16	FC	F	2	1.5	0.00
1	16	FC	M	2	1.5	0.00
2	1	FA	F	1	1.5	0.00
2	1	FA	M	1	1.5	0.29
2	1	FA	F	2	1.5	0.00
2	1	FA	M	2	1.5	0.17
2	2	FB	F	1	1.5	0.00
2	2	FB	M	1	1.5	0.36
2	2	FB	F	2	1.5	0.00
2	2	FB	M	2	1.5	0.00
2	3	S	F	1	1.5	0.00
2	3	S	M	1	1.5	0.00
2	3	S	F	2	1.5	0.00
2	3	S	M	2	1.5	0.00
2	4	FC	F	1	1.5	0.00
2	4	FC	M	1	1.5	0.07
2	4	FC	F	2	1.5	0.00
2	4	FC	M	2	1.5	0.00
2	5	FA	F	1	1.5	0.08
2	5	FA	M	1	1.5	0.08
2	5	FA	F	2	1.5	0.00
2	5	FA	M	2	1.5	0.08
2	6	FB	F	1	1.5	0.20
2	6	FB	M	1	1.5	0.20
2	6	FB	F	2	1.5	0.00
2	6	FB	M	2	1.5	0.00
2	7	S	F	1	1.5	0.00
2	7	S	M	1	1.5	0.00
2	7	S	F	2	1.5	0.00
2	7	S	M	2	1.5	0.00

2	8	FC	F	1	1.5	0.06
2	8	FC	M	1	1.5	0.11
2	8	FC	F	2	1.5	0.00
2	8	FC	M	2	1.5	0.00
2	9	FB	F	1	1.5	0.23
2	9	FB	M	1	1.5	0.42
2	9	FB	F	2	1.5	0.00
2	9	FB	M	2	1.5	0.17
2	10	S	F	1	1.5	0.00
2	10	S	M	1	1.5	0.00
2	10	S	F	2	1.5	0.00
2	10	S	M	2	1.5	0.00
2	11	FC	F	1	1.5	0.08
2	11	FC	M	1	1.5	0.25
2	11	FC	F	2	1.5	0.07
2	11	FC	M	2	1.5	0.18
2	12	FA	F	1	1.5	0.00
2	12	FA	M	1	1.5	0.18
2	12	FA	F	2	1.5	0.00
2	12	FA	M	2	1.5	0.00
2	13	FB	F	1	1.5	0.25
2	13	FB	M	1	1.5	0.31
2	13	FB	F	2	1.5	0.00
2	13	FB	M	2	1.5	0.00
2	14	S	F	1	1.5	0.00
2	14	S	M	1	1.5	0.00
2	14	S	F	2	1.5	0.00
2	14	S	M	2	1.5	0.00
2	15	FC	F	1	1.5	0.08
2	15	FC	M	1	1.5	0.23
2	15	FC	F	2	1.5	0.11
2	15	FC	M	2	1.5	0.19
2	16	FA	F	1	1.5	0.25
2	16	FA	M	1	1.5	0.38
2	16	FA	F	2	1.5	0.00
2	16	FA	M	2	1.5	0.08
1	1	FB	F	1	2	0.15
1	1	FB	M	1	2	0.00
1	1	FB	F	2	2	0.00
1	1	FB	M	2	2	0.00
1	2	FA	F	1	2	0.00
1	2	FA	M	1	2	0.00
1	2	FA	F	2	2	0.00
1	2	FA	M	2	2	0.00
1	3	FC	F	1	2	0.00
1	3	FC	M	1	2	0.00
1	3	FC	F	2	2	0.00
1	3	FC	M	2	2	0.00
1	4	S	F	1	2	0.00
1	4	S	M	1	2	0.00
1	4	S	F	2	2	0.00
1	4	S	M	2	2	0.00
1	5	FB	F	1	2	0.00

1	5	FB	M	1	2	0.00
1	5	FB	F	2	2	0.00
1	5	FB	M	2	2	0.00
1	6	FA	F	1	2	0.00
1	6	FA	M	1	2	0.00
1	6	FA	F	2	2	0.00
1	6	FA	M	2	2	0.00
1	7	FC	F	1	2	0.00
1	7	FC	M	1	2	0.00
1	7	FC	F	2	2	0.00
1	7	FC	M	2	2	0.00
1	8	S	F	1	2	0.00
1	8	S	M	1	2	0.00
1	8	S	F	2	2	0.00
1	8	S	M	2	2	0.00
1	9	S	F	1	2	0.00
1	9	S	M	1	2	0.00
1	9	S	F	2	2	0.00
1	9	S	M	2	2	0.00
1	10	FB	F	1	2	0.00
1	10	FB	M	1	2	0.00
1	10	FB	F	2	2	0.00
1	10	FB	M	2	2	0.00
1	11	FA	F	1	2	0.00
1	11	FA	M	1	2	0.00
1	11	FA	F	2	2	0.00
1	11	FA	M	2	2	0.00
1	12	FC	F	1	2	0.20
1	12	FC	M	1	2	0.00
1	12	FC	F	2	2	0.00
1	12	FC	M	2	2	0.00
1	13	S	F	1	2	0.00
1	13	S	M	1	2	0.00
1	13	S	F	2	2	0.00
1	13	S	M	2	2	0.00
1	14	FB	F	1	2	0.00
1	14	FB	M	1	2	0.00
1	14	FB	F	2	2	0.00
1	14	FB	M	2	2	0.00
1	15	FA	F	1	2	0.00
1	15	FA	M	1	2	0.00
1	15	FA	F	2	2	0.00
1	15	FA	M	2	2	0.00
1	16	FC	F	1	2	0.00
1	16	FC	M	1	2	0.00
1	16	FC	F	2	2	0.00
1	16	FC	M	2	2	0.00
2	1	FA	F	1	2	0.00
2	1	FA	M	1	2	0.00
2	1	FA	F	2	2	0.00
2	1	FA	M	2	2	0.00
2	2	FB	F	1	2	0.00
2	2	FB	M	1	2	0.00

2	2	FB	F	2	2	0.00
2	2	FB	M	2	2	0.00
2	3	S	F	1	2	0.00
2	3	S	M	1	2	0.00
2	3	S	F	2	2	0.00
2	3	S	M	2	2	0.00
2	4	FC	F	1	2	0.00
2	4	FC	M	1	2	0.00
2	4	FC	F	2	2	0.00
2	4	FC	M	2	2	0.00
2	5	FA	F	1	2	0.00
2	5	FA	M	1	2	0.00
2	5	FA	F	2	2	0.00
2	5	FA	M	2	2	0.00
2	6	FB	F	1	2	0.00
2	6	FB	M	1	2	0.00
2	6	FB	F	2	2	0.00
2	6	FB	M	2	2	0.00
2	7	S	F	1	2	0.00
2	7	S	M	1	2	0.00
2	7	S	F	2	2	0.00
2	7	S	M	2	2	0.00
2	8	FC	F	1	2	0.00
2	8	FC	M	1	2	0.00
2	8	FC	F	2	2	0.00
2	8	FC	M	2	2	0.00
2	9	FB	F	1	2	0.00
2	9	FB	M	1	2	0.00
2	9	FB	F	2	2	0.00
2	9	FB	M	2	2	0.00
2	10	S	F	1	2	0.00
2	10	S	M	1	2	0.00
2	10	S	F	2	2	0.00
2	10	S	M	2	2	0.00
2	11	FC	F	1	2	0.00
2	11	FC	M	1	2	0.00
2	11	FC	F	2	2	0.00
2	11	FC	M	2	2	0.00
2	12	FA	F	1	2	0.00
2	12	FA	M	1	2	0.00
2	12	FA	F	2	2	0.00
2	12	FA	M	2	2	0.00
2	13	FB	F	1	2	0.00
2	13	FB	M	1	2	0.00
2	13	FB	F	2	2	0.00
2	13	FB	M	2	2	0.00
2	14	S	F	1	2	0.00
2	14	S	M	1	2	0.00
2	14	S	F	2	2	0.00
2	14	S	M	2	2	0.00
2	15	FC	F	1	2	0.00
2	15	FC	M	1	2	0.00
2	15	FC	F	2	2	0.00

2	15	FC	M	2	2	0.00
2	16	FA	F	1	2	0.00
2	16	FA	M	1	2	0.00
2	16	FA	F	2	2	0.00
2	16	FA	M	2	2	0.00
