# Supplementary material for: Slow and steady wins the race: The behaviour and welfare of commercial faster growing broiler breeds compared to a commercial slower growing breed
Source: PLoS One. 2020 Apr 6;15(4):e0231006. doi: 10.1371/journal.pone.0231006 (PMC7135253; doi:10.1371/journal.pone.0231006)
Supplement: S7 Data — (PDF) [file pone.0231006.s007.pdf]

Replicate	Pen	Breed	Sex	WA	Breast Cle	Prop
1	1	FB	F	1	0	0.00
1	1	FB	M	1	0	0.00
1	1	FB	F	2	0	0.00
1	1	FB	M	2	0	0.00
1	2	FA	F	1	0	0.00
1	2	FA	M	1	0	0.00
1	2	FA	F	2	0	0.00
1	2	FA	M	2	0	0.00
1	3	FC	F	1	0	0.00
1	3	FC	M	1	0	0.00
1	3	FC	F	2	0	0.00
1	3	FC	M	2	0	0.00
1	4	S	F	1	0	0.17
1	4	S	M	1	0	0.15
1	4	S	F	2	0	0.07
1	4	S	M	2	0	0.10
1	5	FB	F	1	0	0.00
1	5	FB	M	1	0	0.00
1	5	FB	F	2	0	0.00
1	5	FB	M	2	0	0.00
1	6	FA	F	1	0	0.00
1	6	FA	M	1	0	0.00
1	6	FA	F	2	0	0.00
1	6	FA	M	2	0	0.00
1	7	FC	F	1	0	0.00
1	7	FC	M	1	0	0.00
1	7	FC	F	2	0	0.00
1	7	FC	M	2	0	0.00
1	8	S	F	1	0	0.15
1	8	S	M	1	0	0.17
1	8	S	F	2	0	0.06
1	8	S	M	2	0	0.00
1	9	S	F	1	0	0.18
1	9	S	M	1	0	0.29
1	9	S	F	2	0	0.38
1	9	S	M	2	0	0.31
1	10	FB	F	1	0	0.00
1	10	FB	M	1	0	0.00
1	10	FB	F	2	0	0.00
1	10	FB	M	2	0	0.00
1	11	FA	F	1	0	0.00
1	11	FA	M	1	0	0.00
1	11	FA	F	2	0	0.00
1	11	FA	M	2	0	0.00
1	12	FC	F	1	0	0.00
1	12	FC	M	1	0	0.00
1	12	FC	F	2	0	0.00
1	12	FC	M	2	0	0.00
1	13	S	F	1	0	0.50
1	13	S	M	1	0	0.53
1	13	S	F	2	0	0.07
1	13	S	M	2	0	0.09

1	14	FB	F	1	0	0.00
1	14	FB	M	1	0	0.00
1	14	FB	F	2	0	0.00
1	14	FB	M	2	0	0.00
1	15	FA	F	1	0	0.00
1	15	FA	M	1	0	0.00
1	15	FA	F	2	0	0.00
1	15	FA	M	2	0	0.00
1	16	FC	F	1	0	0.00
1	16	FC	M	1	0	0.00
1	16	FC	F	2	0	0.00
1	16	FC	M	2	0	0.00
2	1	FA	F	1	0	0.00
2	1	FA	M	1	0	0.00
2	1	FA	F	2	0	0.00
2	1	FA	M	2	0	0.00
2	2	FB	F	1	0	0.00
2	2	FB	M	1	0	0.00
2	2	FB	F	2	0	0.00
2	2	FB	M	2	0	0.00
2	3	S	F	1	0	0.58
2	3	S	M	1	0	0.31
2	3	S	F	2	0	0.50
2	3	S	M	2	0	0.33
2	4	FC	F	1	0	0.00
2	4	FC	M	1	0	0.00
2	4	FC	F	2	0	0.00
2	4	FC	M	2	0	0.00
2	5	FA	F	1	0	0.00
2	5	FA	M	1	0	0.00
2	5	FA	F	2	0	0.00
2	5	FA	M	2	0	0.00
2	6	FB	F	1	0	0.00
2	6	FB	M	1	0	0.00
2	6	FB	F	2	0	0.00
2	6	FB	M	2	0	0.00
2	7	S	F	1	0	0.58
2	7	S	M	1	0	0.31
2	7	S	F	2	0	0.45
2	7	S	M	2	0	0.36
2	8	FC	F	1	0	0.00
2	8	FC	M	1	0	0.00
2	8	FC	F	2	0	0.00
2	8	FC	M	2	0	0.00
2	9	FB	F	1	0	0.00
2	9	FB	M	1	0	0.00
2	9	FB	F	2	0	0.00
2	9	FB	M	2	0	0.00
2	10	S	F	1	0	0.22
2	10	S	M	1	0	0.38
2	10	S	F	2	0	0.50
2	10	S	M	2	0	0.53
2	11	FC	F	1	0	0.00

2	11	FC	M	1	0	0.00
2	11	FC	F	2	0	0.00
2	11	FC	M	2	0	0.00
2	12	FA	F	1	0	0.00
2	12	FA	M	1	0	0.00
2	12	FA	F	2	0	0.00
2	12	FA	M	2	0	0.00
2	13	FB	F	1	0	0.00
2	13	FB	M	1	0	0.00
2	13	FB	F	2	0	0.00
2	13	FB	M	2	0	0.00
2	14	S	F	1	0	0.22
2	14	S	M	1	0	0.25
2	14	S	F	2	0	0.36
2	14	S	M	2	0	0.64
2	15	FC	F	1	0	0.00
2	15	FC	M	1	0	0.00
2	15	FC	F	2	0	0.00
2	15	FC	M	2	0	0.00
2	16	FA	F	1	0	0.00
2	16	FA	M	1	0	0.00
2	16	FA	F	2	0	0.00
2	16	FA	M	2	0	0.00
1	1	FB	F	1	1	0.08
1	1	FB	M	1	1	0.00
1	1	FB	F	2	1	0.06
1	1	FB	M	2	1	0.00
1	2	FA	F	1	1	0.00
1	2	FA	M	1	1	0.36
1	2	FA	F	2	1	0.00
1	2	FA	M	2	1	0.00
1	3	FC	F	1	1	0.30
1	3	FC	M	1	1	0.40
1	3	FC	F	2	1	0.08
1	3	FC	M	2	1	0.00
1	4	S	F	1	1	0.67
1	4	S	M	1	1	0.38
1	4	S	F	2	1	0.47
1	4	S	M	2	1	0.60
1	5	FB	F	1	1	0.60
1	5	FB	M	1	1	0.13
1	5	FB	F	2	1	0.11
1	5	FB	M	2	1	0.00
1	6	FA	F	1	1	0.36
1	6	FA	M	1	1	0.21
1	6	FA	F	2	1	0.17
1	6	FA	M	2	1	0.00
1	7	FC	F	1	1	0.31
1	7	FC	M	1	1	0.17
1	7	FC	F	2	1	0.07
1	7	FC	M	2	1	0.00
1	8	S	F	1	1	0.31
1	8	S	M	1	1	0.42

1	8	S	F	2	1	0.50
1	8	S	M	2	1	0.33
1	9	S	F	1	1	0.55
1	9	S	M	1	1	0.43
1	9	S	F	2	1	0.38
1	9	S	M	2	1	0.13
1	10	FB	F	1	1	0.25
1	10	FB	M	1	1	0.18
1	10	FB	F	2	1	0.00
1	10	FB	M	2	1	0.00
1	11	FA	F	1	1	0.10
1	11	FA	M	1	1	0.00
1	11	FA	F	2	1	0.00
1	11	FA	M	2	1	0.17
1	12	FC	F	1	1	0.00
1	12	FC	M	1	1	0.07
1	12	FC	F	2	1	0.00
1	12	FC	M	2	1	0.00
1	13	S	F	1	1	0.08
1	13	S	M	1	1	0.00
1	13	S	F	2	1	0.57
1	13	S	M	2	1	0.27
1	14	FB	F	1	1	0.15
1	14	FB	M	1	1	0.08
1	14	FB	F	2	1	0.00
1	14	FB	M	2	1	0.00
1	15	FA	F	1	1	0.00
1	15	FA	M	1	1	0.00
1	15	FA	F	2	1	0.00
1	15	FA	M	2	1	0.00
1	16	FC	F	1	1	0.00
1	16	FC	M	1	1	0.20
1	16	FC	F	2	1	0.09
1	16	FC	M	2	1	0.00
2	1	FA	F	1	1	0.67
2	1	FA	M	1	1	0.36
2	1	FA	F	2	1	0.00
2	1	FA	M	2	1	0.08
2	2	FB	F	1	1	0.55
2	2	FB	M	1	1	0.36
2	2	FB	F	2	1	0.08
2	2	FB	M	2	1	0.08
2	3	S	F	1	1	0.25
2	3	S	M	1	1	0.38
2	3	S	F	2	1	0.40
2	3	S	M	2	1	0.40
2	4	FC	F	1	1	0.70
2	4	FC	M	1	1	0.27
2	4	FC	F	2	1	0.33
2	4	FC	M	2	1	0.08
2	5	FA	F	1	1	0.77
2	5	FA	M	1	1	0.67
2	5	FA	F	2	1	0.15

2	5	FA	M	2	1	0.08
2	6	FB	F	1	1	0.70
2	6	FB	M	1	1	0.13
2	6	FB	F	2	1	0.10
2	6	FB	M	2	1	0.07
2	7	S	F	1	1	0.25
2	7	S	M	1	1	0.69
2	7	S	F	2	1	0.55
2	7	S	M	2	1	0.50
2	8	FC	F	1	1	0.44
2	8	FC	M	1	1	0.22
2	8	FC	F	2	1	0.33
2	8	FC	M	2	1	0.08
2	9	FB	F	1	1	0.46
2	9	FB	M	1	1	0.25
2	9	FB	F	2	1	0.00
2	9	FB	M	2	1	0.08
2	10	S	F	1	1	0.56
2	10	S	M	1	1	0.38
2	10	S	F	2	1	0.50
2	10	S	M	2	1	0.35
2	11	FC	F	1	1	0.31
2	11	FC	M	1	1	0.33
2	11	FC	F	2	1	0.21
2	11	FC	M	2	1	0.09
2	12	FA	F	1	1	0.36
2	12	FA	M	1	1	0.64
2	12	FA	F	2	1	0.17
2	12	FA	M	2	1	0.08
2	13	FB	F	1	1	0.50
2	13	FB	M	1	1	0.31
2	13	FB	F	2	1	0.14
2	13	FB	M	2	1	0.18
2	14	S	F	1	1	0.67
2	14	S	M	1	1	0.44
2	14	S	F	2	1	0.27
2	14	S	M	2	1	0.29
2	15	FC	F	1	1	0.42
2	15	FC	M	1	1	0.15
2	15	FC	F	2	1	0.00
2	15	FC	M	2	1	0.25
2	16	FA	F	1	1	0.42
2	16	FA	M	1	1	0.23
2	16	FA	F	2	1	0.15
2	16	FA	M	2	1	0.17
1	1	FB	F	1	2	0.92
1	1	FB	M	1	2	1.00
1	1	FB	F	2	2	0.94
1	1	FB	M	2	2	1.00
1	2	FA	F	1	2	1.00
1	2	FA	M	1	2	0.64
1	2	FA	F	2	2	1.00
1	2	FA	M	2	2	1.00

1	3	FC	F	1	2	0.70
1	3	FC	M	1	2	0.60
1	3	FC	F	2	2	0.92
1	3	FC	M	2	2	1.00
1	4	S	F	1	2	0.17
1	4	S	M	1	2	0.46
1	4	S	F	2	2	0.47
1	4	S	M	2	2	0.30
1	5	FB	F	1	2	0.40
1	5	FB	M	1	2	0.87
1	5	FB	F	2	2	0.89
1	5	FB	M	2	2	1.00
1	6	FA	F	1	2	0.64
1	6	FA	M	1	2	0.79
1	6	FA	F	2	2	0.83
1	6	FA	M	2	2	1.00
1	7	FC	F	1	2	0.77
1	7	FC	M	1	2	0.83
1	7	FC	F	2	2	0.93
1	7	FC	M	2	2	1.00
1	8	S	F	1	2	0.54
1	8	S	M	1	2	0.42
1	8	S	F	2	2	0.44
1	8	S	M	2	2	0.67
1	9	S	F	1	2	0.27
1	9	S	M	1	2	0.29
1	9	S	F	2	2	0.25
1	9	S	M	2	2	0.63
1	10	FB	F	1	2	0.75
1	10	FB	M	1	2	0.82
1	10	FB	F	2	2	1.00
1	10	FB	M	2	2	0.91
1	11	FA	F	1	2	0.90
1	11	FA	M	1	2	1.00
1	11	FA	F	2	2	1.00
1	11	FA	M	2	2	0.83
1	12	FC	F	1	2	1.00
1	12	FC	M	1	2	0.93
1	12	FC	F	2	2	1.00
1	12	FC	M	2	2	1.00
1	13	S	F	1	2	0.42
1	13	S	M	1	2	0.47
1	13	S	F	2	2	0.36
1	13	S	M	2	2	0.64
1	14	FB	F	1	2	0.85
1	14	FB	M	1	2	0.92
1	14	FB	F	2	2	1.00
1	14	FB	M	2	2	1.00
1	15	FA	F	1	2	1.00
1	15	FA	M	1	2	1.00
1	15	FA	F	2	2	1.00
1	15	FA	M	2	2	1.00
1	16	FC	F	1	2	1.00

1	16	FC	M	1	2	0.70
1	16	FC	F	2	2	0.91
1	16	FC	M	2	2	1.00
2	1	FA	F	1	2	0.33
2	1	FA	M	1	2	0.64
2	1	FA	F	2	2	1.00
2	1	FA	M	2	2	0.92
2	2	FB	F	1	2	0.45
2	2	FB	M	1	2	0.64
2	2	FB	F	2	2	0.92
2	2	FB	M	2	2	0.92
2	3	S	F	1	2	0.17
2	3	S	M	1	2	0.31
2	3	S	F	2	2	0.10
2	3	S	M	2	2	0.27
2	4	FC	F	1	2	0.30
2	4	FC	M	1	2	0.73
2	4	FC	F	2	2	0.67
2	4	FC	M	2	2	0.92
2	5	FA	F	1	2	0.23
2	5	FA	M	1	2	0.33
2	5	FA	F	2	2	0.85
2	5	FA	M	2	2	0.92
2	6	FB	F	1	2	0.30
2	6	FB	M	1	2	0.87
2	6	FB	F	2	2	0.90
2	6	FB	M	2	2	0.93
2	7	S	F	1	2	0.17
2	7	S	M	1	2	0.00
2	7	S	F	2	2	0.00
2	7	S	M	2	2	0.14
2	8	FC	F	1	2	0.56
2	8	FC	M	1	2	0.78
2	8	FC	F	2	2	0.67
2	8	FC	M	2	2	0.92
2	9	FB	F	1	2	0.54
2	9	FB	M	1	2	0.75
2	9	FB	F	2	2	1.00
2	9	FB	M	2	2	0.92
2	10	S	F	1	2	0.22
2	10	S	M	1	2	0.25
2	10	S	F	2	2	0.00
2	10	S	M	2	2	0.12
2	11	FC	F	1	2	0.69
2	11	FC	M	1	2	0.67
2	11	FC	F	2	2	0.79
2	11	FC	M	2	2	0.91
2	12	FA	F	1	2	0.64
2	12	FA	M	1	2	0.36
2	12	FA	F	2	2	0.83
2	12	FA	M	2	2	0.92
2	13	FB	F	1	2	0.50
2	13	FB	M	1	2	0.69

2	13	FB	F	2	2	0.86
2	13	FB	M	2	2	0.82
2	14	S	F	1	2	0.11
2	14	S	M	1	2	0.31
2	14	S	F	2	2	0.36
2	14	S	M	2	2	0.07
2	15	FC	F	1	2	0.58
2	15	FC	M	1	2	0.85
2	15	FC	F	2	2	1.00
2	15	FC	M	2	2	0.75
2	16	FA	F	1	2	0.58
2	16	FA	M	1	2	0.77
2	16	FA	F	2	2	0.85
2	16	FA	M	2	2	0.83
