# Supplementary material for: Slow and steady wins the race: The behaviour and welfare of commercial faster growing broiler breeds compared to a commercial slower growing breed
Source: PLoS One. 2020 Apr 6;15(4):e0231006. doi: 10.1371/journal.pone.0231006 (PMC7135253; doi:10.1371/journal.pone.0231006)
Supplement: S8 Data — (PDF) [file pone.0231006.s008.pdf]

Replicate	Pen	Breed	Sex	WA	Podo Scor	Prop
1	1	FB	F	1	0	0.88
1	1	FB	M	1	0	0.91
1	1	FB	F	2	0	1.00
1	1	FB	M	2	0	1.00
1	2	FA	F	1	0	0.89
1	2	FA	M	1	0	0.86
1	2	FA	F	2	0	1.00
1	2	FA	M	2	0	1.00
1	3	FC	F	1	0	0.95
1	3	FC	M	1	0	1.00
1	3	FC	F	2	0	1.00
1	3	FC	M	2	0	1.00
1	4	S	F	1	0	1.00
1	4	S	M	1	0	1.00
1	4	S	F	2	0	1.00
1	4	S	M	2	0	1.00
1	5	FB	F	1	0	0.95
1	5	FB	M	1	0	0.93
1	5	FB	F	2	0	1.00
1	5	FB	M	2	0	1.00
1	6	FA	F	1	0	1.00
1	6	FA	M	1	0	1.00
1	6	FA	F	2	0	1.00
1	6	FA	M	2	0	1.00
1	7	FC	F	1	0	1.00
1	7	FC	M	1	0	0.88
1	7	FC	F	2	0	0.96
1	7	FC	M	2	0	1.00
1	8	S	F	1	0	1.00
1	8	S	M	1	0	0.96
1	8	S	F	2	0	0.91
1	8	S	M	2	0	1.00
1	9	S	F	1	0	1.00
1	9	S	M	1	0	1.00
1	9	S	F	2	0	1.00
1	9	S	M	2	0	1.03
1	10	FB	F	1	0	1.00
1	10	FB	M	1	0	1.00
1	10	FB	F	2	0	1.00
1	10	FB	M	2	0	0.91
1	11	FA	F	1	0	1.00
1	11	FA	M	1	0	1.00
1	11	FA	F	2	0	1.00
1	11	FA	M	2	0	1.00
1	12	FC	F	1	0	0.95
1	12	FC	M	1	0	1.00
1	12	FC	F	2	0	0.96
1	12	FC	M	2	0	0.95
1	13	S	F	1	0	1.00
1	13	S	M	1	0	1.00
1	13	S	F	2	0	1.00
1	13	S	M	2	0	1.00

1	14	FB	F	1	0	1.00
1	14	FB	M	1	0	0.96
1	14	FB	F	2	0	1.00
1	14	FB	M	2	0	1.00
1	15	FA	F	1	0	1.00
1	15	FA	M	1	0	1.00
1	15	FA	F	2	0	1.00
1	15	FA	M	2	0	1.00
1	16	FC	F	1	0	0.90
1	16	FC	M	1	0	0.80
1	16	FC	F	2	0	1.00
1	16	FC	M	2	0	0.96
2	1	FA	F	1	0	0.92
2	1	FA	M	1	0	1.00
2	1	FA	F	2	0	1.00
2	1	FA	M	2	0	1.00
2	2	FB	F	1	0	1.00
2	2	FB	M	1	0	1.00
2	2	FB	F	2	0	1.00
2	2	FB	M	2	0	1.00
2	3	S	F	1	0	1.00
2	3	S	M	1	0	0.96
2	3	S	F	2	0	0.90
2	3	S	M	2	0	1.00
2	4	FC	F	1	0	1.00
2	4	FC	M	1	0	1.00
2	4	FC	F	2	0	1.00
2	4	FC	M	2	0	1.00
2	5	FA	F	1	0	0.92
2	5	FA	M	1	0	1.00
2	5	FA	F	2	0	1.00
2	5	FA	M	2	0	0.96
2	6	FB	F	1	0	1.00
2	6	FB	M	1	0	1.00
2	6	FB	F	2	0	1.00
2	6	FB	M	2	0	1.00
2	7	S	F	1	0	1.00
2	7	S	M	1	0	1.00
2	7	S	F	2	0	1.00
2	7	S	M	2	0	1.00
2	8	FC	F	1	0	1.00
2	8	FC	M	1	0	0.78
2	8	FC	F	2	0	1.00
2	8	FC	M	2	0	0.92
2	9	FB	F	1	0	0.96
2	9	FB	M	1	0	1.00
2	9	FB	F	2	0	1.00
2	9	FB	M	2	0	0.96
2	10	S	F	1	0	0.78
2	10	S	M	1	0	1.00
2	10	S	F	2	0	0.94
2	10	S	M	2	0	1.00
2	11	FC	F	1	0	1.00

2	11	FC	M	1	0	1.00
2	11	FC	F	2	0	1.00
2	11	FC	M	2	0	0.95
2	12	FA	F	1	0	1.00
2	12	FA	M	1	0	1.00
2	12	FA	F	2	0	1.00
2	12	FA	M	2	0	1.00
2	13	FB	F	1	0	0.92
2	13	FB	M	1	0	0.92
2	13	FB	F	2	0	1.00
2	13	FB	M	2	0	0.91
2	14	S	F	1	0	1.00
2	14	S	M	1	0	1.00
2	14	S	F	2	0	1.00
2	14	S	M	2	0	1.00
2	15	FC	F	1	0	0.88
2	15	FC	M	1	0	0.96
2	15	FC	F	2	0	0.89
2	15	FC	M	2	0	0.91
2	16	FA	F	1	0	1.00
2	16	FA	M	1	0	1.00
2	16	FA	F	2	0	1.00
2	16	FA	M	2	0	1.00
1	1	FB	F	1	0P	0.12
1	1	FB	M	1	0P	0.09
1	1	FB	F	2	0P	0.00
1	1	FB	M	2	0P	0.00
1	2	FA	F	1	0P	0.11
1	2	FA	M	1	0P	0.14
1	2	FA	F	2	0P	0.00
1	2	FA	M	2	0P	0.00
1	3	FC	F	1	0P	0.05
1	3	FC	M	1	0P	0.00
1	3	FC	F	2	0P	0.00
1	3	FC	M	2	0P	0.00
1	4	S	F	1	0P	0.00
1	4	S	M	1	0P	0.00
1	4	S	F	2	0P	0.00
1	4	S	M	2	0P	0.00
1	5	FB	F	1	0P	0.05
1	5	FB	M	1	0P	0.07
1	5	FB	F	2	0P	0.00
1	5	FB	M	2	0P	0.00
1	6	FA	F	1	0P	0.00
1	6	FA	M	1	0P	0.00
1	6	FA	F	2	0P	0.00
1	6	FA	M	2	0P	0.00
1	7	FC	F	1	0P	0.04
1	7	FC	M	1	0P	0.13
1	7	FC	F	2	0P	0.04
1	7	FC	M	2	0P	0.00
1	8	S	F	1	0P	0.00
1	8	S	M	1	0P	0.04

1	8	S	F	2	0P	0.00
1	8	S	M	2	0P	0.00
1	9	S	F	1	0P	0.00
1	9	S	M	1	0P	0.00
1	9	S	F	2	0P	0.00
1	9	S	M	2	0P	0.00
1	10	FB	F	1	0P	0.00
1	10	FB	M	1	0P	0.00
1	10	FB	F	2	0P	0.00
1	10	FB	M	2	0P	0.05
1	11	FA	F	1	0P	0.00
1	11	FA	M	1	0P	0.00
1	11	FA	F	2	0P	0.00
1	11	FA	M	2	0P	0.00
1	12	FC	F	1	0P	0.05
1	12	FC	M	1	0P	0.00
1	12	FC	F	2	0P	0.04
1	12	FC	M	2	0P	0.05
1	13	S	F	1	0P	0.00
1	13	S	M	1	0P	0.00
1	13	S	F	2	0P	0.00
1	13	S	M	2	0P	0.00
1	14	FB	F	1	0P	0.00
1	14	FB	M	1	0P	0.04
1	14	FB	F	2	0P	0.00
1	14	FB	M	2	0P	0.00
1	15	FA	F	1	0P	0.00
1	15	FA	M	1	0P	0.00
1	15	FA	F	2	0P	0.00
1	15	FA	M	2	0P	0.00
1	16	FC	F	1	0P	0.10
1	16	FC	M	1	0P	0.10
1	16	FC	F	2	0P	0.00
1	16	FC	M	2	0P	0.04
2	1	FA	F	1	0P	0.08
2	1	FA	M	1	0P	0.00
2	1	FA	F	2	0P	0.00
2	1	FA	M	2	0P	0.00
2	2	FB	F	1	0P	0.00
2	2	FB	M	1	0P	0.00
2	2	FB	F	2	0P	0.00
2	2	FB	M	2	0P	0.00
2	3	S	F	1	0P	0.00
2	3	S	M	1	0P	0.04
2	3	S	F	2	0P	0.10
2	3	S	M	2	0P	0.00
2	4	FC	F	1	0P	0.00
2	4	FC	M	1	0P	0.00
2	4	FC	F	2	0P	0.00
2	4	FC	M	2	0P	0.00
2	5	FA	F	1	0P	0.08
2	5	FA	M	1	0P	0.00
2	5	FA	F	2	0P	0.00

2	5	FA	M	2	0P	0.04
2	6	FB	F	1	0P	0.00
2	6	FB	M	1	0P	0.00
2	6	FB	F	2	0P	0.00
2	6	FB	M	2	0P	0.00
2	7	S	F	1	0P	0.00
2	7	S	M	1	0P	0.00
2	7	S	F	2	0P	0.00
2	7	S	M	2	0P	0.00
2	8	FC	F	1	0P	0.00
2	8	FC	M	1	0P	0.22
2	8	FC	F	2	0P	0.00
2	8	FC	M	2	0P	0.08
2	9	FB	F	1	0P	0.04
2	9	FB	M	1	0P	0.00
2	9	FB	F	2	0P	0.00
2	9	FB	M	2	0P	0.04
2	10	S	F	1	0P	0.22
2	10	S	M	1	0P	0.00
2	10	S	F	2	0P	0.06
2	10	S	M	2	0P	0.00
2	11	FC	F	1	0P	0.00
2	11	FC	M	1	0P	0.00
2	11	FC	F	2	0P	0.00
2	11	FC	M	2	0P	0.05
2	12	FA	F	1	0P	0.00
2	12	FA	M	1	0P	0.00
2	12	FA	F	2	0P	0.00
2	12	FA	M	2	0P	0.00
2	13	FB	F	1	0P	0.08
2	13	FB	M	1	0P	0.08
2	13	FB	F	2	0P	0.00
2	13	FB	M	2	0P	0.09
2	14	S	F	1	0P	0.00
2	14	S	M	1	0P	0.00
2	14	S	F	2	0P	0.00
2	14	S	M	2	0P	0.00
2	15	FC	F	1	0P	0.13
2	15	FC	M	1	0P	0.04
2	15	FC	F	2	0P	0.11
2	15	FC	M	2	0P	0.03
2	16	FA	F	1	0P	0.00
2	16	FA	M	1	0P	0.00
2	16	FA	F	2	0P	0.00
2	16	FA	M	2	0P	0.00
1	1	FB	F	1	1	0.00
1	1	FB	M	1	1	0.00
1	1	FB	F	2	1	0.00
1	1	FB	M	2	1	0.00
1	2	FA	F	1	1	0.00
1	2	FA	M	1	1	0.00
1	2	FA	F	2	1	0.00
1	2	FA	M	2	1	0.00

1	3	FC	F	1	1	0.00
1	3	FC	M	1	1	0.00
1	3	FC	F	2	1	0.00
1	3	FC	M	2	1	0.00
1	4	S	F	1	1	0.00
1	4	S	M	1	1	0.00
1	4	S	F	2	1	0.00
1	4	S	M	2	1	0.00
1	5	FB	F	1	1	0.00
1	5	FB	M	1	1	0.00
1	5	FB	F	2	1	0.00
1	5	FB	M	2	1	0.00
1	6	FA	F	1	1	0.00
1	6	FA	M	1	1	0.00
1	6	FA	F	2	1	0.00
1	6	FA	M	2	1	0.00
1	7	FC	F	1	1	0.00
1	7	FC	M	1	1	0.00
1	7	FC	F	2	1	0.00
1	7	FC	M	2	1	0.00
1	8	S	F	1	1	0.00
1	8	S	M	1	1	0.00
1	8	S	F	2	1	0.09
1	8	S	M	2	1	0.00
1	9	S	F	1	1	0.00
1	9	S	M	1	1	0.00
1	9	S	F	2	1	0.00
1	9	S	M	2	1	0.00
1	10	FB	F	1	1	0.00
1	10	FB	M	1	1	0.00
1	10	FB	F	2	1	0.00
1	10	FB	M	2	1	0.00
1	11	FA	F	1	1	0.00
1	11	FA	M	1	1	0.00
1	11	FA	F	2	1	0.00
1	11	FA	M	2	1	0.00
1	12	FC	F	1	1	0.00
1	12	FC	M	1	1	0.00
1	12	FC	F	2	1	0.00
1	12	FC	M	2	1	0.00
1	13	S	F	1	1	0.00
1	13	S	M	1	1	0.00
1	13	S	F	2	1	0.00
1	13	S	M	2	1	0.00
1	14	FB	F	1	1	0.00
1	14	FB	M	1	1	0.00
1	14	FB	F	2	1	0.00
1	14	FB	M	2	1	0.00
1	15	FA	F	1	1	0.00
1	15	FA	M	1	1	0.00
1	15	FA	F	2	1	0.00
1	15	FA	M	2	1	0.00
1	16	FC	F	1	1	0.00

1	16	FC	M	1	1	0.00
1	16	FC	F	2	1	0.00
1	16	FC	M	2	1	0.00
2	1	FA	F	1	1	0.00
2	1	FA	M	1	1	0.00
2	1	FA	F	2	1	0.00
2	1	FA	M	2	1	0.00
2	2	FB	F	1	1	0.00
2	2	FB	M	1	1	0.00
2	2	FB	F	2	1	0.00
2	2	FB	M	2	1	0.00
2	3	S	F	1	1	0.00
2	3	S	M	1	1	0.00
2	3	S	F	2	1	0.00
2	3	S	M	2	1	0.00
2	4	FC	F	1	1	0.00
2	4	FC	M	1	1	0.00
2	4	FC	F	2	1	0.00
2	4	FC	M	2	1	0.00
2	5	FA	F	1	1	0.00
2	5	FA	M	1	1	0.00
2	5	FA	F	2	1	0.00
2	5	FA	M	2	1	0.00
2	6	FB	F	1	1	0.00
2	6	FB	M	1	1	0.00
2	6	FB	F	2	1	0.00
2	6	FB	M	2	1	0.00
2	7	S	F	1	1	0.00
2	7	S	M	1	1	0.00
2	7	S	F	2	1	0.00
2	7	S	M	2	1	0.00
2	8	FC	F	1	1	0.00
2	8	FC	M	1	1	0.00
2	8	FC	F	2	1	0.00
2	8	FC	M	2	1	0.00
2	9	FB	F	1	1	0.00
2	9	FB	M	1	1	0.00
2	9	FB	F	2	1	0.00
2	9	FB	M	2	1	0.00
2	10	S	F	1	1	0.00
2	10	S	M	1	1	0.00
2	10	S	F	2	1	0.00
2	10	S	M	2	1	0.00
2	11	FC	F	1	1	0.00
2	11	FC	M	1	1	0.00
2	11	FC	F	2	1	0.00
2	11	FC	M	2	1	0.00
2	12	FA	F	1	1	0.00
2	12	FA	M	1	1	0.00
2	12	FA	F	2	1	0.00
2	12	FA	M	2	1	0.00
2	13	FB	F	1	1	0.00
2	13	FB	M	1	1	0.00

2	13	FB	F	2	1	0.00
2	13	FB	M	2	1	0.00
2	14	S	F	1	1	0.00
2	14	S	M	1	1	0.00
2	14	S	F	2	1	0.00
2	14	S	M	2	1	0.00
2	15	FC	F	1	1	0.00
2	15	FC	M	1	1	0.00
2	15	FC	F	2	1	0.00
2	15	FC	M	2	1	0.06
2	16	FA	F	1	1	0.00
2	16	FA	M	1	1	0.00
2	16	FA	F	2	1	0.00
2	16	FA	M	2	1	0.00
1	1	FB	F	1	2	0.00
1	1	FB	M	1	2	0.00
1	1	FB	F	2	2	0.00
1	1	FB	M	2	2	0.00
1	2	FA	F	1	2	0.00
1	2	FA	M	1	2	0.00
1	2	FA	F	2	2	0.00
1	2	FA	M	2	2	0.00
1	3	FC	F	1	2	0.00
1	3	FC	M	1	2	0.00
1	3	FC	F	2	2	0.00
1	3	FC	M	2	2	0.00
1	4	S	F	1	2	0.00
1	4	S	M	1	2	0.00
1	4	S	F	2	2	0.00
1	4	S	M	2	2	0.00
1	5	FB	F	1	2	0.00
1	5	FB	M	1	2	0.00
1	5	FB	F	2	2	0.00
1	5	FB	M	2	2	0.00
1	6	FA	F	1	2	0.00
1	6	FA	M	1	2	0.00
1	6	FA	F	2	2	0.00
1	6	FA	M	2	2	0.00
1	7	FC	F	1	2	0.04
1	7	FC	M	1	2	0.00
1	7	FC	F	2	2	0.00
1	7	FC	M	2	2	0.00
1	8	S	F	1	2	0.00
1	8	S	M	1	2	0.00
1	8	S	F	2	2	0.00
1	8	S	M	2	2	0.00
1	9	S	F	1	2	0.00
1	9	S	M	1	2	0.00
1	9	S	F	2	2	0.00
1	9	S	M	2	2	0.03
1	10	FB	F	1	2	0.00
1	10	FB	M	1	2	0.00
1	10	FB	F	2	2	0.00

1	10	FB	M	2	2	0.05
1	11	FA	F	1	2	0.00
1	11	FA	M	1	2	0.00
1	11	FA	F	2	2	0.00
1	11	FA	M	2	2	0.00
1	12	FC	F	1	2	0.00
1	12	FC	M	1	2	0.00
1	12	FC	F	2	2	0.00
1	12	FC	M	2	2	0.00
1	13	S	F	1	2	0.00
1	13	S	M	1	2	0.00
1	13	S	F	2	2	0.00
1	13	S	M	2	2	0.00
1	14	FB	F	1	2	0.00
1	14	FB	M	1	2	0.00
1	14	FB	F	2	2	0.00
1	14	FB	M	2	2	0.00
1	15	FA	F	1	2	0.00
1	15	FA	M	1	2	0.00
1	15	FA	F	2	2	0.00
1	15	FA	M	2	2	0.00
1	16	FC	F	1	2	0.00
1	16	FC	M	1	2	0.00
1	16	FC	F	2	2	0.00
1	16	FC	M	2	2	0.00
2	1	FA	F	1	2	0.00
2	1	FA	M	1	2	0.00
2	1	FA	F	2	2	0.00
2	1	FA	M	2	2	0.00
2	2	FB	F	1	2	0.00
2	2	FB	M	1	2	0.00
2	2	FB	F	2	2	0.00
2	2	FB	M	2	2	0.00
2	3	S	F	1	2	0.00
2	3	S	M	1	2	0.00
2	3	S	F	2	2	0.00
2	3	S	M	2	2	0.00
2	4	FC	F	1	2	0.00
2	4	FC	M	1	2	0.00
2	4	FC	F	2	2	0.00
2	4	FC	M	2	2	0.00
2	5	FA	F	1	2	0.00
2	5	FA	M	1	2	0.00
2	5	FA	F	2	2	0.00
2	5	FA	M	2	2	0.00
2	6	FB	F	1	2	0.00
2	6	FB	M	1	2	0.00
2	6	FB	F	2	2	0.00
2	6	FB	M	2	2	0.00
2	7	S	F	1	2	0.00
2	7	S	M	1	2	0.00
2	7	S	F	2	2	0.00
2	7	S	M	2	2	0.00

2	8	FC	F	1	2	0.00
2	8	FC	M	1	2	0.00
2	8	FC	F	2	2	0.00
2	8	FC	M	2	2	0.00
2	9	FB	F	1	2	0.00
2	9	FB	M	1	2	0.00
2	9	FB	F	2	2	0.00
2	9	FB	M	2	2	0.00
2	10	S	F	1	2	0.00
2	10	S	M	1	2	0.00
2	10	S	F	2	2	0.00
2	10	S	M	2	2	0.00
2	11	FC	F	1	2	0.00
2	11	FC	M	1	2	0.00
2	11	FC	F	2	2	0.00
2	11	FC	M	2	2	0.00
2	12	FA	F	1	2	0.00
2	12	FA	M	1	2	0.00
2	12	FA	F	2	2	0.00
2	12	FA	M	2	2	0.00
2	13	FB	F	1	2	0.00
2	13	FB	M	1	2	0.00
2	13	FB	F	2	2	0.00
2	13	FB	M	2	2	0.00
2	14	S	F	1	2	0.00
2	14	S	M	1	2	0.00
2	14	S	F	2	2	0.00
2	14	S	M	2	2	0.00
2	15	FC	F	1	2	0.00
2	15	FC	M	1	2	0.00
2	15	FC	F	2	2	0.00
2	15	FC	M	2	2	0.00
2	16	FA	F	1	2	0.00
2	16	FA	M	1	2	0.00
2	16	FA	F	2	2	0.00
2	16	FA	M	2	2	0.00
