# Supplementary material for: Slow and steady wins the race: The behaviour and welfare of commercial faster growing broiler breeds compared to a commercial slower growing breed
Source: PLoS One. 2020 Apr 6;15(4):e0231006. doi: 10.1371/journal.pone.0231006 (PMC7135253; doi:10.1371/journal.pone.0231006)
Supplement: S9 Data — (PDF) [file pone.0231006.s009.pdf]

Replicate	Pen	Breed	Sex	WA	Hock Burn	Prop
1	1	FB	F	1	0	0.38
1	1	FB	M	1	0	0.14
1	1	FB	F	2	0	0.19
1	1	FB	M	2	0	0.06
1	2	FA	F	1	0	0.18
1	2	FA	M	1	0	0.18
1	2	FA	F	2	0	0.14
1	2	FA	M	2	0	0.00
1	3	FC	F	1	0	0.30
1	3	FC	M	1	0	0.27
1	3	FC	F	2	0	0.19
1	3	FC	M	2	0	0.17
1	4	S	F	1	0	0.79
1	4	S	M	1	0	0.77
1	4	S	F	2	0	0.80
1	4	S	M	2	0	0.80
1	5	FB	F	1	0	0.35
1	5	FB	M	1	0	0.30
1	5	FB	F	2	0	0.61
1	5	FB	M	2	0	0.16
1	6	FA	F	1	0	0.23
1	6	FA	M	1	0	0.21
1	6	FA	F	2	0	0.25
1	6	FA	M	2	0	0.17
1	7	FC	F	1	0	0.50
1	7	FC	M	1	0	0.42
1	7	FC	F	2	0	0.36
1	7	FC	M	2	0	0.18
1	8	S	F	1	0	0.92
1	8	S	M	1	0	0.50
1	8	S	F	2	0	0.81
1	8	S	M	2	0	0.50
1	9	S	F	1	0	0.91
1	9	S	M	1	0	0.64
1	9	S	F	2	0	1.00
1	9	S	M	2	0	0.66
1	10	FB	F	1	0	0.41
1	10	FB	M	1	0	0.00
1	10	FB	F	2	0	0.29
1	10	FB	M	2	0	0.09
1	11	FA	F	1	0	0.55
1	11	FA	M	1	0	0.40
1	11	FA	F	2	0	0.31
1	11	FA	M	2	0	0.25
1	12	FC	F	1	0	0.55
1	12	FC	M	1	0	0.23
1	12	FC	F	2	0	0.46
1	12	FC	M	2	0	0.14
1	13	S	F	1	0	0.83
1	13	S	M	1	0	0.70
1	13	S	F	2	0	0.79
1	13	S	M	2	0	0.73

1	14	FB	F	1	0	0.23
1	14	FB	M	1	0	0.13
1	14	FB	F	2	0	0.25
1	14	FB	M	2	0	0.03
1	15	FA	F	1	0	0.33
1	15	FA	M	1	0	0.23
1	15	FA	F	2	0	0.41
1	15	FA	M	2	0	0.04
1	16	FC	F	1	0	0.33
1	16	FC	M	1	0	0.45
1	16	FC	F	2	0	0.09
1	16	FC	M	2	0	0.08
2	1	FA	F	1	0	0.33
2	1	FA	M	1	0	0.18
2	1	FA	F	2	0	0.08
2	1	FA	M	2	0	0.00
2	2	FB	F	1	0	0.77
2	2	FB	M	1	0	0.43
2	2	FB	F	2	0	0.46
2	2	FB	M	2	0	0.08
2	3	S	F	1	0	1.00
2	3	S	M	1	0	0.92
2	3	S	F	2	0	0.95
2	3	S	M	2	0	0.83
2	4	FC	F	1	0	1.00
2	4	FC	M	1	0	0.33
2	4	FC	F	2	0	0.58
2	4	FC	M	2	0	0.23
2	5	FA	F	1	0	0.42
2	5	FA	M	1	0	0.42
2	5	FA	F	2	0	0.23
2	5	FA	M	2	0	0.08
2	6	FB	F	1	0	0.75
2	6	FB	M	1	0	0.37
2	6	FB	F	2	0	0.35
2	6	FB	M	2	0	0.30
2	7	S	F	1	0	1.00
2	7	S	M	1	0	0.81
2	7	S	F	2	0	1.00
2	7	S	M	2	0	0.71
2	8	FC	F	1	0	0.50
2	8	FC	M	1	0	0.56
2	8	FC	F	2	0	0.50
2	8	FC	M	2	0	0.42
2	9	FB	F	1	0	0.35
2	9	FB	M	1	0	0.25
2	9	FB	F	2	0	0.31
2	9	FB	M	2	0	0.21
2	10	S	F	1	0	0.89
2	10	S	M	1	0	0.69
2	10	S	F	2	0	0.88
2	10	S	M	2	0	0.68
2	11	FC	F	1	0	0.69

2	11	FC	M	1	0	0.83
2	11	FC	F	2	0	0.39
2	11	FC	M	2	0	0.45
2	12	FA	F	1	0	0.18
2	12	FA	M	1	0	0.27
2	12	FA	F	2	0	0.29
2	12	FA	M	2	0	0.27
2	13	FB	F	1	0	0.42
2	13	FB	M	1	0	0.38
2	13	FB	F	2	0	0.39
2	13	FB	M	2	0	0.27
2	14	S	F	1	0	1.00
2	14	S	M	1	0	0.75
2	14	S	F	2	0	0.91
2	14	S	M	2	0	0.82
2	15	FC	F	1	0	0.63
2	15	FC	M	1	0	0.42
2	15	FC	F	2	0	0.56
2	15	FC	M	2	0	0.19
2	16	FA	F	1	0	0.46
2	16	FA	M	1	0	0.19
2	16	FA	F	2	0	0.08
2	16	FA	M	2	0	0.17
1	1	FB	F	1	0P	0.62
1	1	FB	M	1	0P	0.86
1	1	FB	F	2	0P	0.63
1	1	FB	M	2	0P	0.83
1	2	FA	F	1	0P	0.82
1	2	FA	M	1	0P	0.82
1	2	FA	F	2	0P	0.59
1	2	FA	M	2	0P	0.93
1	3	FC	F	1	0P	0.70
1	3	FC	M	1	0P	0.73
1	3	FC	F	2	0P	0.73
1	3	FC	M	2	0P	0.75
1	4	S	F	1	0P	0.17
1	4	S	M	1	0P	0.19
1	4	S	F	2	0P	0.17
1	4	S	M	2	0P	0.20
1	5	FB	F	1	0P	0.65
1	5	FB	M	1	0P	0.70
1	5	FB	F	2	0P	0.39
1	5	FB	M	2	0P	0.78
1	6	FA	F	1	0P	0.77
1	6	FA	M	1	0P	0.79
1	6	FA	F	2	0P	0.67
1	6	FA	M	2	0P	0.71
1	7	FC	F	1	0P	0.58
1	7	FC	M	1	0P	0.58
1	7	FC	F	2	0P	0.61
1	7	FC	M	2	0P	0.55
1	8	S	F	1	0P	0.00
1	8	S	M	1	0P	0.50

1	8	S	F	2	0P	0.19
1	8	S	M	2	0P	0.28
1	9	S	F	1	0P	0.09
1	9	S	M	1	0P	0.32
1	9	S	F	2	0P	0.00
1	9	S	M	2	0P	0.38
1	10	FB	F	1	0P	0.53
1	10	FB	M	1	0P	0.82
1	10	FB	F	2	0P	0.50
1	10	FB	M	2	0P	0.36
1	11	FA	F	1	0P	0.45
1	11	FA	M	1	0P	0.60
1	11	FA	F	2	0P	0.65
1	11	FA	M	2	0P	0.63
1	12	FC	F	1	0P	0.45
1	12	FC	M	1	0P	0.77
1	12	FC	F	2	0P	0.54
1	12	FC	M	2	0P	0.59
1	13	S	F	1	0P	0.13
1	13	S	M	1	0P	0.27
1	13	S	F	2	0P	0.18
1	13	S	M	2	0P	0.14
1	14	FB	F	1	0P	0.77
1	14	FB	M	1	0P	0.63
1	14	FB	F	2	0P	0.65
1	14	FB	M	2	0P	0.59
1	15	FA	F	1	0P	0.67
1	15	FA	M	1	0P	0.77
1	15	FA	F	2	0P	0.59
1	15	FA	M	2	0P	0.71
1	16	FC	F	1	0P	0.67
1	16	FC	M	1	0P	0.45
1	16	FC	F	2	0P	0.59
1	16	FC	M	2	0P	0.33
2	1	FA	F	1	0P	0.63
2	1	FA	M	1	0P	0.71
2	1	FA	F	2	0P	0.58
2	1	FA	M	2	0P	0.54
2	2	FB	F	1	0P	0.23
2	2	FB	M	1	0P	0.57
2	2	FB	F	2	0P	0.46
2	2	FB	M	2	0P	0.73
2	3	S	F	1	0P	0.00
2	3	S	M	1	0P	0.08
2	3	S	F	2	0P	0.05
2	3	S	M	2	0P	0.17
2	4	FC	F	1	0P	0.00
2	4	FC	M	1	0P	0.67
2	4	FC	F	2	0P	0.38
2	4	FC	M	2	0P	0.54
2	5	FA	F	1	0P	0.42
2	5	FA	M	1	0P	0.58
2	5	FA	F	2	0P	0.73

2	5	FA	M	2	0P	0.79
2	6	FB	F	1	0P	0.20
2	6	FB	M	1	0P	0.63
2	6	FB	F	2	0P	0.60
2	6	FB	M	2	0P	0.70
2	7	S	F	1	0P	0.00
2	7	S	M	1	0P	0.19
2	7	S	F	2	0P	0.00
2	7	S	M	2	0P	0.29
2	8	FC	F	1	0P	0.50
2	8	FC	M	1	0P	0.44
2	8	FC	F	2	0P	0.50
2	8	FC	M	2	0P	0.54
2	9	FB	F	1	0P	0.58
2	9	FB	M	1	0P	0.71
2	9	FB	F	2	0P	0.69
2	9	FB	M	2	0P	0.54
2	10	S	F	1	0P	0.11
2	10	S	M	1	0P	0.25
2	10	S	F	2	0P	0.06
2	10	S	M	2	0P	0.26
2	11	FC	F	1	0P	0.23
2	11	FC	M	1	0P	0.17
2	11	FC	F	2	0P	0.39
2	11	FC	M	2	0P	0.41
2	12	FA	F	1	0P	0.82
2	12	FA	M	1	0P	0.64
2	12	FA	F	2	0P	0.63
2	12	FA	M	2	0P	0.65
2	13	FB	F	1	0P	0.54
2	13	FB	M	1	0P	0.62
2	13	FB	F	2	0P	0.43
2	13	FB	M	2	0P	0.41
2	14	S	F	1	0P	0.00
2	14	S	M	1	0P	0.25
2	14	S	F	2	0P	0.09
2	14	S	M	2	0P	0.18
2	15	FC	F	1	0P	0.25
2	15	FC	M	1	0P	0.54
2	15	FC	F	2	0P	0.33
2	15	FC	M	2	0P	0.63
2	16	FA	F	1	0P	0.54
2	16	FA	M	1	0P	0.81
2	16	FA	F	2	0P	0.85
2	16	FA	M	2	0P	0.67
1	1	FB	F	1	1	0.00
1	1	FB	M	1	1	0.00
1	1	FB	F	2	1	0.19
1	1	FB	M	2	1	0.11
1	2	FA	F	1	1	0.00
1	2	FA	M	1	1	0.00
1	2	FA	F	2	1	0.27
1	2	FA	M	2	1	0.07

1	3	FC	F	1	1	0.00
1	3	FC	M	1	1	0.00
1	3	FC	F	2	1	0.08
1	3	FC	M	2	1	0.08
1	4	S	F	1	1	0.04
1	4	S	M	1	1	0.04
1	4	S	F	2	1	0.03
1	4	S	M	2	1	0.00
1	5	FB	F	1	1	0.00
1	5	FB	M	1	1	0.00
1	5	FB	F	2	1	0.00
1	5	FB	M	2	1	0.06
1	6	FA	F	1	1	0.00
1	6	FA	M	1	1	0.00
1	6	FA	F	2	1	0.08
1	6	FA	M	2	1	0.13
1	7	FC	F	1	1	0.00
1	7	FC	M	1	1	0.00
1	7	FC	F	2	1	0.00
1	7	FC	M	2	1	0.23
1	8	S	F	1	1	0.08
1	8	S	M	1	1	0.00
1	8	S	F	2	1	0.00
1	8	S	M	2	1	0.22
1	9	S	F	1	1	0.00
1	9	S	M	1	1	0.04
1	9	S	F	2	1	0.00
1	9	S	M	2	1	0.03
1	10	FB	F	1	1	0.06
1	10	FB	M	1	1	0.18
1	10	FB	F	2	1	0.21
1	10	FB	M	2	1	0.55
1	11	FA	F	1	1	0.00
1	11	FA	M	1	1	0.00
1	11	FA	F	2	1	0.04
1	11	FA	M	2	1	0.13
1	12	FC	F	1	1	0.00
1	12	FC	M	1	1	0.00
1	12	FC	F	2	1	0.00
1	12	FC	M	2	1	0.27
1	13	S	F	1	1	0.04
1	13	S	M	1	1	0.03
1	13	S	F	2	1	0.04
1	13	S	M	2	1	0.14
1	14	FB	F	1	1	0.00
1	14	FB	M	1	1	0.25
1	14	FB	F	2	1	0.10
1	14	FB	M	2	1	0.32
1	15	FA	F	1	1	0.00
1	15	FA	M	1	1	0.00
1	15	FA	F	2	1	0.00
1	15	FA	M	2	1	0.25
1	16	FC	F	1	1	0.00

1	16	FC	M	1	1	0.00
1	16	FC	F	2	1	0.32
1	16	FC	M	2	1	0.58
2	1	FA	F	1	1	0.04
2	1	FA	M	1	1	0.11
2	1	FA	F	2	1	0.35
2	1	FA	M	2	1	0.46
2	2	FB	F	1	1	0.00
2	2	FB	M	1	1	0.00
2	2	FB	F	2	1	0.08
2	2	FB	M	2	1	0.19
2	3	S	F	1	1	0.00
2	3	S	M	1	1	0.00
2	3	S	F	2	1	0.00
2	3	S	M	2	1	0.00
2	4	FC	F	1	1	0.00
2	4	FC	M	1	1	0.00
2	4	FC	F	2	1	0.04
2	4	FC	M	2	1	0.23
2	5	FA	F	1	1	0.15
2	5	FA	M	1	1	0.00
2	5	FA	F	2	1	0.04
2	5	FA	M	2	1	0.13
2	6	FB	F	1	1	0.05
2	6	FB	M	1	1	0.00
2	6	FB	F	2	1	0.05
2	6	FB	M	2	1	0.00
2	7	S	F	1	1	0.00
2	7	S	M	1	1	0.00
2	7	S	F	2	1	0.00
2	7	S	M	2	1	0.00
2	8	FC	F	1	1	0.00
2	8	FC	M	1	1	0.00
2	8	FC	F	2	1	0.00
2	8	FC	M	2	1	0.04
2	9	FB	F	1	1	0.08
2	9	FB	M	1	1	0.04
2	9	FB	F	2	1	0.00
2	9	FB	M	2	1	0.25
2	10	S	F	1	1	0.00
2	10	S	M	1	1	0.00
2	10	S	F	2	1	0.00
2	10	S	M	2	1	0.06
2	11	FC	F	1	1	0.08
2	11	FC	M	1	1	0.00
2	11	FC	F	2	1	0.18
2	11	FC	M	2	1	0.14
2	12	FA	F	1	1	0.00
2	12	FA	M	1	1	0.09
2	12	FA	F	2	1	0.08
2	12	FA	M	2	1	0.08
2	13	FB	F	1	1	0.04
2	13	FB	M	1	1	0.00

2	13	FB	F	2	1	0.18
2	13	FB	M	2	1	0.32
2	14	S	F	1	1	0.00
2	14	S	M	1	1	0.00
2	14	S	F	2	1	0.00
2	14	S	M	2	1	0.00
2	15	FC	F	1	1	0.13
2	15	FC	M	1	1	0.04
2	15	FC	F	2	1	0.11
2	15	FC	M	2	1	0.19
2	16	FA	F	1	1	0.00
2	16	FA	M	1	1	0.00
2	16	FA	F	2	1	0.08
2	16	FA	M	2	1	0.17
1	1	FB	F	1	2	0.00
1	1	FB	M	1	2	0.00
1	1	FB	F	2	2	0.00
1	1	FB	M	2	2	0.00
1	2	FA	F	1	2	0.00
1	2	FA	M	1	2	0.00
1	2	FA	F	2	2	0.00
1	2	FA	M	2	2	0.00
1	3	FC	F	1	2	0.00
1	3	FC	M	1	2	0.00
1	3	FC	F	2	2	0.00
1	3	FC	M	2	2	0.00
1	4	S	F	1	2	0.00
1	4	S	M	1	2	0.00
1	4	S	F	2	2	0.00
1	4	S	M	2	2	0.00
1	5	FB	F	1	2	0.00
1	5	FB	M	1	2	0.00
1	5	FB	F	2	2	0.00
1	5	FB	M	2	2	0.00
1	6	FA	F	1	2	0.00
1	6	FA	M	1	2	0.00
1	6	FA	F	2	2	0.00
1	6	FA	M	2	2	0.00
1	7	FC	F	1	2	0.00
1	7	FC	M	1	2	0.00
1	7	FC	F	2	2	0.00
1	7	FC	M	2	2	0.05
1	8	S	F	1	2	0.00
1	8	S	M	1	2	0.00
1	8	S	F	2	2	0.00
1	8	S	M	2	2	0.00
1	9	S	F	1	2	0.00
1	9	S	M	1	2	0.00
1	9	S	F	2	2	0.00
1	9	S	M	2	2	0.00
1	10	FB	F	1	2	0.00
1	10	FB	M	1	2	0.00
1	10	FB	F	2	2	0.00

1	10	FB	M	2	2	0.00
1	11	FA	F	1	2	0.00
1	11	FA	M	1	2	0.00
1	11	FA	F	2	2	0.00
1	11	FA	M	2	2	0.00
1	12	FC	F	1	2	0.00
1	12	FC	M	1	2	0.00
1	12	FC	F	2	2	0.00
1	12	FC	M	2	2	0.00
1	13	S	F	1	2	0.00
1	13	S	M	1	2	0.00
1	13	S	F	2	2	0.00
1	13	S	M	2	2	0.00
1	14	FB	F	1	2	0.00
1	14	FB	M	1	2	0.00
1	14	FB	F	2	2	0.00
1	14	FB	M	2	2	0.06
1	15	FA	F	1	2	0.00
1	15	FA	M	1	2	0.00
1	15	FA	F	2	2	0.00
1	15	FA	M	2	2	0.00
1	16	FC	F	1	2	0.00
1	16	FC	M	1	2	0.00
1	16	FC	F	2	2	0.00
1	16	FC	M	2	2	0.00
2	1	FA	F	1	2	0.00
2	1	FA	M	1	2	0.00
2	1	FA	F	2	2	0.00
2	1	FA	M	2	2	0.00
2	2	FB	F	1	2	0.00
2	2	FB	M	1	2	0.00
2	2	FB	F	2	2	0.00
2	2	FB	M	2	2	0.00
2	3	S	F	1	2	0.00
2	3	S	M	1	2	0.00
2	3	S	F	2	2	0.00
2	3	S	M	2	2	0.00
2	4	FC	F	1	2	0.00
2	4	FC	M	1	2	0.00
2	4	FC	F	2	2	0.00
2	4	FC	M	2	2	0.00
2	5	FA	F	1	2	0.00
2	5	FA	M	1	2	0.00
2	5	FA	F	2	2	0.00
2	5	FA	M	2	2	0.00
2	6	FB	F	1	2	0.00
2	6	FB	M	1	2	0.00
2	6	FB	F	2	2	0.00
2	6	FB	M	2	2	0.00
2	7	S	F	1	2	0.00
2	7	S	M	1	2	0.00
2	7	S	F	2	2	0.00
2	7	S	M	2	2	0.00

2	8	FC	F	1	2	0.00
2	8	FC	M	1	2	0.00
2	8	FC	F	2	2	0.00
2	8	FC	M	2	2	0.00
2	9	FB	F	1	2	0.00
2	9	FB	M	1	2	0.00
2	9	FB	F	2	2	0.00
2	9	FB	M	2	2	0.00
2	10	S	F	1	2	0.00
2	10	S	M	1	2	0.00
2	10	S	F	2	2	0.00
2	10	S	M	2	2	0.00
2	11	FC	F	1	2	0.00
2	11	FC	M	1	2	0.00
2	11	FC	F	2	2	0.00
2	11	FC	M	2	2	0.00
2	12	FA	F	1	2	0.00
2	12	FA	M	1	2	0.00
2	12	FA	F	2	2	0.00
2	12	FA	M	2	2	0.00
2	13	FB	F	1	2	0.00
2	13	FB	M	1	2	0.00
2	13	FB	F	2	2	0.00
2	13	FB	M	2	2	0.00
2	14	S	F	1	2	0.00
2	14	S	M	1	2	0.00
2	14	S	F	2	2	0.00
2	14	S	M	2	2	0.00
2	15	FC	F	1	2	0.00
2	15	FC	M	1	2	0.00
2	15	FC	F	2	2	0.00
2	15	FC	M	2	2	0.00
2	16	FA	F	1	2	0.00
2	16	FA	M	1	2	0.00
2	16	FA	F	2	2	0.00
2	16	FA	M	2	2	0.00
