# Supplementary material for: Slow and steady wins the race: The behaviour and welfare of commercial faster growing broiler breeds compared to a commercial slower growing breed
Source: PLoS One. 2020 Apr 6;15(4):e0231006. doi: 10.1371/journal.pone.0231006 (PMC7135253; doi:10.1371/journal.pone.0231006)
Supplement: S10 Data — (PDF) [file pone.0231006.s010.pdf]

Replicate	Pen	Breed	age (d)	Lights	PropFeed	PropDrink	PropSit	PropStand	PropForag	PropLocon	PropPerch	PropPreen	PropOther
1	1	FB	2	OFF	0.00	0.00	0.96	0.00	0.01	0.03	0.00	0.00	0.00
1	1	FB	2	ON	0.11	0.07	0.43	0.14	0.08	0.14	0.00	0.02	0.00
1	10	FB	2	OFF	0.00	0.00	0.81	0.04	0.11	0.01	0.00	0.02	0.00
1	10	FB	2	ON	0.03	0.03	0.63	0.14	0.12	0.05	0.00	0.01	0.00
1	11	FA	2	OFF	0.04	0.00	0.74	0.12	0.04	0.05	0.00	0.00	0.00
1	11	FA	2	ON	0.05	0.04	0.54	0.18	0.12	0.06	0.00	0.01	0.00
1	12	FC	2	OFF	0.02	0.02	0.75	0.14	0.03	0.03	0.00	0.00	0.00
1	12	FC	2	ON	0.03	0.03	0.53	0.17	0.14	0.09	0.00	0.01	0.00
1	13	S	2	OFF	0.00	0.00	1.00	0.00	0.00	0.00	0.00	0.00	0.00
1	13	S	2	ON	0.02	0.03	0.56	0.14	0.15	0.08	0.00	0.03	0.00
1	14	FB	2	OFF	0.02	0.01	0.86	0.04	0.02	0.04	0.00	0.00	0.00
1	14	FB	2	ON	0.03	0.04	0.52	0.19	0.15	0.07	0.00	0.01	0.00
1	15	FA	2	OFF	0.00	0.01	0.95	0.04	0.00	0.00	0.00	0.00	0.00
1	15	FA	2	ON	0.04	0.03	0.55	0.19	0.13	0.05	0.00	0.01	0.00
1	16	FC	2	OFF	0.00	0.00	0.92	0.07	0.00	0.01	0.00	0.00	0.00
1	16	FC	2	ON	0.03	0.03	0.57	0.17	0.14	0.06	0.00	0.01	0.00
1	2	FA	2	OFF	0.00	0.00	0.96	0.01	0.00	0.03	0.00	0.00	0.00
1	2	FA	2	ON	0.08	0.05	0.50	0.13	0.15	0.08	0.00	0.01	0.00
1	3	FC	2	OFF	0.02	0.00	0.89	0.06	0.00	0.02	0.00	0.00	0.00
1	3	FC	2	ON	0.06	0.04	0.47	0.15	0.17	0.10	0.00	0.01	0.00
1	4	S	2	OFF	0.00	0.02	0.91	0.00	0.06	0.00	0.00	0.00	0.00
1	4	S	2	ON	0.03	0.07	0.45	0.15	0.15	0.12	0.00	0.01	0.00
1	5	FB	2	OFF	0.07	0.02	0.79	0.04	0.07	0.01	0.00	0.00	0.00
1	5	FB	2	ON	0.04	0.04	0.60	0.15	0.12	0.04	0.00	0.02	0.00
1	6	FA	2	OFF	0.03	0.02	0.77	0.10	0.02	0.05	0.00	0.00	0.00
1	6	FA	2	ON	0.06	0.04	0.51	0.15	0.14	0.08	0.00	0.02	0.00
1	7	FC	2	OFF	0.00	0.04	0.88	0.07	0.00	0.01	0.00	0.00	0.00
1	7	FC	2	ON	0.03	0.04	0.61	0.10	0.12	0.09	0.00	0.01	0.00
1	8	S	2	OFF	0.01	0.03	0.91	0.00	0.00	0.05	0.00	0.00	0.00
1	8	S	2	ON	0.03	0.02	0.63	0.10	0.15	0.07	0.00	0.01	0.00
1	9	S	2	OFF	0.00	0.00	0.91	0.06	0.00	0.00	0.00	0.03	0.00
1	9	S	2	ON	0.03	0.03	0.53	0.16	0.16	0.07	0.00	0.02	0.00
1	1	FB	9	OFF	0.00	0.00	0.99	0.01	0.00	0.00	0.00	0.00	0.00
1	1	FB	9	ON	0.13	0.07	0.42	0.13	0.15	0.06	0.01	0.02	0.00
1	10	FB	9	OFF	0.01	0.00	0.96	0.01	0.01	0.00	0.00	0.01	0.00
1	10	FB	9	ON	0.10	0.02	0.55	0.06	0.18	0.05	0.02	0.01	0.00
1	11	FA	9	OFF	0.00	0.00	0.98	0.02	0.01	0.00	0.00	0.00	0.00
1	11	FA	9	ON	0.14	0.04	0.40	0.08	0.23	0.10	0.00	0.01	0.00
1	12	FC	9	OFF	0.00	0.00	0.98	0.01	0.00	0.00	0.00	0.00	0.00
1	12	FC	9	ON	0.16	0.05	0.37	0.07	0.27	0.07	0.01	0.01	0.00
1	13	S	9	OFF	0.00	0.00	1.00	0.00	0.00	0.00	0.00	0.00	0.00
1	13	S	9	ON	0.12	0.03	0.44	0.08	0.18	0.09	0.03	0.03	0.00
1	14	FB	9	OFF	0.00	0.00	1.00	0.00	0.00	0.00	0.00	0.00	0.00
1	14	FB	9	ON	0.12	0.03	0.43	0.11	0.16	0.10	0.03	0.02	0.00
1	15	FA	9	OFF	0.00	0.00	1.00	0.00	0.00	0.00	0.00	0.00	0.00
1	15	FA	9	ON	0.18	0.03	0.47	0.06	0.13	0.09	0.01	0.02	0.00
1	16	FC	9	OFF	0.00	0.00	0.99	0.01	0.00	0.00	0.00	0.00	0.00
1	16	FC	9	ON	0.12	0.04	0.49	0.10	0.12	0.10	0.00	0.02	0.00
1	2	FA	9	OFF	0.00	0.00	0.99	0.01	0.00	0.00	0.00	0.00	0.00
1	2	FA	9	ON	0.13	0.04	0.50	0.11	0.09	0.11	0.01	0.02	0.00
1	3	FC	9	OFF	0.00	0.00	0.98	0.02	0.00	0.00	0.00	0.00	0.00
1	3	FC	9	ON	0.10	0.04	0.47	0.15	0.13	0.09	0.01	0.00	0.01
1	4	S	9	OFF	0.00	0.00	1.00	0.00	0.00	0.00	0.00	0.00	0.00
1	4	S	9	ON	0.10	0.04	0.39	0.11	0.21	0.10	0.02	0.01	0.01
1	5	FB	9	OFF	0.00	0.00	0.99	0.01	0.00	0.00	0.00	0.00	0.00
1	5	FB	9	ON	0.16	0.07	0.44	0.11	0.09	0.09	0.01	0.01	0.00
1	6	FA	9	OFF	0.00	0.00	0.97	0.01	0.00	0.01	0.00	0.00	0.00
1	6	FA	9	ON	0.12	0.05	0.56	0.09	0.09	0.06	0.01	0.01	0.00
1	7	FC	9	OFF	0.00	0.00	1.00	0.00	0.00	0.00	0.00	0.00	0.00
1	7	FC	9	ON	0.14	0.05	0.44	0.09	0.15	0.10	0.00	0.02	0.00
1	8	S	9	OFF	0.00	0.00	0.98	0.02	0.00	0.00	0.00	0.00	0.00
1	8	S	9	ON	0.10	0.02	0.47	0.07	0.15	0.11	0.03	0.04	0.00
1	9	S	9	OFF	0.00	0.01	0.97	0.02	0.00	0.00	0.00	0.00	0.00
1	9	S	9	ON	0.14	0.03	0.39	0.15	0.16	0.09	0.01	0.01	0.00
1	1	FB	16	OFF	0.00	0.00	0.99	0.01	0.00	0.00	0.00	0.00	0.00
1	1	FB	16	ON	0.08	0.03	0.62	0.07	0.08	0.07	0.02	0.03	0.01
1	10	FB	16	OFF	0.00	0.00	0.96	0.02	0.00	0.00	0.02	0.00	0.00
1	10	FB	16	ON	0.07	0.03	0.60	0.09	0.10	0.04	0.04	0.02	0.01
1	11	FA	16	OFF	0.00	0.00	0.99	0.01	0.00	0.00	0.00	0.00	0.00
1	11	FA	16	ON	0.08	0.05	0.68	0.05	0.06	0.04	0.01	0.03	0.01
1	12	FC	16	OFF	0.00	0.00	0.98	0.02	0.00	0.00	0.00	0.00	0.00
1	12	FC	16	ON	0.08	0.05	0.51	0.08	0.15	0.06	0.03	0.03	0.01
1	13	S	16	OFF	0.00	0.00	0.96	0.02	0.00	0.00	0.02	0.00	0.00
1	13	S	16	ON	0.04	0.01	0.44	0.12	0.16	0.04	0.13	0.05	0.01
1	14	FB	16	OFF	0.00	0.00	0.99	0.01	0.00	0.00	0.00	0.00	0.00

1	14	FB	16	ON	0.07	0.03	0.57	0.08	0.09	0.04	0.05	0.05	0.01
1	15	FA	16	OFF	0.00	0.00	0.98	0.02	0.00	0.00	0.00	0.00	0.00
1	15	FA	16	ON	0.08	0.04	0.66	0.07	0.06	0.04	0.01	0.04	0.01
1	16	FC	16	OFF	0.00	0.00	0.98	0.02	0.00	0.00	0.00	0.00	0.00
1	16	FC	16	ON	0.08	0.03	0.58	0.09	0.15	0.04	0.01	0.03	0.00
1	2	FA	16	OFF	0.00	0.00	0.96	0.03	0.00	0.01	0.00	0.00	0.00
1	2	FA	16	ON	0.09	0.05	0.62	0.08	0.06	0.05	0.01	0.04	0.00
1	3	FC	16	OFF	0.00	0.00	0.98	0.02	0.00	0.00	0.00	0.00	0.00
1	3	FC	16	ON	0.11	0.05	0.57	0.08	0.09	0.06	0.02	0.03	0.01
1	4	S	16	OFF	0.00	0.00	0.97	0.01	0.00	0.01	0.01	0.01	0.00
1	4	S	16	ON	0.05	0.02	0.39	0.18	0.12	0.12	0.08	0.03	0.01
1	5	FB	16	OFF	0.00	0.00	0.98	0.02	0.00	0.00	0.00	0.00	0.00
1	5	FB	16	ON	0.08	0.02	0.58	0.12	0.08	0.07	0.02	0.03	0.00
1	6	FA	16	OFF	0.00	0.00	0.65	0.35	0.00	0.00	0.00	0.00	0.00
1	6	FA	16	ON	0.09	0.03	0.63	0.10	0.06	0.05	0.01	0.04	0.01
1	7	FC	16	OFF	0.00	0.00	0.98	0.01	0.00	0.00	0.00	0.00	0.00
1	7	FC	16	ON	0.09	0.03	0.62	0.10	0.06	0.05	0.01	0.04	0.01
1	8	S	16	OFF	0.00	0.00	0.97	0.02	0.00	0.01	0.00	0.00	0.00
1	8	S	16	ON	0.05	0.02	0.40	0.10	0.18	0.08	0.10	0.05	0.01
1	9	S	16	OFF	0.00	0.00	0.98	0.02	0.00	0.00	0.00	0.00	0.00
1	9	S	16	ON	0.04	0.03	0.46	0.09	0.16	0.07	0.11	0.03	0.01
1	1	FB	23	OFF	0.00	0.00	0.94	0.03	0.00	0.00	0.02	0.00	0.00
1	1	FB	23	ON	0.06	0.03	0.67	0.09	0.06	0.04	0.01	0.03	0.00
1	10	FB	23	OFF	0.00	0.00	0.94	0.04	0.00	0.00	0.02	0.00	0.00
1	10	FB	23	ON	0.07	0.02	0.66	0.10	0.06	0.03	0.02	0.04	0.00
1	11	FA	23	OFF	0.00	0.00	0.96	0.04	0.00	0.00	0.00	0.00	0.00
1	11	FA	23	ON	0.08	0.04	0.69	0.09	0.02	0.03	0.01	0.04	0.01
1	12	FC	23	OFF	0.00	0.00	0.93	0.05	0.00	0.00	0.02	0.00	0.00
1	12	FC	23	ON	0.10	0.03	0.61	0.11	0.05	0.04	0.02	0.03	0.01
1	13	S	23	OFF	0.00	0.00	0.91	0.07	0.00	0.00	0.01	0.00	0.00
1	13	S	23	ON	0.05	0.01	0.41	0.13	0.13	0.06	0.15	0.05	0.01
1	14	FB	23	OFF	0.00	0.00	0.96	0.04	0.00	0.00	0.00	0.00	0.00
1	14	FB	23	ON	0.08	0.03	0.61	0.08	0.10	0.04	0.02	0.05	0.00
1	15	FA	23	OFF	0.00	0.00	0.97	0.03	0.00	0.00	0.00	0.00	0.00
1	15	FA	23	ON	0.08	0.02	0.64	0.08	0.08	0.04	0.02	0.04	0.01
1	16	FC	23	OFF	0.00	0.00	0.99	0.01	0.00	0.00	0.00	0.00	0.00
1	16	FC	23	ON	0.07	0.03	0.56	0.15	0.07	0.05	0.01	0.05	0.01
1	2	FA	23	OFF	0.00	0.00	0.93	0.05	0.00	0.00	0.02	0.00	0.00
1	2	FA	23	ON	0.06	0.05	0.70	0.07	0.04	0.03	0.00	0.04	0.00
1	3	FC	23	OFF	0.00	0.00	0.95	0.04	0.00	0.01	0.00	0.00	0.00
1	3	FC	23	ON	0.08	0.02	0.65	0.07	0.08	0.04	0.01	0.05	0.00
1	4	S	23	OFF	0.00	0.00	0.88	0.04	0.00	0.00	0.07	0.00	0.00
1	4	S	23	ON	0.03	0.02	0.40	0.18	0.11	0.09	0.12	0.06	0.00
1	5	FB	23	OFF	0.00	0.00	0.96	0.03	0.00	0.00	0.00	0.00	0.00
1	5	FB	23	ON	0.07	0.04	0.58	0.11	0.08	0.06	0.01	0.03	0.00
1	6	FA	23	OFF	0.00	0.00	0.97	0.03	0.00	0.00	0.00	0.00	0.00
1	6	FA	23	ON	0.07	0.03	0.67	0.07	0.05	0.04	0.00	0.06	0.00
1	7	FC	23	OFF	0.00	0.00	0.97	0.02	0.00	0.00	0.00	0.00	0.00
1	7	FC	23	ON	0.07	0.05	0.60	0.11	0.07	0.03	0.02	0.05	0.01
1	8	S	23	OFF	0.00	0.00	0.76	0.21	0.00	0.01	0.03	0.00	0.00
1	8	S	23	ON	0.05	0.02	0.38	0.11	0.14	0.07	0.14	0.06	0.01
1	9	S	23	OFF	0.00	0.00	0.90	0.02	0.00	0.00	0.08	0.00	0.00
1	9	S	23	ON	0.02	0.01	0.53	0.17	0.11	0.03	0.09	0.04	0.01
1	1	FB	30	OFF	0.00	0.00	0.94	0.05	0.00	0.00	0.00	0.00	0.00
1	1	FB	30	ON	0.06	0.04	0.65	0.10	0.07	0.02	0.00	0.05	0.00
1	10	FB	30	OFF	0.00	0.00	0.94	0.05	0.00	0.00	0.01	0.00	0.00
1	10	FB	30	ON	0.06	0.03	0.70	0.06	0.07	0.02	0.01	0.04	0.00
1	11	FA	30	OFF	0.00	0.00	0.96	0.04	0.00	0.00	0.00	0.00	0.00
1	11	FA	30	ON	0.08	0.05	0.68	0.05	0.05	0.03	0.00	0.05	0.00
1	12	FC	30	OFF	0.00	0.00	0.47	0.48	0.01	0.00	0.04	0.00	0.00
1	12	FC	30	ON	0.07	0.04	0.70	0.04	0.06	0.02	0.01	0.05	0.01
1	13	S	30	OFF	0.00	0.00	0.89	0.04	0.00	0.00	0.07	0.00	0.00
1	13	S	30	ON	0.04	0.02	0.46	0.13	0.09	0.07	0.13	0.04	0.01
1	14	FB	30	OFF	0.00	0.00	0.92	0.04	0.00	0.00	0.04	0.00	0.00
1	14	FB	30	ON	0.07	0.02	0.66	0.05	0.09	0.03	0.01	0.07	0.01
1	15	FA	30	OFF	0.00	0.00	0.96	0.03	0.00	0.00	0.00	0.01	0.00
1	15	FA	30	ON	0.06	0.04	0.73	0.05	0.03	0.02	0.00	0.05	0.01
1	16	FC	30	OFF	0.00	0.00	0.97	0.03	0.00	0.00	0.00	0.00	0.00
1	16	FC	30	ON	0.06	0.03	0.68	0.07	0.07	0.02	0.01	0.06	0.01
1	2	FA	30	OFF	0.00	0.00	0.97	0.03	0.00	0.00	0.00	0.00	0.00
1	2	FA	30	ON	0.06	0.04	0.72	0.07	0.04	0.02	0.00	0.04	0.01
1	3	FC	30	OFF	0.00	0.00	0.77	0.21	0.02	0.00	0.00	0.00	0.00
1	3	FC	30	ON	0.08	0.03	0.65	0.07	0.05	0.04	0.01	0.06	0.00
1	4	S	30	OFF	0.00	0.00	0.92	0.03	0.00	0.01	0.04	0.00	0.00
1	4	S	30	ON	0.05	0.03	0.50	0.10	0.11	0.05	0.10	0.04	0.01
1	5	FB	30	OFF	0.00	0.00	0.98	0.02	0.00	0.00	0.00	0.00	0.00

1	5	FB	30	ON	0.07	0.04	0.64	0.09	0.06	0.04	0.01	0.05	0.01
1	6	FA	30	OFF	0.00	0.00	0.96	0.03	0.00	0.00	0.00	0.00	0.00
1	6	FA	30	ON	0.07	0.04	0.68	0.06	0.06	0.03	0.00	0.05	0.00
1	7	FC	30	OFF	0.00	0.00	0.95	0.04	0.00	0.00	0.00	0.00	0.00
1	7	FC	30	ON	0.06	0.03	0.65	0.04	0.10	0.03	0.02	0.06	0.01
1	8	S	30	OFF	0.00	0.00	0.94	0.04	0.00	0.00	0.02	0.00	0.00
1	8	S	30	ON	0.02	0.01	0.48	0.09	0.11	0.05	0.16	0.06	0.01
1	9	S	30	OFF	0.00	0.00	0.89	0.01	0.00	0.00	0.10	0.00	0.00
1	9	S	30	ON	0.03	0.00	0.57	0.09	0.10	0.02	0.09	0.08	0.01
1	1	FB	37	OFF	0.00	0.00	0.97	0.03	0.00	0.00	0.00	0.00	0.00
1	1	FB	37	ON	0.06	0.03	0.71	0.06	0.05	0.03	0.00	0.06	0.00
1	10	FB	37	OFF	0.00	0.00	0.96	0.03	0.00	0.00	0.00	0.00	0.00
1	10	FB	37	ON	0.04	0.03	0.78	0.04	0.03	0.02	0.00	0.06	0.00
1	11	FA	37	OFF	0.00	0.00	0.96	0.04	0.00	0.00	0.00	0.00	0.00
1	11	FA	37	ON	0.04	0.03	0.78	0.04	0.03	0.02	0.00	0.06	0.00
1	12	FC	37	OFF	0.00	0.00	0.99	0.01	0.00	0.00	0.00	0.00	0.00
1	12	FC	37	ON	0.05	0.03	0.77	0.05	0.04	0.02	0.00	0.05	0.00
1	13	S	37	OFF	0.00	0.00	0.87	0.04	0.00	0.00	0.09	0.00	0.00
1	13	S	37	ON	0.03	0.01	0.44	0.13	0.10	0.05	0.12	0.10	0.02
1	14	FB	37	OFF	0.00	0.00	0.96	0.03	0.00	0.00	0.00	0.00	0.00
1	14	FB	37	ON	0.05	0.03	0.72	0.04	0.05	0.03	0.00	0.08	0.00
1	15	FA	37	OFF	0.00	0.00	0.98	0.02	0.00	0.00	0.00	0.00	0.00
1	15	FA	37	ON	0.06	0.04	0.74	0.02	0.05	0.02	0.00	0.07	0.01
1	16	FC	37	OFF	0.00	0.00	0.96	0.04	0.00	0.00	0.00	0.00	0.00
1	16	FC	37	ON	0.06	0.03	0.72	0.06	0.03	0.02	0.00	0.06	0.01
1	2	FA	37	OFF	0.00	0.00	0.98	0.02	0.00	0.00	0.00	0.00	0.00
1	2	FA	37	ON	0.06	0.05	0.74	0.04	0.03	0.02	0.00	0.06	0.00
1	3	FC	37	OFF	0.00	0.00	0.97	0.03	0.00	0.00	0.00	0.00	0.00
1	3	FC	37	ON	0.06	0.04	0.69	0.06	0.06	0.04	0.00	0.06	0.00
1	4	S	37	OFF	0.00	0.00	0.88	0.04	0.00	0.00	0.07	0.01	0.00
1	4	S	37	ON	0.04	0.02	0.53	0.12	0.07	0.05	0.09	0.07	0.02
1	5	FB	37	OFF	0.00	0.00	0.92	0.08	0.00	0.00	0.00	0.00	0.00
1	5	FB	37	ON	0.04	0.05	0.69	0.06	0.06	0.04	0.00	0.06	0.00
1	6	FA	37	OFF	0.00	0.00	0.97	0.03	0.00	0.00	0.00	0.00	0.00
1	6	FA	37	ON	0.08	0.04	0.74	0.03	0.03	0.03	0.00	0.06	0.00
1	7	FC	37	OFF	0.00	0.00	0.94	0.06	0.00	0.00	0.00	0.00	0.00
1	7	FC	37	ON	0.07	0.03	0.69	0.04	0.06	0.03	0.01	0.07	0.00
1	8	S	37	OFF	0.00	0.00	0.92	0.05	0.00	0.00	0.03	0.00	0.00
1	8	S	37	ON	0.03	0.02	0.51	0.12	0.06	0.06	0.12	0.08	0.01
1	9	S	37	OFF	0.00	0.00	0.87	0.00	0.00	0.00	0.13	0.00	0.00
1	9	S	37	ON	0.04	0.01	0.56	0.11	0.07	0.05	0.06	0.08	0.01
2	1	FA	2	OFF	0.00	0.00	0.98	0.00	0.00	0.02	0.00	0.00	0.00
2	1	FA	2	ON	0.06	0.06	0.57	0.13	0.11	0.07	0.00	0.01	0.00
2	10	S	2	OFF	0.00	0.00	0.93	0.05	0.00	0.00	0.00	0.02	0.00
2	10	S	2	ON	0.03	0.02	0.63	0.14	0.11	0.06	0.00	0.01	0.00
2	11	FC	2	OFF	0.00	0.00	0.98	0.00	0.01	0.01	0.00	0.00	0.00
2	11	FC	2	ON	0.06	0.05	0.48	0.15	0.19	0.06	0.00	0.01	0.00
2	12	FA	2	OFF	0.00	0.00	0.94	0.00	0.04	0.02	0.00	0.00	0.00
2	12	FA	2	ON	0.04	0.02	0.60	0.16	0.11	0.07	0.00	0.00	0.00
2	13	FB	2	OFF	0.00	0.00	0.93	0.05	0.00	0.00	0.00	0.02	0.00
2	13	FB	2	ON	0.04	0.02	0.55	0.19	0.14	0.06	0.00	0.00	0.00
2	14	S	2	OFF	0.00	0.00	0.93	0.07	0.00	0.00	0.00	0.00	0.00
2	14	S	2	ON	0.02	0.02	0.54	0.17	0.20	0.06	0.00	0.01	0.00
2	15	FC	2	OFF	0.01	0.01	0.94	0.00	0.04	0.00	0.00	0.00	0.00
2	15	FC	2	ON	0.01	0.03	0.61	0.17	0.09	0.08	0.00	0.00	0.00
2	16	FA	2	OFF	0.00	0.00	0.85	0.09	0.06	0.00	0.00	0.00	0.00
2	16	FA	2	ON	0.04	0.02	0.57	0.17	0.10	0.10	0.00	0.01	0.00
2	2	FB	2	OFF	0.02	0.00	0.87	0.07	0.03	0.01	0.00	0.00	0.00
2	2	FB	2	ON	0.03	0.04	0.54	0.21	0.10	0.08	0.00	0.01	0.00
2	3	S	2	OFF	0.00	0.00	0.95	0.03	0.00	0.02	0.00	0.00	0.00
2	3	S	2	ON	0.02	0.03	0.55	0.18	0.14	0.07	0.00	0.01	0.00
2	4	FC	2	OFF	0.00	0.00	0.96	0.04	0.00	0.00	0.00	0.00	0.00
2	4	FC	2	ON	0.04	0.05	0.63	0.13	0.10	0.05	0.00	0.00	0.00
2	5	FA	2	OFF	0.00	0.00	0.95	0.05	0.00	0.00	0.00	0.00	0.00
2	5	FA	2	ON	0.03	0.04	0.52	0.23	0.10	0.08	0.00	0.01	0.00
2	6	FB	2	OFF	0.02	0.00	0.97	0.01	0.00	0.00	0.00	0.00	0.00
2	6	FB	2	ON	0.03	0.04	0.66	0.12	0.08	0.06	0.00	0.00	0.00
2	7	S	2	OFF	0.00	0.00	0.94	0.05	0.00	0.01	0.00	0.00	0.00
2	7	S	2	ON	0.03	0.03	0.65	0.10	0.14	0.05	0.00	0.01	0.00
2	8	FC	2	OFF	0.00	0.00	0.98	0.02	0.00	0.00	0.00	0.00	0.00
2	8	FC	2	ON	0.02	0.05	0.65	0.10	0.13	0.05	0.00	0.00	0.00
2	9	FB	2	OFF	0.00	0.01	0.95	0.03	0.00	0.01	0.00	0.00	0.00
2	9	FB	2	ON	0.04	0.04	0.61	0.11	0.13	0.06	0.00	0.00	0.00
2	1	FA	9	OFF	0.00	0.00	0.99	0.01	0.00	0.00	0.00	0.00	0.00
2	1	FA	9	ON	0.17	0.04	0.42	0.07	0.16	0.09	0.03	0.02	0.00
2	10	S	9	OFF	0.00	0.00	0.98	0.02	0.00	0.00	0.00	0.00	0.00

2	10	S	9	ON	0.13	0.03	0.52	0.05	0.13	0.09	0.02	0.02	0.00
2	11	FC	9	OFF	0.00	0.00	1.00	0.00	0.00	0.00	0.00	0.00	0.00
2	11	FC	9	ON	0.14	0.04	0.41	0.07	0.22	0.09	0.00	0.02	0.00
2	12	FA	9	OFF	0.00	0.00	0.99	0.01	0.00	0.00	0.00	0.00	0.00
2	12	FA	9	ON	0.13	0.04	0.37	0.08	0.24	0.11	0.00	0.02	0.00
2	13	FB	9	OFF	0.00	0.00	0.99	0.01	0.00	0.00	0.00	0.00	0.00
2	13	FB	9	ON	0.17	0.04	0.35	0.12	0.17	0.09	0.04	0.01	0.00
2	14	S	9	OFF	0.00	0.00	0.32	0.67	0.01	0.00	0.00	0.00	0.00
2	14	S	9	ON	0.10	0.02	0.39	0.11	0.22	0.08	0.04	0.03	0.01
2	15	FC	9	OFF	0.00	0.00	0.99	0.01	0.00	0.00	0.00	0.00	0.00
2	15	FC	9	ON	0.14	0.03	0.42	0.12	0.16	0.10	0.01	0.01	0.00
2	16	FA	9	OFF	0.00	0.00	0.97	0.01	0.01	0.01	0.00	0.00	0.00
2	16	FA	9	ON	0.13	0.05	0.43	0.11	0.17	0.07	0.02	0.02	0.00
2	2	FB	9	OFF	0.01	0.00	0.96	0.01	0.01	0.00	0.01	0.00	0.00
2	2	FB	9	ON	0.15	0.04	0.38	0.07	0.21	0.10	0.03	0.01	0.00
2	3	S	9	OFF	0.00	0.00	0.97	0.02	0.01	0.00	0.00	0.00	0.00
2	3	S	9	ON	0.13	0.04	0.43	0.06	0.14	0.09	0.08	0.03	0.00
2	4	FC	9	OFF	0.01	0.00	0.97	0.01	0.01	0.00	0.00	0.00	0.00
2	4	FC	9	ON	0.16	0.05	0.46	0.10	0.15	0.07	0.00	0.01	0.00
2	5	FA	9	OFF	0.00	0.00	0.98	0.02	0.00	0.00	0.00	0.00	0.00
2	5	FA	9	ON	0.18	0.04	0.49	0.05	0.12	0.11	0.00	0.01	0.00
2	6	FB	9	OFF	0.00	0.00	0.96	0.02	0.01	0.01	0.00	0.00	0.00
2	6	FB	9	ON	0.12	0.05	0.51	0.07	0.14	0.09	0.01	0.02	0.00
2	7	S	9	OFF	0.00	0.00	0.99	0.01	0.00	0.00	0.00	0.00	0.00
2	7	S	9	ON	0.11	0.02	0.41	0.05	0.28	0.08	0.03	0.02	0.00
2	8	FC	9	OFF	0.00	0.00	0.98	0.02	0.00	0.00	0.00	0.00	0.00
2	8	FC	9	ON	0.16	0.06	0.46	0.06	0.13	0.07	0.04	0.02	0.00
2	9	FB	9	OFF	0.00	0.00	0.97	0.02	0.00	0.01	0.00	0.00	0.00
2	9	FB	9	ON	0.13	0.04	0.38	0.09	0.20	0.12	0.01	0.02	0.00
2	1	FA	16	OFF	0.00	0.00	0.98	0.02	0.00	0.00	0.00	0.00	0.00
2	1	FA	16	ON	0.09	0.03	0.58	0.09	0.09	0.06	0.02	0.03	0.01
2	10	S	16	OFF	0.00	0.00	0.92	0.04	0.00	0.00	0.03	0.00	0.00
2	10	S	16	ON	0.05	0.03	0.48	0.14	0.11	0.05	0.09	0.04	0.01
2	11	FC	16	OFF	0.00	0.00	0.93	0.02	0.00	0.01	0.02	0.01	0.00
2	11	FC	16	ON	0.10	0.04	0.46	0.09	0.15	0.10	0.03	0.02	0.01
2	12	FA	16	OFF	0.00	0.00	0.98	0.02	0.00	0.00	0.00	0.00	0.00
2	12	FA	16	ON	0.08	0.06	0.57	0.12	0.07	0.05	0.01	0.03	0.01
2	13	FB	16	OFF	0.00	0.00	0.96	0.03	0.00	0.00	0.00	0.00	0.00
2	13	FB	16	ON	0.08	0.04	0.50	0.15	0.10	0.06	0.02	0.04	0.01
2	14	S	16	OFF	0.00	0.00	0.94	0.04	0.00	0.01	0.01	0.00	0.00
2	14	S	16	ON	0.05	0.02	0.48	0.14	0.10	0.06	0.10	0.05	0.01
2	15	FC	16	OFF	0.00	0.00	0.98	0.02	0.00	0.00	0.00	0.00	0.00
2	15	FC	16	ON	0.12	0.03	0.48	0.12	0.12	0.07	0.02	0.03	0.01
2	16	FA	16	OFF	0.00	0.00	1.00	0.00	0.00	0.00	0.00	0.00	0.00
2	16	FA	16	ON	0.06	0.03	0.62	0.14	0.09	0.02	0.01	0.02	0.01
2	2	FB	16	OFF	0.00	0.00	0.99	0.01	0.00	0.00	0.00	0.00	0.00
2	2	FB	16	ON	0.10	0.03	0.33	0.16	0.19	0.14	0.03	0.02	0.00
2	3	S	16	OFF	0.00	0.00	0.99	0.01	0.00	0.00	0.00	0.00	0.00
2	3	S	16	ON	0.08	0.03	0.32	0.14	0.23	0.08	0.09	0.04	0.00
2	4	FC	16	OFF	0.00	0.00	1.00	0.00	0.00	0.00	0.00	0.00	0.00
2	4	FC	16	ON	0.12	0.02	0.45	0.12	0.16	0.09	0.01	0.01	0.00
2	5	FA	16	OFF	0.00	0.00	1.00	0.00	0.00	0.00	0.00	0.00	0.00
2	5	FA	16	ON	0.13	0.03	0.43	0.12	0.13	0.12	0.01	0.02	0.00
2	6	FB	16	OFF	0.00	0.00	0.99	0.01	0.00	0.00	0.00	0.00	0.00
2	6	FB	16	ON	0.11	0.03	0.46	0.15	0.12	0.09	0.02	0.03	0.00
2	7	S	16	OFF	0.00	0.00	1.00	0.00	0.00	0.00	0.00	0.00	0.00
2	7	S	16	ON	0.10	0.03	0.42	0.11	0.17	0.10	0.05	0.02	0.00
2	8	FC	16	OFF	0.00	0.00	0.98	0.02	0.00	0.00	0.00	0.00	0.00
2	8	FC	16	ON	0.10	0.03	0.45	0.07	0.20	0.11	0.01	0.03	0.00
2	9	FB	16	OFF	0.00	0.01	0.97	0.02	0.00	0.00	0.00	0.00	0.00
2	9	FB	16	ON	0.13	0.05	0.49	0.10	0.12	0.06	0.03	0.03	0.00
2	1	FA	23	OFF	0.00	0.00	0.99	0.01	0.00	0.00	0.00	0.00	0.00
2	1	FA	23	ON	0.07	0.04	0.66	0.10	0.03	0.05	0.01	0.04	0.00
2	10	S	23	OFF	0.00	0.00	0.85	0.03	0.00	0.00	0.12	0.00	0.00
2	10	S	23	ON	0.03	0.01	0.55	0.13	0.08	0.06	0.08	0.04	0.01
2	11	FC	23	OFF	0.00	0.00	0.99	0.01	0.00	0.00	0.00	0.00	0.00
2	11	FC	23	ON	0.08	0.03	0.63	0.10	0.07	0.03	0.02	0.04	0.00
2	12	FA	23	OFF	0.00	0.00	1.00	0.00	0.00	0.00	0.00	0.00	0.00
2	12	FA	23	ON	0.08	0.03	0.65	0.13	0.04	0.03	0.01	0.02	0.00
2	13	FB	23	OFF	0.00	0.00	0.97	0.03	0.00	0.00	0.00	0.00	0.00
2	13	FB	23	ON	0.06	0.01	0.61	0.12	0.10	0.04	0.02	0.05	0.00
2	14	S	23	OFF	0.00	0.00	0.88	0.10	0.00	0.02	0.01	0.00	0.00
2	14	S	23	ON	0.05	0.02	0.46	0.16	0.13	0.04	0.11	0.03	0.00
2	15	FC	23	OFF	0.00	0.00	0.99	0.00	0.00	0.00	0.00	0.00	0.00
2	15	FC	23	ON	0.11	0.02	0.59	0.10	0.07	0.06	0.01	0.04	0.00
2	16	FA	23	OFF	0.00	0.00	0.96	0.03	0.00	0.00	0.00	0.00	0.00

2	16	FA	23	ON	0.07	0.02	0.61	0.15	0.06	0.03	0.01	0.05	0.01
2	2	FB	23	OFF	0.00	0.00	0.97	0.03	0.00	0.00	0.00	0.00	0.00
2	2	FB	23	ON	0.06	0.02	0.57	0.14	0.08	0.07	0.02	0.04	0.01
2	3	S	23	OFF	0.00	0.00	0.97	0.01	0.00	0.00	0.02	0.00	0.00
2	3	S	23	ON	0.04	0.02	0.49	0.13	0.08	0.05	0.14	0.04	0.01
2	4	FC	23	OFF	0.00	0.00	0.98	0.02	0.00	0.00	0.00	0.00	0.00
2	4	FC	23	ON	0.09	0.03	0.63	0.09	0.06	0.06	0.01	0.03	0.00
2	5	FA	23	OFF	0.00	0.00	0.99	0.01	0.00	0.00	0.00	0.00	0.00
2	5	FA	23	ON	0.08	0.03	0.68	0.08	0.06	0.04	0.01	0.03	0.00
2	6	FB	23	OFF	0.00	0.00	0.98	0.02	0.00	0.00	0.00	0.00	0.00
2	6	FB	23	ON	0.08	0.03	0.61	0.11	0.09	0.04	0.02	0.03	0.00
2	7	S	23	OFF	0.00	0.00	0.96	0.02	0.00	0.00	0.02	0.00	0.00
2	7	S	23	ON	0.03	0.02	0.34	0.19	0.16	0.10	0.11	0.04	0.01
2	8	FC	23	OFF	0.00	0.00	0.93	0.01	0.00	0.00	0.06	0.00	0.00
2	8	FC	23	ON	0.07	0.03	0.65	0.08	0.06	0.04	0.03	0.04	0.00
2	9	FB	23	OFF	0.00	0.00	0.98	0.02	0.00	0.00	0.00	0.00	0.00
2	9	FB	23	ON	0.08	0.03	0.63	0.11	0.06	0.04	0.01	0.03	0.00
2	1	FA	30	OFF	0.00	0.00	0.96	0.03	0.00	0.00	0.01	0.00	0.00
2	1	FA	30	ON	0.07	0.02	0.73	0.08	0.04	0.02	0.00	0.03	0.00
2	10	S	30	OFF	0.00	0.00	0.90	0.01	0.00	0.00	0.08	0.00	0.00
2	10	S	30	ON	0.05	0.02	0.49	0.10	0.10	0.06	0.12	0.06	0.00
2	11	FC	30	OFF	0.00	0.00	0.99	0.01	0.00	0.00	0.00	0.00	0.00
2	11	FC	30	ON	0.05	0.04	0.71	0.05	0.05	0.03	0.02	0.05	0.00
2	12	FA	30	OFF	0.00	0.00	0.98	0.02	0.00	0.00	0.00	0.00	0.00
2	12	FA	30	ON	0.07	0.04	0.71	0.06	0.04	0.02	0.00	0.04	0.00
2	13	FB	30	OFF	0.00	0.00	0.99	0.01	0.00	0.00	0.00	0.00	0.00
2	13	FB	30	ON	0.06	0.02	0.73	0.04	0.06	0.02	0.01	0.06	0.00
2	14	S	30	OFF	0.00	0.00	0.89	0.03	0.00	0.00	0.08	0.00	0.00
2	14	S	30	ON	0.05	0.01	0.51	0.14	0.05	0.03	0.15	0.05	0.00
2	15	FC	30	OFF	0.00	0.00	0.99	0.01	0.00	0.00	0.00	0.00	0.00
2	15	FC	30	ON	0.07	0.03	0.61	0.06	0.09	0.04	0.05	0.05	0.00
2	16	FA	30	OFF	0.00	0.00	0.99	0.01	0.00	0.00	0.00	0.00	0.00
2	16	FA	30	ON	0.06	0.03	0.75	0.08	0.03	0.01	0.00	0.04	0.00
2	2	FB	30	OFF	0.00	0.00	0.97	0.03	0.00	0.00	0.00	0.00	0.00
2	2	FB	30	ON	0.06	0.03	0.69	0.06	0.06	0.03	0.00	0.06	0.00
2	3	S	30	OFF	0.00	0.00	0.92	0.02	0.00	0.00	0.06	0.00	0.00
2	3	S	30	ON	0.03	0.02	0.51	0.10	0.08	0.04	0.14	0.07	0.01
2	4	FC	30	OFF	0.00	0.00	0.96	0.04	0.00	0.00	0.00	0.00	0.00
2	4	FC	30	ON	0.08	0.03	0.66	0.08	0.06	0.03	0.00	0.06	0.00
2	5	FA	30	OFF	0.00	0.00	0.98	0.02	0.00	0.00	0.00	0.00	0.00
2	5	FA	30	ON	0.07	0.04	0.71	0.06	0.04	0.03	0.00	0.05	0.00
2	6	FB	30	OFF	0.00	0.00	0.98	0.02	0.00	0.00	0.00	0.00	0.00
2	6	FB	30	ON	0.08	0.03	0.62	0.08	0.09	0.04	0.01	0.05	0.01
2	7	S	30	OFF	0.00	0.00	0.93	0.01	0.00	0.00	0.06	0.00	0.00
2	7	S	30	ON	0.04	0.01	0.45	0.12	0.12	0.05	0.13	0.07	0.01
2	8	FC	30	OFF	0.00	0.00	0.94	0.04	0.00	0.00	0.02	0.00	0.00
2	8	FC	30	ON	0.06	0.02	0.71	0.05	0.06	0.01	0.01	0.06	0.01
2	9	FB	30	OFF	0.00	0.00	0.99	0.01	0.00	0.00	0.00	0.00	0.00
2	9	FB	30	ON	0.06	0.03	0.65	0.09	0.07	0.03	0.00	0.05	0.00
2	1	FA	37	OFF	0.00	0.00	0.99	0.01	0.00	0.00	0.00	0.00	0.00
2	1	FA	37	ON	0.08	0.03	0.76	0.03	0.02	0.02	0.00	0.05	0.00
2	10	S	37	OFF	0.00	0.00	0.83	0.03	0.00	0.00	0.14	0.00	0.00
2	10	S	37	ON	0.03	0.01	0.55	0.07	0.09	0.05	0.11	0.09	0.00
2	11	FC	37	OFF	0.00	0.00	0.97	0.02	0.00	0.00	0.00	0.00	0.00
2	11	FC	37	ON	0.06	0.04	0.70	0.05	0.05	0.02	0.00	0.07	0.01
2	12	FA	37	OFF	0.00	0.00	0.96	0.04	0.00	0.00	0.00	0.00	0.00
2	12	FA	37	ON	0.06	0.05	0.72	0.04	0.04	0.02	0.00	0.06	0.00
2	13	FB	37	OFF	0.00	0.00	0.96	0.02	0.00	0.00	0.02	0.00	0.00
2	13	FB	37	ON	0.06	0.02	0.69	0.05	0.06	0.02	0.01	0.09	0.00
2	14	S	37	OFF	0.00	0.00	0.82	0.03	0.00	0.00	0.15	0.00	0.00
2	14	S	37	ON	0.03	0.01	0.51	0.10	0.08	0.06	0.11	0.08	0.01
2	15	FC	37	OFF	0.00	0.00	0.98	0.01	0.00	0.00	0.01	0.00	0.00
2	15	FC	37	ON	0.07	0.03	0.68	0.04	0.06	0.04	0.01	0.06	0.00
2	16	FA	37	OFF	0.00	0.00	0.96	0.04	0.00	0.00	0.00	0.00	0.00
2	16	FA	37	ON	0.06	0.04	0.73	0.04	0.03	0.02	0.00	0.07	0.00
2	2	FB	37	OFF	0.00	0.00	0.98	0.02	0.00	0.00	0.00	0.00	0.00
2	2	FB	37	ON	0.07	0.02	0.73	0.03	0.06	0.02	0.00	0.07	0.00
2	3	S	37	OFF	0.00	0.00	0.86	0.04	0.00	0.00	0.10	0.00	0.00
2	3	S	37	ON	0.04	0.02	0.52	0.09	0.04	0.07	0.13	0.09	0.01
2	4	FC	37	OFF	0.00	0.00	0.98	0.02	0.00	0.00	0.00	0.00	0.00
2	4	FC	37	ON	0.06	0.03	0.70	0.07	0.05	0.02	0.00	0.05	0.00
2	5	FA	37	OFF	0.00	0.00	0.98	0.02	0.00	0.00	0.00	0.00	0.00
2	5	FA	37	ON	0.05	0.03	0.74	0.05	0.03	0.03	0.00	0.06	0.00
2	6	FB	37	OFF	0.00	0.00	0.99	0.01	0.00	0.00	0.00	0.00	0.00
2	6	FB	37	ON	0.08	0.04	0.72	0.04	0.03	0.02	0.00	0.07	0.00
2	7	S	37	OFF	0.00	0.00	0.93	0.00	0.00	0.00	0.07	0.00	0.00

2	7	S	37	ON	0.04	0.02	0.48	0.09	0.09	0.07	0.11	0.09	0.01
2	8	FC	37	OFF	0.00	0.00	0.95	0.04	0.00	0.00	0.00	0.00	0.00
2	8	FC	37	ON	0.04	0.03	0.73	0.06	0.05	0.02	0.00	0.07	0.00
2	9	FB	37	OFF	0.00	0.00	0.96	0.03	0.00	0.00	0.00	0.00	0.00
2	9	FB	37	ON	0.06	0.03	0.69	0.05	0.06	0.03	0.00	0.07	0.00
