# Supplementary material for: Slow and steady wins the race: The behaviour and welfare of commercial faster growing broiler breeds compared to a commercial slower growing breed
Source: PLoS One. 2020 Apr 6;15(4):e0231006. doi: 10.1371/journal.pone.0231006 (PMC7135253; doi:10.1371/journal.pone.0231006)
Supplement: S11 Data — (PDF) [file pone.0231006.s011.pdf]

Replicate	Pen	Breed	Phase	feed per 50 birds (kg)
1	1	FB	Starter	14.04
1	1	FB	Grower	87.18
1	1	FB	Finisher	108.42
1	2	FA	Starter	16.85
1	2	FA	Grower	97.46
1	2	FA	Finisher	101.18
1	3	FC	Starter	13.42
1	3	FC	Grower	86.14
1	3	FC	Finisher	106.64
1	4	S	Starter	10.50
1	4	S	Grower	56.90
1	4	S	Finisher	192.34
1	5	FB	Starter	13.77
1	5	FB	Grower	91.46
1	5	FB	Finisher	108.61
1	6	FA	Starter	15.63
1	6	FA	Grower	96.64
1	6	FA	Finisher	99.60
1	7	FC	Starter	12.71
1	7	FC	Grower	82.94
1	7	FC	Finisher	106.12
1	8	S	Starter	9.65
1	8	S	Grower	57.82
1	8	S	Finisher	190.06
1	9	S	Starter	12.12
1	9	S	Grower	60.15
1	9	S	Finisher	196.42
1	10	FB	Starter	14.18
1	10	FB	Grower	91.94
1	10	FB	Finisher	107.46
1	11	FA	Starter	14.77
1	11	FA	Grower	94.94
1	11	FA	Finisher	97.57
1	12	FC	Starter	12.39
1	12	FC	Grower	88.82
1	12	FC	Finisher	106.41
1	13	S	Starter	11.64
1	13	S	Grower	60.54
1	13	S	Finisher	191.49
1	14	FB	Starter	13.11
1	14	FB	Grower	88.85
1	14	FB	Finisher	108.23
1	15	FA	Starter	14.42
1	15	FA	Grower	95.54
1	15	FA	Finisher	100.49
1	16	FC	Starter	13.53
1	16	FC	Grower	89.14
1	16	FC	Finisher	105.33
2	1	FA	Starter	15.46
2	1	FA	Grower	96.65
2	1	FA	Finisher	124.39
2	2	FB	Starter	12.17

2	2	FB	Grower	91.28
2	2	FB	Finisher	122.76
2	3	S	Starter	11.16
2	3	S	Grower	65.96
2	3	S	Finisher	195.65
2	4	FC	Starter	11.91
2	4	FC	Grower	83.43
2	4	FC	Finisher	120.55
2	5	FA	Starter	16.08
2	5	FA	Grower	105.15
2	5	FA	Finisher	125.84
2	6	FB	Starter	12.98
2	6	FB	Grower	96.91
2	6	FB	Finisher	124.41
2	7	S	Starter	11.71
2	7	S	Grower	62.99
2	7	S	Finisher	202.11
2	8	FC	Starter	11.51
2	8	FC	Grower	91.73
2	8	FC	Finisher	122.82
2	9	FB	Starter	13.26
2	9	FB	Grower	90.67
2	9	FB	Finisher	120.94
2	10	S	Starter	11.60
2	10	S	Grower	66.91
2	10	S	Finisher	200.45
2	11	FC	Starter	11.49
2	11	FC	Grower	79.59
2	11	FC	Finisher	118.98
2	12	FA	Starter	14.27
2	12	FA	Grower	99.10
2	12	FA	Finisher	118.16
2	13	FB	Starter	12.93
2	13	FB	Grower	92.40
2	13	FB	Finisher	122.72
2	14	S	Starter	12.33
2	14	S	Grower	62.59
2	14	S	Finisher	193.89
2	15	FC	Starter	12.26
2	15	FC	Grower	88.43
2	15	FC	Finisher	123.15
2	16	FA	Starter	16.00
2	16	FA	Grower	108.20
2	16	FA	Finisher	126.68
