# Supplementary material for: Slow and steady wins the race: The behaviour and welfare of commercial faster growing broiler breeds compared to a commercial slower growing breed
Source: PLoS One. 2020 Apr 6;15(4):e0231006. doi: 10.1371/journal.pone.0231006 (PMC7135253; doi:10.1371/journal.pone.0231006)
Supplement: S12 Data — (PDF) [file pone.0231006.s012.pdf]

Replicate	Pen	Breed	FCR	ADG
1	1	FB	1.50	60.54
1	2	FA	1.47	64.27
1	3	FC	1.33	64.10
1	4	S	1.79	45.13
1	5	FB	1.49	61.70
1	6	FA	1.44	64.02
1	7	FC	1.32	62.91
1	8	S	1.75	45.30
1	9	S	1.73	47.08
1	10	FB	1.50	61.99
1	11	FA	1.44	62.74
1	12	FC	1.37	63.55
1	13	S	1.73	47.07
1	14	FB	1.47	61.40
1	15	FA	1.48	62.16
1	16	FC	1.38	63.23
2	1	FA	1.39	62.56
2	2	FB	1.37	62.59
2	3	S	1.71	46.32
2	4	FC	1.31	62.91
2	5	FA	1.48	64.43
2	6	FB	1.39	64.84
2	7	S	1.81	46.37
2	8	FC	1.39	62.98
2	9	FB	1.32	64.91
2	10	S	1.82	45.98
2	11	FC	1.29	60.99
2	12	FA	1.43	62.98
2	13	FB	1.38	62.89
2	14	S	1.77	45.41
2	15	FC	1.39	61.36
2	16	FA	1.52	64.78
