# Supplementary material for: Slow and steady wins the race: The behaviour and welfare of commercial faster growing broiler breeds compared to a commercial slower growing breed
Source: PLoS One. 2020 Apr 6;15(4):e0231006. doi: 10.1371/journal.pone.0231006 (PMC7135253; doi:10.1371/journal.pone.0231006)
Supplement: S13 Data — (PDF) [file pone.0231006.s013.pdf]

Breed	Sex	Evis carcass (g	Ab Fat (g	Breast Weights	Leg Weights (g)
FB	F	1724	37	506	533
FB	M	2064	31	597	654
FA	F	1903	39	604	563
FA	M	2315	36	736	691
FC	F	1818	32	567	552
FC	M	2158	33	659	677
FB	F	1760	38	528	541
FB	M	2122	35	613	665
FA	F	1914	38	598	573
FA	M	2346	33	727	685
FC	F	1780	34	540	548
FC	M	2166	36	649	667
FB	F	1789	41	520	565
FB	M	2135	34	611	689
FA	F	1858	37	591	548
FA	M	2232	34	689	671
FC	F	1840	35	573	572
FC	M	2188	33	663	702
FB	F	1718	35	493	544
FB	M	2014	35	558	644
FA	F	1821	33	587	539
FA	M	2293	34	714	692
FC	F	1869	35	578	577
FC	M	2144	34	650	670
S	F	1885	73	461	584
S	M	2450	77	565	802
S	F	1946	70	480	609
S	M	2441	76	578	802
