# Supplementary material for: Slow and steady wins the race: The behaviour and welfare of commercial faster growing broiler breeds compared to a commercial slower growing breed
Source: PLoS One. 2020 Apr 6;15(4):e0231006. doi: 10.1371/journal.pone.0231006 (PMC7135253; doi:10.1371/journal.pone.0231006)
Supplement: S14 Data — (PDF) [file pone.0231006.s014.pdf]

Breed	Sex	Breast Stri	Prop
FB	F	0	0.33
FB	M	0	0.15
FA	F	0	0.25
FA	M	0	0.00
FC	F	0	0.27
FC	M	0	0.15
FB	F	0	0.24
FB	M	0	0.15
FA	F	0	0.36
FA	M	0	0.29
FC	F	0	0.52
FC	M	0	0.26
FB	F	0	0.29
FB	M	0	0.14
FA	F	0	0.26
FA	M	0	0.04
FC	F	0	0.52
FC	M	0	0.24
FB	F	0	0.18
FB	M	0	0.23
FA	F	0	0.05
FA	M	0	0.17
FC	F	0	0.42
FC	M	0	0.27
S	F	0	0.96
S	M	0	0.81
S	F	0	0.88
S	M	0	0.96
FB	F	1	0.57
FB	M	1	0.65
FA	F	1	0.54
FA	M	1	0.74
FC	F	1	0.64
FC	M	1	0.81
FB	F	1	0.57
FB	M	1	0.74
FA	F	1	0.57
FA	M	1	0.53
FC	F	1	0.40
FC	M	1	0.68
FB	F	1	0.54
FB	M	1	0.76
FA	F	1	0.57
FA	M	1	0.61
FC	F	1	0.39
FC	M	1	0.64
FB	F	1	0.59
FB	M	1	0.69
FA	F	1	0.81
FA	M	1	0.67
FC	F	1	0.46
FC	M	1	0.55

S	F	1	0.04
S	M	1	0.15
S	F	1	0.12
S	M	1	0.04
FB	F	2	0.10
FB	M	2	0.20
FA	F	2	0.11
FA	M	2	0.21
FC	F	2	0.05
FC	M	2	0.04
FB	F	2	0.19
FB	M	2	0.11
FA	F	2	0.04
FA	M	2	0.18
FC	F	2	0.04
FC	M	2	0.00
FB	F	2	0.18
FB	M	2	0.10
FA	F	2	0.09
FA	M	2	0.35
FC	F	2	0.04
FC	M	2	0.12
FB	F	2	0.18
FB	M	2	0.08
FA	F	2	0.10
FA	M	2	0.13
FC	F	2	0.08
FC	M	2	0.14
S	F	2	0.00
S	M	2	0.04
S	F	2	0.00
S	M	2	0.00
