# Supplementary material for: Slow and steady wins the race: The behaviour and welfare of commercial faster growing broiler breeds compared to a commercial slower growing breed
Source: PLoS One. 2020 Apr 6;15(4):e0231006. doi: 10.1371/journal.pone.0231006 (PMC7135253; doi:10.1371/journal.pone.0231006)
Supplement: S15 Data — (PDF) [file pone.0231006.s015.pdf]

Breed	Sex	Wooden	Prop
FB	F	0	1.00
FB	M	0	0.95
FA	F	0	0.82
FA	M	0	0.58
FC	F	0	0.86
FC	M	0	0.58
FB	F	0	0.95
FB	M	0	0.93
FA	F	0	0.89
FA	M	0	0.59
FC	F	0	0.92
FC	M	0	0.74
FB	F	0	1.00
FB	M	0	1.00
FA	F	0	0.74
FA	M	0	0.61
FC	F	0	0.91
FC	M	0	0.84
FB	F	0	0.95
FB	M	0	0.92
FA	F	0	0.86
FA	M	0	0.67
FC	F	0	0.92
FC	M	0	0.82
S	F	0	1.00
S	M	0	0.96
S	F	0	1.00
S	M	0	1.00
FB	F	1	0.00
FB	M	1	0.05
FA	F	1	0.07
FA	M	1	0.26
FC	F	1	0.09
FC	M	1	0.31
FB	F	1	0.05
FB	M	1	0.07
FA	F	1	0.04
FA	M	1	0.18
FC	F	1	0.04
FC	M	1	0.05
FB	F	1	0.00
FB	M	1	0.00
FA	F	1	0.13
FA	M	1	0.26
FC	F	1	0.04
FC	M	1	0.12
FB	F	1	0.00
FB	M	1	0.08
FA	F	1	0.10
FA	M	1	0.17
FC	F	1	0.04
FC	M	1	0.09

S	F	1	0.00
S	M	1	0.04
S	F	1	0.00
S	M	1	0.00
FB	F	2	0.00
FB	M	2	0.00
FA	F	2	0.00
FA	M	2	0.11
FC	F	2	0.00
FC	M	2	0.12
FB	F	2	0.00
FB	M	2	0.00
FA	F	2	0.04
FA	M	2	0.24
FC	F	2	0.00
FC	M	2	0.16
FB	F	2	0.00
FB	M	2	0.00
FA	F	2	0.04
FA	M	2	0.13
FC	F	2	0.00
FC	M	2	0.04
FB	F	2	0.00
FB	M	2	0.00
FA	F	2	0.00
FA	M	2	0.13
FC	F	2	0.00
FC	M	2	0.05
S	F	2	0.00
S	M	2	0.00
S	F	2	0.00
S	M	2	0.00
